# Governing the emissive properties of 4-aminobiphenyl-2-pyrimidine push–pull systems via the restricted torsion of N,N-disubstituted amino groups

**DOI:** 10.3389/fchem.2023.1292541

**Published:** 2023-11-10

**Authors:** Alejandro Cortés-Villena, Iván Soriano-Díaz, Moisés Domínguez, Matías Vidal, Pablo Rojas, Carolina Aliaga, Angelo Giussani, Antonio Doménech-Carbó, Enrique Ortí, Raquel E. Galian, Julia Pérez-Prieto

**Affiliations:** ^1^ Instituto de Ciencia Molecular, Universidad de Valencia, Valencia, Spain; ^2^ Facultad de Química y Biología, Universidad de Santiago de Chile, Santiago, Chile; ^3^ Centro para el Desarrollo de la Nanociencia y la Nanotecnología (CEDENNA), Universidad de Santiago de Chile, Santiago, Chile; ^4^ Departamento de Química Analítica, Universidad de Valencia, Valencia, Spain

**Keywords:** intramolecular charge transfer, donor–acceptor systems, fluorosolvatochromism, photophysical properties, theoretical calculations

## Abstract

Donor–acceptor-substituted biphenyl derivatives are particularly interesting model compounds, which exhibit intramolecular charge transfer because of the extent of charge transfer between both substituents. The connection of a 4-[1,1′-biphenyl]-4-yl-2-pyrimidinyl) moiety to differently disubstituted amino groups at the biphenyl terminal can offer push–pull compounds with distinctive photophysical properties. Herein, we report a comprehensive study of the influence of the torsion angle of the disubstituted amino group on the emissive properties of two pull–push systems: 4-[4-(4-*N*,*N*-dimethylaminophenyl)phenyl]-2,6-diphenylpyrimidine (**D1**) and 4-[4-(4-*N*,*N*-diphenylaminophenyl)phenyl]-2,6-diphenylpyrimidine (**D2**). The torsion angle of the disubstituted amino group, either *N*,*N*-dimethyl-amine or *N*,*N*-diphenyl-amine, at the biphenyl end governs their emissive properties. A drastic fluorescence quenching occurs in **D1** as the solvent polarity increases, whereas **D2** maintains its emission independently of the solvent polarity. Theoretical calculations on **D1** support the presence of a twisted geometry for the lowest energy, charge-transfer excited state (S_1,90_), which corresponds to the minimum energy structure in polar solvents and presents a small energy barrier to move from the excited to the ground state, thereby favoring the non-radiative pathway and reducing the fluorescence efficiency. In contrast, this twisted structure is absent in **D2** due to the steric hindrance of the phenyl groups attached to the amine group, making the non-radiative decay less favorable. Our findings provide insights into the crucial role of the substituent in the donor moiety of donor–acceptor systems on both the singlet excited state and the intramolecular charge-transfer process.

## Introduction

Push–pull structures are conjugated organic molecules integrated by electron-donating and electron-withdrawing moieties separated by a π-system, which broadens the charge distribution across the molecule, endowing it with exotic optical and electronic properties (Bureš, 2014). This class of materials has been extensively used as sensitizers in dye-sensitized solar cells (DSSCs) (Verbitskiy et al., 2014; Verbitskiy et al., 2015; Tan et al., 2016; Kozlov et al., 2017; Sun et al., 2018; Verbitskiy et al., 2021b) and hole-transporting materials in perovskite-based solar cells (Maddala et al., 2021; Bouihi et al., 2022; Manda et al., 2022) owing to their high molar absorption coefficient and efficient hole mobility, respectively. Moreover, they can be integrated into organic light-emitting diodes (OLEDs) (Wong et al., 2002; Wu et al., 2002; Nakao et al., 2017; Ryutaro et al., 2018; Verbitskiy et al., 2021a) because of their high fluorescence quantum yields (*Φ*_F_).

Pyrimidine derivatives have been used as the electron-withdrawing group in push–pull systems due to the significant π-deficient character of diazine rings (Bureš, 2014; Achelle et al., 2023). This character of the pyrimidine ring can be further increased by protonation, complexation, or alkylation of the nitrogen electron lone pair. Pyrimidine derivatives substituted with electron-donating fragments through π-conjugated linkers are highly fluorescent and sensitive to external stimuli (Achelle et al., 2023).

In general, push–pull organic chromophores play a strategic role in the development of new and sophisticated applications in photonics (Verbitskiy et al., 2021b). The optimization of the organic structure with the appropriate design of a π-electron structure has made it possible to bring these systems closer to the market. Thus, it is possible to exploit the unique properties of push–pull benzenoid derivatives and heterocyclic rings to obtain novel systems that can efficiently convert the emission of a cheap, easily available IR laser into that of a more technological valuable visible laser. Exploiting substituent effects and properly adjusting the π-electron structure can not only modulate the emission frequency, so that a whole range of laser wavelengths is accessible, but also improve upconversion efficiencies to meet market requirements (Fecková et al., 2020).

Particularly, push–pull organic systems exhibit strong fluorosolvatochromism as a consequence of the large dipole moment in the excited state (Hadad et al., 2011; Verbitskiy et al., 2014; Liu et al., 2022), thus making them interesting candidates for their application as chemical and biochemical environmental probes (Qin et al., 2021). The large Stokes shift observed upon increasing solvent polarity stems from an intramolecular relaxation process in an electronic excited state, which sometimes leads to a new energetic minimum far below the former structure in the excited state. This relaxation process typically accompanies not only changes in bond lengths and bond angles but also structural changes due to rotation around a single bond (Haberhauer, 2017).

The importance of electron-donor and acceptor groups, conjugation in the excited state, and the nature of π-bridges (particularly thiophene) has been investigated in the literature for indolo [2,3-b]quinoxaline-based dyes, aryl-substituted indolo [2,3-a]carbazole derivatives synthesized and indeno [1,2-b]indole donor derivatives (Venkateswararao et al., 2014; Yang et al., 2015; Qian et al., 2017). The solvent polarity effect has also been reported for a large number of organic fluorophores, such as tetrazole-substituted pyrene and carbazole-substituted quinoline dyes (Slodek et al., 2019; Zych et al., 2019). Complex systems based on the D–D–π–A architecture such as new indolo [3,2,1-jk]carbazole derivatives have also been reported by Schab-Balcerzak, E. et al. for dye-sensitized solar cells. A phenothiazine unit and an acetylene linkage containing either an aldehyde or cyanoacrylic acid as electron-withdrawing groups were used, and a significant solvent effect was only observed for the cyanoacrylic acid, demonstrating the high sensitivity of the ICT state to the electronic properties of the linkage group (Gnida et al., 2022). Another D–π–D–π–A architecture reported by Schab-Balcerzak, E. et al. using a phenothiazine-based cyanoacrylic acid containing an imidazole ring substituted with the alkyl group with different chain lengths was employed. Time-resolved fluorescence studies were performed using different solvent polarities, indicating the presence of an ICT state more stabilized in polar solvents such as DMF (Zimosz et al., 2022).

The structure–fluorosolvatochromism relationship of pyrimidine-based chromophores has already been reported from an experimental point of view and using Taguchi methodology (Denneval et al., 2014; Achelle and Robin-le Guen, 2017). As a benchmark molecule that undergoes such a process, 4-(dimethylamino)benzonitrile (DMABN) has been widely used to disentangle empirical findings from a theoretical perspective. Yet, several models have been proposed for the explanation of the lower energy band exhibited by this relatively simple molecule; however, they are still under debate (Grabowski et al., 1979; Zachariasse et al., 1996; Gómez et al., 2021). First, the twisted intramolecular charge-transfer (TICT) model was proposed by Grabowski et al. to elucidate the dual fluorescence (from locally excited and intramolecular charge-transfer states) observed for DMABN in polar solvents (Grabowski et al., 1979; Grabowski et al., 2003; Sasaki et al., 2016). This model states that after the electronic excitation of DMABN in polar solvents, a charge transfer concomitantly with a ca. 90° twist of the single bond occurs, thereby electronically disconnecting the donor and acceptor moieties in the excited state. The resulting charge-transfer state is more stable than the preceding localized state (Galván et al., 2010; Segarra-Martí and Coto, 2014). The driving force for this sort of stabilization emerges from the minimization of the Coulomb interaction of the two unpaired electrons. Since the rotation leads to π-orbital decoupling, the fluorescence associated with a TICT process is typically weakened and redshifted (Haberhauer, 2017). On the other hand, Zachariasse et al. proposed the planarized intramolecular charge-transfer (PLICT) model to explain the opposite effect in which a rotation of ca. 90° leads to planarization of the donor and acceptor moieties in the excited state instead (Zachariasse et al., 1996; Zachariasse et al., 1997; Il’ichev et al., 1998). A key difference compared to the TICT process is that the fluorescence from a PLICT state to the ground state is now allowed, and thus high quantum yields are expected (Haberhauer et al., 2016).

Consequently, given the vast amount of the literature on DMABN as a model molecule that is still under discussion, we were motivated to shed light on the mechanism underlying fluorosolvatochromism that occurs in biphenylpyrimidine derivatives. The impact of the donor substituent on the optical and excited state properties of two π-extended biphenylpyrimidines was studied. Particularly, push–pull systems which comprise a dialkylamino or diarylamino and a pyrimidine as electron-donor and -acceptor moieties, respectively, were studied by steady-state and time-resolved fluorescence techniques combined with theoretical calculations that significantly contribute to support the experimental findings.

This research contributes to expanding knowledge about the photochemical behavior and properties of simple push–pull systems and understanding of the relationship between the molecular structure of the amino donor, the solvent environment, and the fluorescence properties. This analysis can help select the right conditions for various applications in chemistry and materials science, such as sensing, imaging, and optoelectronic devices.

## Materials and methods

### Materials

The synthesis and characterization of the respective 2,4,6-triarylpyrimidine derivatives, 4-[4-(4-*N*,*N*-dimethylaminophenyl)phenyl]-2,6-diphenylpyrimidine (**D1**) and 4-[4-(4-*N*,*N*-diphenylaminophenyl)phenyl]-2,6-diphenylpyrimidine (**D2**), by Rodríguez Aguilar and coworkers were reported elsewhere (Rodríguez-Aguilar et al., 2018). In brief, a microwave vial (10 mL) was charged with bromophenyl-4-pyrimidines (0.7 mmol), Pd(PPh_3_)_4_ (41 mg, 0.035 mmol, and 5 mol%), K_2_CO_3_ (97 mg and 0.7 mmol), *N*,*N*-dimethylformamide (5 mL), and 4-(*N*,*N*-dimethyl)phenylboronic acid (139 mg and 0.84 mmol) for **D1** or the 4-(*N*,*N*-diphenyl)phenylboronic acid (243 mg and 0.84 mmol) for **D2**. The resulting reaction mixture was heated for 1 h at 100°C. Upon the end of the reaction (as observed on TLC, n-hexanes/EtOAc, 5:1), the crude was diluted with water (25 mL) and extracted with EtOAc (3 × 15 mL). The combined organic extracts were dried over Na_2_SO_4_, and all the volatile components were removed by rotary evaporation. The respective products were purified by column chromatography (*n*-hexane: EtOAc, 20:1 → 5:1).

All commercially available solvents used for the spectroscopy investigation were purchased from Alfa Aesar and used as received without further purification. The short names of the solvents are included in Table 1.

**TABLE 1:** Optical data (absorption and fluorescence maxima, Stokes shift, and absolute fluorescence quantum yield (*Φ*_F_)) of D1 and D2 systems in solvents with a decreasing dielectric constant (*ε*) under anaerobic conditions. Fluorescence and *Φ*_F_ measurements were recorded upon excitation at 365 nm.

Sample	Solvent, dielectric constant (*ε*)	Abs. max., *λ*_abs_ (nm)	Fluor. max., *λ*_em_ (nm)	Stokes shift, Δ*λ* (nm)	*Φ*_F_ (%)	Nature of the solvent
**D1**	Dimethylsulfoxide (DMSO, *ε* = 46.7)	265 and 376	410, 436, and 609	233	16	Polar aprotic
	Acetonitrile (ACN, *ε* = 37.5)	260 and 349	418 and 593	244	19	
	Dimethylformamide (DMF, *ε* = 36.7)	265 and 373	410, 438, and 588	215	29	
	Methanol (MeOH, *ε* = 32.7)	260 and 361	410, 445, and 608	247	2	Polar protic
	Ethanol (EtOH, *ε* = 24.5)	261 and 364	410 and 595	231	7	
	Dichloromethane (DCM, *ε* = 8.9)	261 and 369	525	156	82	Medium polar
	Ethyl acetate (AcOEt, *ε* = 6.0)	261 and 362	514	152	54	
	Chloroform (CHCl_3_, *ε* = 4.8)	262 and 365	497	132	41	
	Toluene (Tol, *ε* = 2.4)	286 and 365	452	87	87	Non-polar
	Benzene (Bz, *ε* = 2.3)	278 and 365	455	90	96	
	Hexane (Hx, *ε* = 1.9)	259 and 354	403, 425, and 454	49	87	
**D2**	Dimethylsulfoxide (DMSO, *ε* = 46.7)	287, 307, and 374	411 and 574	200	79	Polar aprotic
	Acetonitrile (ACN, *ε* = 37.5)	265, 303, and 365	411 and 574	209	78	
	Dimethylformamide (DMF, *ε* = 36.7)	279, 306, and 372	411 and 559	187	87	
	Methanol (MeOH, *ε* = 32.7)	265, 304, and 365	411, 447, and 600	235	3	Polar protic
	Ethanol (EtOH, *ε* = 24.5)	268, 305, and 367	411 and 571	204	31	
	Dichloromethane (DCM, *ε* = 8.9)	269, 307, and 371	520	149	91	Medium polar
	Ethyl acetate (AcOEt, *ε* = 6.0)	257, 304, and 366	493	127	82	
	Chloroform (CHCl_3_, *ε* = 4.8)	266, 308, and 372	493	121	84	
	Toluene (Tol, *ε* = 2.4)	286, 306, and 373	444	71	96	Non-polar
	Benzene (Bz, *ε* = 2.3)	287, 307, and 371	445	74	69	
	Hexane (Hx, *ε* = 1.9)	267, 303, and 366	411, 434, and 465	45	75	

### Sample purging

A measure of 3 mL contained of optically matched **D1** or **D2** solutions at 0.1 OD at an excitation wavelength (365 nm) was purged with nitrogen directly from the cylinder (to degas air from the sample) for 5 min by placing a syringe needle through the septum into the bottom and another into the air space above the sample as a vent with a flow rate of dry nitrogen so that bubbles are observable in the sample, unless specified. In case, the solvent was evaporated upon bubbling, the cuvettes were refilled with extra purged solvent. Immediately after this procedure, samples were placed in the instrument for measurement. Cuvettes of 1 cm optical path length were used for spectroscopy studies.

### Steady-state UV-vis–NIR absorption spectroscopy

Steady-state UV-vis absorption spectra were recorded on a UV/Vis/NIR PerkinElmer LAMBDA 1050 spectrophotometer equipped with deuterium and tungsten halogen light sources and Peltier-controlled InGaAs and PbS detectors covering from 175 to 3,300 nm. The absorption spectra were collected in the range of 250–700 nm.

### Steady-state photoluminescence spectroscopy

Stationary fluorescence spectra were recorded on a FLS1000 photoluminescence spectrometer (Edinburgh Instruments) equipped with a 450 W ozone-free continuous xenon arc lamp and a photomultiplier (PMT-980) detector in a cooled housing with an extended spectral range of 185–980 nm. A 365 nm excitation wavelength was used in all measurements. The emission range was registered between 380 and 800 nm.

### Photoluminescence quantum yield

Absolute fluorescence quantum yields (*Φ*_F_) were recorded on a FLS1000 photoluminescence spectrometer equipped with an integrating sphere system with a reflectance higher than 99% in the range of 400–1,500 nm. Sample solutions with optical density (OD) of 0.1 at a 365 nm excitation wavelength were used to minimize re-absorption effects. For reference, the neat solvent with the same volume was used.

### Time-resolved photoluminescence spectroscopy

Time-resolved fluorescence measurements were recorded on a FLS1000 photoluminescence spectrometer through the time-correlated single-photon counting (TCSPC) technique coupled with a 375 nm ps pulsed diode laser (EPL-375, pulse width: 75 ps, peak power: 140 mW, and repetition rate: 10 MHz, Edinburgh Instruments) and a microchannel plate (MCP-900) detector in a cooled housing with a spectral range of 200–850 nm in the nanosecond domain. A Ludox solution (0.1 OD at an excitation wavelength) was used as an instrument response function (IRF). The IRF was approximately 110 ps in our setup. All spectra were recorded using a 1-cm-path-length quartz cuvette at room temperature.

### Electrochemical measurements

The characterization of redox properties was performed on an Autolab potentiostat (Autolab 128N potentiostat/galvanostat) using a three-electrode system. Cyclic voltammetry (CV) experiments were carried out in 0.1 M tetrabutylammonium tetrafluoroborate (TBABF_4_) solution in dried acetonitrile (ACN) using Pt as a working electrode, Pt wire as an auxiliary electrode, and Ag/AgCl as a reference electrode separated from the tested solution by means of a Luggin capillary. The measurements were performed at room temperature (298 ± 1 K) partially deaerating the electrolyte solution by bubbling nitrogen for 2 min. Partially oxygenated solutions were optionally used to facilitate the use of the O_2_/O_2_^•-^ couple as the internal standard for electrode potential measurements. The concentration of the compounds was approximately 0.2 mM in dried acetonitrile. Experiments were performed under air conditions, with a scan rate of 100 mV/s. To evaluate the electrochemical bandgaps, potentials were referred to the Fc/Fc^+^ couple using 0.2 mM ferrocene solutions in 0.1 M TBABF_4_/ACN.

### Thermal measurements

The thermal properties of **D1** and **D2** were examined under a nitrogen atmosphere, with a heating rate of 10°C/min up to 950°C.

### Computational details

The singlet ground state (S_0_) was optimized using density functional theory (DFT) calculations. The first excited singlet state (S_1_) was also optimized at the time-dependent DFT (TD-DFT) level of theory. All the calculations were performed based on the exchange-correlation Becke’s three-parameter (B3LYP) functional (Lee et al., 1988; Becke, 1993) and the split-valence triple-zeta 6-311G** basis set (Francl et al., 1982), without imposing any symmetry restriction. Calculations were carried out using Gaussian 16 software (Rev. A.03) (Frisch et al., 2016). Solvent effects (hexane, toluene, ACN, and DMSO) were implicitly considered by employing the polarizable continuum model (PCM) method (Tomasi and Persico, 1994). For this reason, all the excited state geometries were obtained at the LR-PCM TD-DFT level of theory. Nevertheless, solvent effects on the photophysical processes (absorption and emission) can be inadequately reproduced using LR-PCM (Chibani et al., 2014). To solve this problem, single vertical point calculations were employed, at the optimized LR-TD-DFT geometries, using the corrected linear response (CLR-PCM) method (Improta et al., 2006; Improta et al., 2007). This approach allows to relax the interaction between the solvent and the solute molecule in the excited-state minima, thus improving the description of the excited states from an energetical point of view. All the energies discussed in the main text refer to PCM-CLR TD-DFT energies. The optimizations must be carried out at the LR-PCM TD-DFT level of theory because CLR-PCM TD-DFT is only implemented for single-point calculations. The ORCA 5.0.1 code was additionally used (Neese, 2022), employing the same computational approach (B3LYP/6-311G**) and simulating the solvent effects with CPCM methodology (Cammi et al., 2000) to characterize the minimum energy paths (MEPs) connecting two singlet excited-state minima belonging to the same potential energy surface (PES). This was accomplished by employing the climbing image nudged elastic band (CI-NEB) method (Henkelman et al., 2000). The energy of the so-obtained MEPs was then re-computed using Gaussian 16 as described previously. The computation of natural transition orbitals (NTOs) and the Mulliken population analysis were performed as implemented in Gaussian 16 (Martin, 2003; Soriano-Díaz et al., 2023).

## Results and discussion

### Optical and electrochemical properties

**SCHEME 1:**
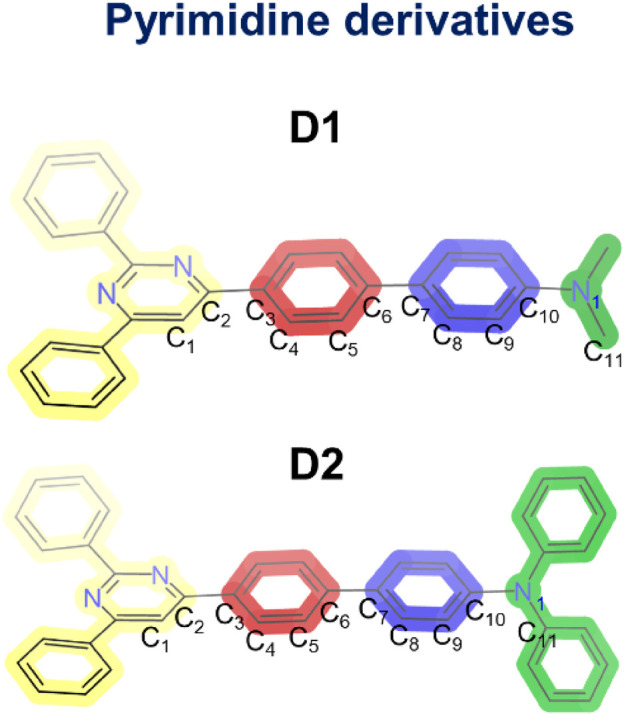
Molecular structures of the two pyrimidine derivatives (**D1** and **D2**) and atomic numbering used to define the dihedral angles in the optimized geometries.

4-[4-(4-*N*,*N*-dimethylaminophenyl)phenyl]-2,6-diphenylpyrimidine (**D1**) and 4-[4-(4-*N*,*N*-diphenylaminophenyl)phenyl]-2,6-diphenylpyrimidine (**D2**) (Scheme 1) were synthesized using previously reported procedures (Rodríguez-Aguilar et al., 2018), and their procedure can be found in the Materials and Methods section. Their optical and photophysical properties were investigated in a battery of solvents with different polarities.

**FIGURE 1:**
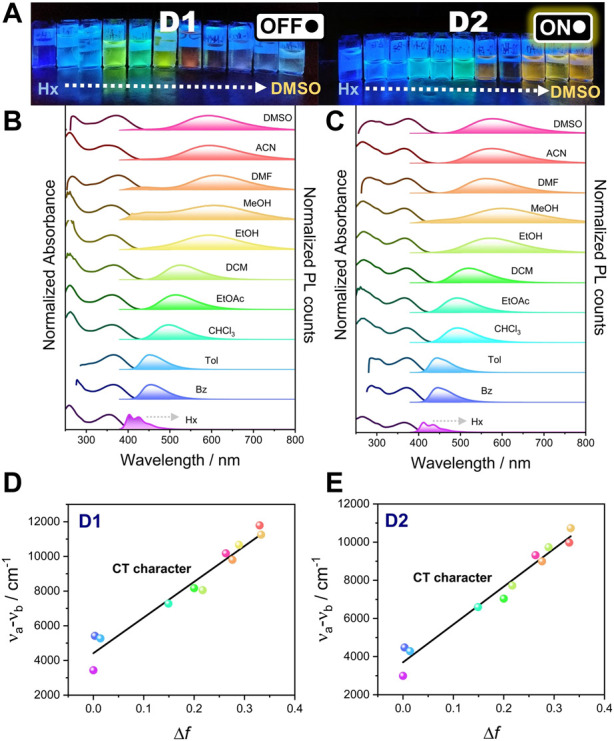
**(A)** Photographs of **D1** and **D2** pyrimidine derivative solutions in solvents with an increasing dielectric constant from hexane (left) to DMSO (right) under UV irradiation (365 nm). Normalized steady-state absorption and fluorescence spectra of **D1 (B)** and **D2 (C)** in the solvents shown previously under anaerobic conditions. From top to bottom, solvents are ordered by decreasing dielectric constant (DMSO, ACN, DMF, MeOH, EtOH, DCM, EtOAc, CHCl_3_, Tol, Bz, and Hx). For fluorescence measurements, an excitation wavelength of 365 nm was used. Lippert–Mataga plot for **D1 (D)** and **D2 (E)**.

The optical properties (steady-state absorption and photoluminescence) of compounds **D1** and **D2** were measured in diluted solutions of 0.1 OD at the excitation wavelength in solvents with an increasing dielectric constant at room temperature and under anaerobic conditions. The recorded absorption and emission spectra are shown in Figure 1, and the relevant optical data are summarized in Table 1. It is evident that these systems exhibit slight differences in the ground-state absorption features, which involve two major absorption bands, namely, 260–285 nm and 355–380 nm for high- and low-energy bands, respectively, and a small shoulder at approximately 303–307 nm for **D2**. The shape and energy of the absorption bands were proved to be weakly dependent on the solvent polarity (Figure 1B).

The shape and energy of the high-energy absorption band in both systems are nearly independent of the solvent polarity, whereas the low-energy band is shown to be weakly dependent on the solvent polarity, being redshifted in DMSO compared to Hx (Δ*λ* is 22 and 18 nm for **D1** and **D2**, respectively), which suggests a low molecular dipole moment in the ground state. However, the observation of a blueshift in polar protic solvents, particularly more intensified in MeOH, is attributed to the hydrogen-bond formation ability (*α*) of alcohol with the molecule in the ground state, which stabilizes this state and leads to an increase in the energy gap as noted previously (Al-Ahmed et al., 2021). Interestingly, in the case of ACN, which is a highly polar solvent, there is also a significant blueshift in the low-energy absorption band compared to DMF, for which the dielectric constant is slightly lower. Then, the observed blueshift is justified by the better hydrogen-bond formation ability of ACN (*α* = 19) than DMF (*α* = 0).

In contrast to the absorption spectra, the fluorescence spectra under a 365 nm excitation wavelength show a strong dependence on the solvent polarity and a remarkable positive solvatochromism upon increasing the solvent dielectric constant. This fluorosolvatochromism suggests a potential intramolecular charge transfer between the donor and acceptor units in the emitting excited state, indicating that the excited state has a larger dipole moment than the ground state. The digital photographs of dye-containing solutions exhibited a wide range of colors, from deep blue to orange. In hexane, the fluorescence spectra of both compounds show that the fingerprint shape were associated with a structured emission as a result of the small solute–solvent interaction (Subuddhi et al., 2006). As the polarity of the solvent increases, the fluorescence spectrum loses its vibrational fine structure, and a broadening and bathochromic shift is observed when moving from hexane to DMSO (Table 1), thus supporting a charge-transfer character of the emitting excited state that explains the large Stokes shift observed (50–250 nm). It is worth mentioning that in polar solvents (from ethanol to DMSO), we observed the appearance of two emission bands, the higher-energy band being more pronounced in **D1** than in **D2** (Figure 1C). The coexistence of two emission bands (small contribution of the higher-energy band) can be rationally understood by the presence of two emitting species as previously observed on the vast number of push–pull systems found in the literature (Fecková et al., 2021). The higher-energy band may be ascribed to the locally excited (LE) state because of the negligible redshift as compared to the fluorescence in hexane. In contrast, the redshifted, higher-intensity emission band is attributed to the charge-transfer emitting state, which proves the large Stokes shift.

To further evaluate this, analysis using the Lippert–Mataga equation was employed in which the Stokes shift (Δ*ν*) is plotted as a function of the orientation polarizability (Δ*f*) of the solvents (Equations 1, 2) (Lippert, 1955; Mataga et al., 1955):

$$\Delta \nu =\nu_{a}-\nu_{b}=\frac{2\Delta f}{hc}\frac{{\left(\mu_{E}-\mu_{G}\right)}^{2}}{a^{3}}+constant,$$ (1)

where *ν*_a_ and *ν*_b_ are the wavenumbers (cm^−1^) of absorption and fluorescence peaks, respectively, *h* is the Planck’s constant, *c* is the speed of light in vacuum, *μ*_E_ and *μ*_G_ are dipole moments in the excited and ground states, respectively, *a* is the radius of the Onsager cavity, and Δ*f* is the orientation polarizability of the solvent given in the following equation:

$$\Delta f=\frac{\varepsilon -1}{2\varepsilon +1}-\frac{\eta^{2}-1}{{2\eta }^{2}+1},$$ (2)

where *ε* and *η* are the dielectric constant and refractive index of the solvent, respectively. The clear linear trend in both compounds (except for hexane) indicates the increase in dipole moment in the excited state compared to the ground state and supports the ICT nature of the excited state (Figures 1D,E). The deviation of the linearity in hexane in both compounds supports the existence of partial contribution of the LE state. A higher slope for **D1** than for **D2** suggests that it exhibits a more pronounced charge-transfer process.

Regarding the emission efficiency, both compounds exhibited high emission *Φ*_F_ in non-polar solvents such as hexane (Hx), toluene (Tol), and benzene (Bz). However, *Φ*_F_ of **D1** was significantly reduced in polar solvents such as dimethylformamide (DMF), acetonitrile (ACN), and dimethylsulfoxide (DMSO), whereas **D2** approximately maintains the high *Φ*_F_ observed in non-polar solvents (see photographs in Figure 1A and values in Table 1).

The solvent-dependent emissive properties shown in Table 1 can be classified according to the dielectric constants as follows: 1) non-polar; 2) medium polar; 3) protic polar; and 4) aprotic polar solvents (Table 1). In non-polar solvents, the fluorescence quantum yield is high because of the negligible non-radiative constants obtained for both **D1** and **D2**. In the case of solvents with intermediate polarity, the solvent polarizability (π*) and dielectric constant (*ε*), could help understand the solvent-dependent photophysical behavior. For example, chloroform and ethyl acetate have π* values of 0.58 and 0.55, respectively, i.e., a π* value similar to toluene (0.54); however, they present an intermediate dielectric constant that deactivates **D1** in a non-radiative pathway, reducing the fluorescence quantum yield. However, DCM with higher *ε* in the block showed a high fluorescence quantum yield for both compounds, which can be attributed to the higher polarizability (0.82) of the solvent (Marcus, 1993).

It has been shown that the *α* values of chloroform and DCM play a key role in the aggregation of molecules in crystal structures and the ability to form H-bonds (Mansoor and Shafi, 2015). Chloroform and DCM present *α* values of 0.44 and 0.30, respectively, confirming the higher H-bond formation ability of chloroform, which consequently reduces the ICT character (Marcus, 1993). For chlorinated solvents with intermediate polarity values, the PL quantum yield could also be affected by π*. The higher π* for DCM (0.82) compared to chloroform (0.58) facilitates a larger ICT character, increasing the fluorescence quantum yield up to 82%. A similar behavior was observed for the D–π–D–π–A architecture where the greater the stabilized ICT state, the higher the fluorescence quantum yield (Zimosz et al., 2022).

In the polar protic solvent, the H-bond ability of alcohols and thus the *α* value play a key role in the ICT character and, consequently, the emissive properties (Marcus, 1993). Both molecules displayed low fluorescence *Φ*_F_ in polar protic solvents such as ethanol (EtOH) and methanol (MeOH), as previously observed for related aminopyrimidines (Herbich et al., 1992; Herbich and Waluk, 1994). Hence, *Φ*_F_ is strongly influenced by the polarity and hydrogen-bonding ability of solvents used (Pannipara et al., 2014; Kalyagin et al., 2022). Furthermore, MeOH and EtOH present the values of 0.98 and 0.86, respectively, indicating a higher hydrogen-bond formation ability for MeOH, which reduces the donor character of the substituted-amine group and, consequently, decreases the fluorescence quantum yield (2%–3%) for both systems (Anderson et al., 2019). However, a quantum yield of 31% is observed in EtOH for **D2**, suggesting a less-effective interaction of EtOH with the electron pair of the amine nitrogen due to the steric hindrance introduced by the phenyl substituents compared with the methyl groups in **D1** (7%) (Kinoshita et al., 2000).

The significant redshift (35 nm) of the emission maximum in **D1** compared to **D2** in polar aprotic solvents such as DMSO could be rationalized due to a larger molecule planarization and better stabilization of the polar excited state by the solvent molecules of **D1**. Similar behavior has been observed in other systems such as *N*,*N*′-disubstituted dihydrodibenzo[a,c]phenazines (Chen et al., 2017).

**FIGURE 2:**
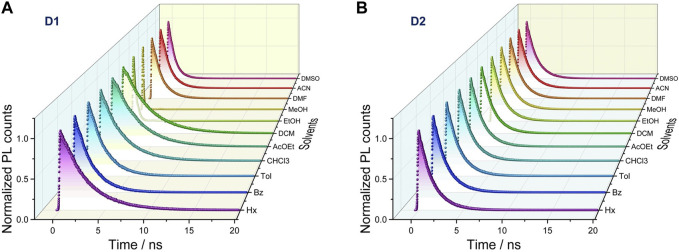
Time-resolved fluorescence spectra recorded in their maxima in different solvents under anaerobic conditions as a function of the dielectric constant for **D1 (A)** and **D2 (B)**. An excitation wavelength of 375 nm was employed using a pulsed laser (10 MHz).

Time-resolved fluorescence measurements were performed to analyze the dependence of the emissive properties of **D1** and **D2** on the dielectric constant of the solvent. The fluorescence decay profiles were recorded in all the aforementioned solvents (Figure 2 and Supplementary Table S1). Fluorescence decay traces showed a correlation of the lifetime parameter with solvent polarity only for **D2**, with lifetimes spanning from 1.51 ns to 4.64 ns from hexane to ACN. Nearly all the decays displayed a clear mono-exponential fitting, except for **D1** in DMF, ACN, and DMSO, which showed a biexponential behavior. It is worth noting that the fluorescence lifetime is drastically reduced in polar protic solvents for both compounds, and as a consequence, *Φ*_F_ decreases below 10% for **D1** (EtOH and MeOH) and to 31% and 3% for **D2** in EtOH and MeOH, respectively (Liu et al., 2022). Kinetic parameters (radiative and non-radiative rate constants) of both **D1** and **D2** systems were extracted from *Φ*_F_ and time-resolved measurements. As shown in Supplementary Table S2, the radiative rate constant (*k*_r_) decreased as the solvent polarity increased for **D1**, whereas the non-radiative rate constant (*k*_nr_) increased considerably, thus leading to a lower *Φ*_F_. In contrast, both the radiative and non-radiative pathways decreased upon increasing the solvent polarity for **D2** (Supplementary Table S2 and Supplementary Figure S1), thus resulting in a high *Φ*_F_ even in polar solvents. These data agree with the high dependence of the emissive properties on the nature of the *N*-donor substituent of the biphenylpyrimidine push–pull systems.

The electrochemical properties of **D1** and **D2** were then characterized through cyclic voltammetry (CV) measurements, and the energies of the highest occupied (HOMO) and lowest unoccupied molecular orbital (LUMO) were estimated using the energy level of ferrocene (Fc, 4.8 eV) as an external standard and calibrated by comparing with the *E*_1/2_(Fc/Fc^+^) half-wave electrode potential. The reduction potential of triarylpyrimidines has been reported to be dependent on the nature of the aryl groups. The introduction of electron-donor groups would increase the reduction potential to some extent (Itami et al., 2004). Supplementary Figure S2 shows the comparison of the cyclic voltametric responses of **D1** and **D2** in partially deaerated ACN solutions. The voltammograms showed an essentially reversible couple near −0.65 V vs. Ag/AgCl, corresponding to the well-known one-electron reduction of dissolved oxygen, accompanied by an apparently irreversible cathodic signal at approximately −2.1 and –2.1 V and an apparently irreversible anodic wave at approximately 1 and 0.9 V for **D1** and **D2**, respectively. The value recorded for the reduction potential is close to that previously reported and is attributed to the introduction of one electron into the pyrimidine ring, whereas the oxidation potential arises from the strong electron-donating ability of the dimethyl/phenylamino group (Itami et al., 2004; Qiao et al., 2020).

To estimate the electrochemical bandgaps of **D1** and **D2**, we employed the following widely used equations:

$$E_{\text{HOMO}}=-\;\left[E_{1/2}^{\text{ox}}\;–\;E_{1/2}\;\left(\frac{\text{Fc}}{{\text{Fc}}^{+}}\right)+4.8\right]\;\left(\text{eV}\right),$$ (3)

$$E_{\text{LUMO}}=-\;\left[E_{1/2}^{\text{red}}\;–\;E_{1/2}\;\left(\frac{\text{Fc}}{{\text{Fc}}^{+}}\right)+4.8\right]\;\left(\text{eV}\right),$$ (4)

where *E*_HOMO_ and *E*_LUMO_ represent the energies in the vacuum scale of HOMO and LUMO, respectively; *E*_ox_ and *E*_red_ are the half-peak electrode potentials corresponding to the oxidation and reduction of the tested compounds, respectively, and the *E*_1/2_(Fc/Fc^+^) half-wave electrode potential of the Fc/Fc^+^ couple is ca. 0.5 V under these conditions; all potentials relative to the reference electrode were used for voltametric measurements. Then, the electrochemical bandgap (∆*E* ^CV^) was calculated using Eq. (5):

$${∆E}^{\text{CV}}=E_{\text{LUMO}}–E_{\text{HOMO}}.$$ (5)

Since the oxidation and reduction of **D1** and **D2** are not electrochemically reversible processes, the half-wave potentials at 100 mV/s were used as approximate estimates of *E*_ox_ and *E*_red_. The corresponding values led to the bandgaps of 3.1 ± 0.1 and 3.0 ± 0.1 eV for **D1** and **D2**, respectively. These values are consistent with the respective optical bandgaps (∆*E* ^Opt^) determined for **D1** and **D2** (2.9 and 2.8 eV) derived from the absorption onset. The energy levels obtained by CV for **D1** and **D2** are very close to each other, with the HOMO level of **D1** slightly more stabilized than that of **D2**. Table 2 summarizes the electrochemical data.

**TABLE 2:** Electrochemical data, HOMO and LUMO energies, and energy gaps obtained by cyclic voltammetry and optical measurements.

Sample	*E*_ox_ (V) irreversible	*E*_*red*_ (V) irreversible	*E*_HOMO_ (eV)	*E*_LUMO_ (eV)	Δ*E* ^CV^ (eV)	Δ*E* ^Opt^ (eV)
**D1**	1.0	−2.1	−5.3	−2.2	3.1	2.9
**D2**	0.9	−2.1	−5.2	−2.2	3.0	2.8

According to thermal properties (Supplementary Figure S3), both **D1** and **D2** compounds present good thermal stability at the usual annealing temperature (>100°C) required for optical device preparation, as demonstrated by thermogravimetric (TGA) and differential scanning calorimetry (DSC) analyses. The higher decomposition temperature observed for **D2** agrees with the presence of the phenyl groups in the amine-donor moiety.

### Theoretical calculations

In order to explain the different optical behavior observed for **D1** and **D2**, the excited-state properties of these two molecules were theoretically investigated using DFT calculations for the ground state and TD-DFT calculations for the excited states. The geometry of the two systems was optimized, both in the ground electronic state (S_0_) and in the lowest singlet excited state (S_1_) at the DFT B3LYP/6-311G** and TD-DFT B3LYP/6-311G** levels of theory, respectively. Four solvents were chosen (hexane and toluene to describe the non-polar environments and ACN and DMSO to describe the polar solvents) to interpret the photophysical properties of **D1** and **D2**.

Supplementary Figure S4 shows the optimized geometries calculated for the electronic ground state (S_0_) of the two molecules in the four different solvents. The C_1_C_2_C_3_C_4_, C_5_C_6_C_7_C_8_, and C_9_C_10_N_1_C_11_ dihedral angles, which account for the internal twisting of the pyrimidine ring, the central biphenyl unit, and the amino group, respectively, and define the deviations from molecular planarity, are used for characterizing the obtained minima (Scheme 1 shows the atomic numbering). Independent of the solvent, the C_1_C_2_C_3_C_4_ and C_5_C_6_C_7_C_8_ angles present similar values for both **D1** and **D2** (approximately 20° and 35°, respectively), whereas the C_9_C_10_N_1_C_11_ angle shows significantly different values of approximately 8° and 35° for **D1** and **D2**, respectively. This difference is most probably the result of the steric hindrance introduced by the phenyl groups borne by the terminal amine group in **D2**. The conjugated core of molecules **D1** and **D2** therefore presents a maximum deviation of 35° from planarity.

**FIGURE 3:**
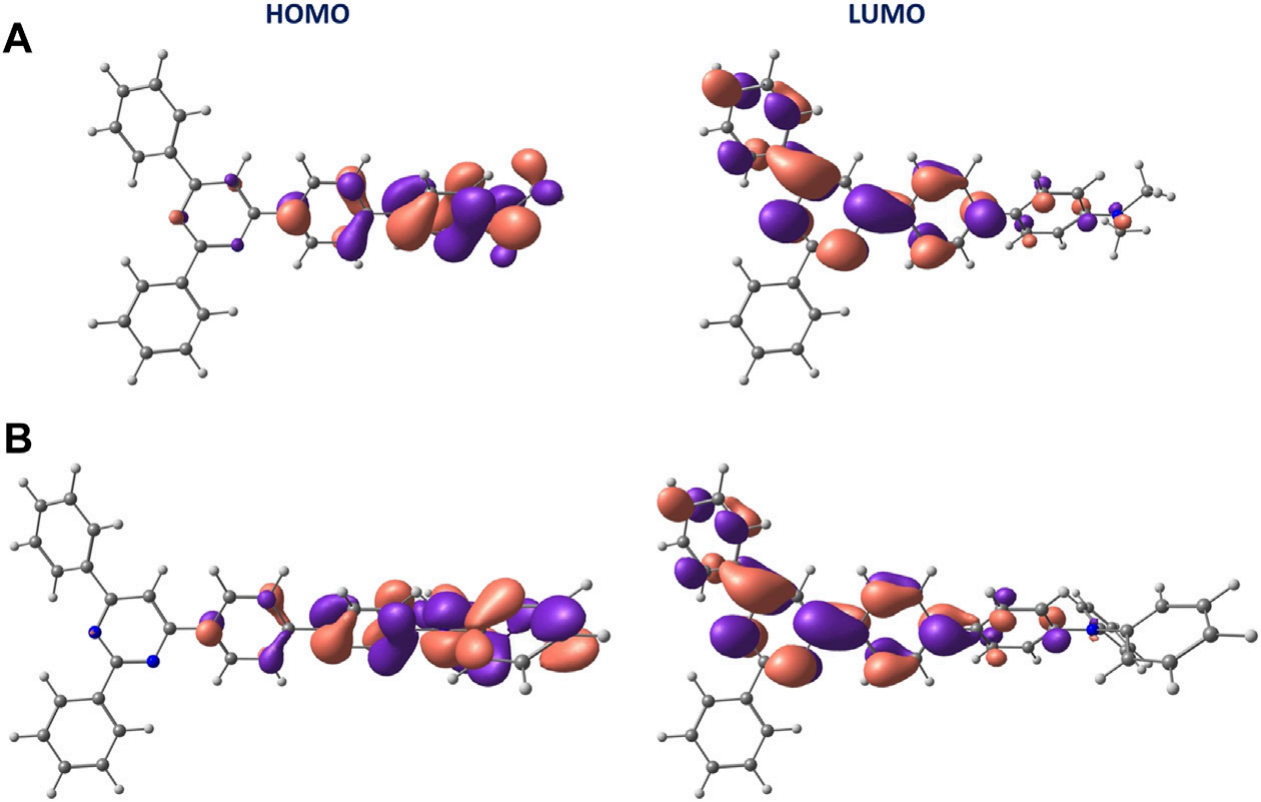
Isosurface contour plots (±0.03 a. u.) calculated at the B3LYP/6-311G**(PCM) level for the HOMO and LUMO orbitals in ACN of **D1 (A)** and **D2 (B)**.

To disentangle the absorption spectra, the electronic S_0_→S_n_ transitions were calculated at the S_0_ minimum-energy geometry using TD-DFT calculations. Independent of the solvent, the low- and high-energy bands experimentally observed at approximately 370 and 270 nm were assigned to transitions to the S_1_ and S_4_ singlet excited states, respectively. The nature of the S_1_ state for the four solvents considered in our calculations (hexane, toluene, ACN, and DMSO) is mainly of a charge-transfer character for both **D1** and **D2**. The state is mainly described by a one-electron excitation from HOMO to LUMO, which are respectively localized over the amine, with a large contribution of the biphenyl linker, and the pyrimidine part of the molecule (Figure 3 and Supplementary Figures S5, S6). The charge transfer upon excitation is supported by the localization of HOMO and LUMO orbitals over the four fragments defined in Scheme 1 that has been evaluated performing a Mulliken population analysis (Supplementary Table S3).

The charge-transfer nature of the S_1_ state is reflected in the value calculated for the dipole moment, equal to 36 and 32 D for **D1** and **D2**, respectively, which is considerably larger than that computed for the S_0_ state (6 and 3 D, respectively). The nature of the S_4_ state of **D1** for the four solvents considered is of a π–π* state localized on the pyrimidine part of the molecule, being described by a one-electron promotion from HOMO-1 to LUMO orbitals (Supplementary Figure S5 and Supplementary Table S4). In contrast, the S_4_ state of **D2** shows a charge-transfer character (similar to S_1_) and mainly results from the HOMO→LUMO+2 excitation (Supplementary Figure S6 and Supplementary Table S5). The nature of the S_1_ and S_4_ states of **D1** and **D2** is confirmed by computing the corresponding natural transition orbitals (NTOs), both at the ground-state minimum-energy geometry and at the S_1_ minima (Supplementary Figures S7, S8). The topology of NTOs closely resembles the topology characterizing the MOs involved in the respective electronic transition.

The absorption maxima of **D1**, computed as the vertical energy to the S_1_/S_4_ states at the optimized S_0_ minimum, are calculated at 447/315, 462/316, 483/316, and 492/316 nm (2.77/3.94, 2.68/3.92, 2.57/3.92, and 2.52/3.92 eV) in hexane, toluene, ACN, and DMSO, respectively. For **D2**, these values are 467/333, 480/336, 489/340, and 498/341 nm (2.66/3.72, 2.58/3.69, 2.54/3.65, and 2.49/3.64 eV), respectively. Then, theoretical calculations accurately predict that both absorptions weakly depend on the solvent polarity, being slightly redshifted passing from **D1** to **D2**. As previously suggested, this agrees with the small dipole moment calculated in S_0_ for **D2** (3 D) and the slightly larger value obtained for **D1** (6 D). Compared with the experimental results (Table 1), the theoretical data were redshifted for all the solvents considered. The reported redshifted trend in the theoretical energies is not surprising, considering the charge-transfer character of the excited states and the well-documented underestimation that B3LYP gives in such cases (Dreuw and Head-Gordon, 2004).

To gain insights into the changes in the fluorescence properties that **D1** and **D2** undergo with the solvent, the geometry of both molecules in the emitting S_1_ state was optimized at the TD-DFT 6-311G** level in the four solvents selected. The geometry optimization of S_1_ evolves differently for **D1** and **D2**. For **D1**, two minima were found for S_1_ (Supplementary Figure S9). In the first minimum, hereafter S_1,0_, the amine group is placed in the same plane of the adjacent phenyl ring, displaying a C_9_C_10_N_1_C_11_ dihedral angle at approximately 0°. In the second minimum, hereafter S_1,90_, the amine group is instead perpendicular to the plane of the adjacent phenyl ring, displaying a C_9_C_10_N_1_C_11_ dihedral angle close to 90°. Regarding the C_5_C_6_C_7_C_8_ twisting angle of the central biphenyl unit, its value decreases with respect to the S_0_ minimum by approximately 8° and 16° in the S_1,0_ and S_1,90_ minima, respectively, independent of the polarity of the solvent.

**FIGURE 4:**
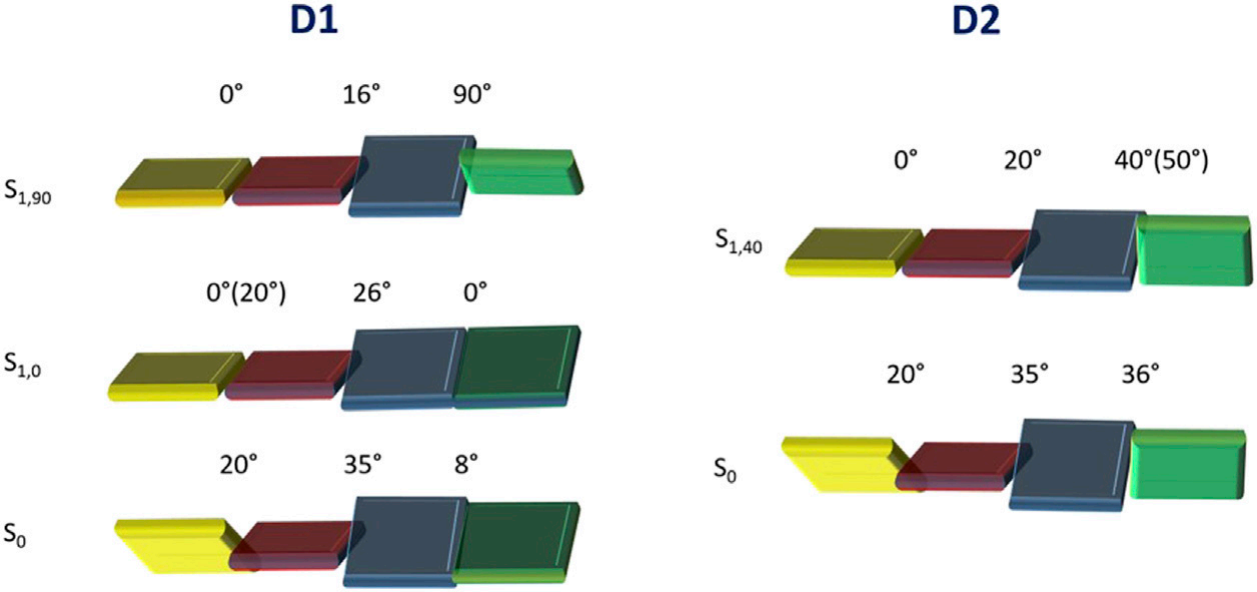
Schematic representation showing the twisting of the different molecular fragments constituting compounds **D1** and **D2**. Averaged values of the C_1_C_2_C_3_C_4_, C_5_C_6_C_7_C_8_, and C_9_C_10_N_1_C_11_ dihedral angles are given for the optimized S_0_ and S_1_ geometries of **D1** and **D2**. When the dihedral angle in non-polar solvents differs significantly, the value is reported in parenthesis. In order to better identify the dihedral angels, the same color code presented in Scheme 1 was adopted.

On the other hand, the C_1_C_2_C_3_C_4_ angle defining the twisting of the pyrimidine environment decreases to almost zero in all cases, except for the S_1,0_ minima in non-polar solvents, where it only decreases by a few degrees. The charge transfer between the amine and pyrimidine environments associated with the S_0_→S_1_ transition therefore determines that the conjugated skeleton of **D1** is, in general, more planar in S_1_ than in S_0_. For **D2**, only one minimum was obtained, hereafter S_1,40_, in which the amine group is significantly rotated (C_9_C_10_N_1_C_11_ dihedral angle equal to approximately 50° in hexane and toluene and at approximately 40° in ACN and DMSO), but it is not perpendicular to the phenyl ring. The non-planarity of such a structure is compatible with the steric hindrance offered by the phenyl rings in **D2**. All attempts to obtain a S_1,90_ structure for **D2** were unsuccessful. Regarding the C_1_C_2_C_3_C_4_ and C_5_C_6_C_7_C_8_ angles, the former decreases to almost zero, and the latter is reduced by approximately 15° under both polar and non-polar conditions. The variation in the three aforementioned dihedral angles passing from S_0_ to S_1_ is schematically summarized in Figure 4. The specific values of these dihedral angles shed light on the crucial role of the molecular geometry in their optical and electronic properties, including intramolecular charge transfer. According to the values obtained, the biphenyl spacer has a minimal impact on the connection with the acceptor triphenylpyrimidine moiety, thus facilitating ICT, but has a relevant impact on the conformation adopted by the amine-donor group passing from S_0_ to S_1_, depending on the substituent donor nature. For **D1**, a fully twisted 90° conformation is achieved upon excitation to the charge-transfer S_1_ state (S_1,90_), whereas only an intermediate twisting is possible for **D2** (S_1,40_).

From the characterized S_1_ minima, the vertical energy differences with respect to the ground state were computed and compared with the recorded fluorescence emission. For **D1**, the emission energies calculated from the S_1,0_ minima are equal to 541, 566, 729, and 725 nm (2.29, 2.19, 1.70, and 1.71 eV) in hexane, toluene, ACN, and DMSO, respectively. Despite the fact that these theoretical energies are considerably lower than the experimental energies, they correctly describe the experimentally recorded redshifting in the emission energy passing from non-polar to polar solvents. For example, the experimental data register a redshift of the emission in ACN with respect to toluene of approximately 0.6 eV, a value that agrees with the 0.5 eV difference based on the computational results. It is known that the functional used here tends to lower the energy to CT-type states (Cammi et al., 2000). Therefore, theoretical results predict the energetic minima of the CT character for all solvents, which are indeed in agreement with the experimental emission spectra in all solvent except hexane. Regarding the S_1,90_ minima of **D1**, they appear to be non-emissive, with the computed oscillator strength for the transition to the ground state being equal to zero. This is a consequence of the 90° rotation of the amine group, making the overlap between HOMO and LUMO insignificant (Supplementary Figure S10). For **D2** (Supplementary Figure S11), the emission energies computed from S_1,40_ are equal to 545, 561, 715, and 710 nm (2.27, 2.21, 1.73, and 1.75 eV) in hexane, toluene, ACN, and DMSO, respectively. Again, the absolute values predicted for the emission energies are too low compared to the experimental values but correctly described the redshifting of the emission from non-polar to polar solvents.

Employing the same example as that of **D1**, for **D2**, the experimental data found a redshift of 0.6 eV from toluene to ACN, a value that agrees with the 0.5 eV difference based on the computational results.

**FIGURE 5:**
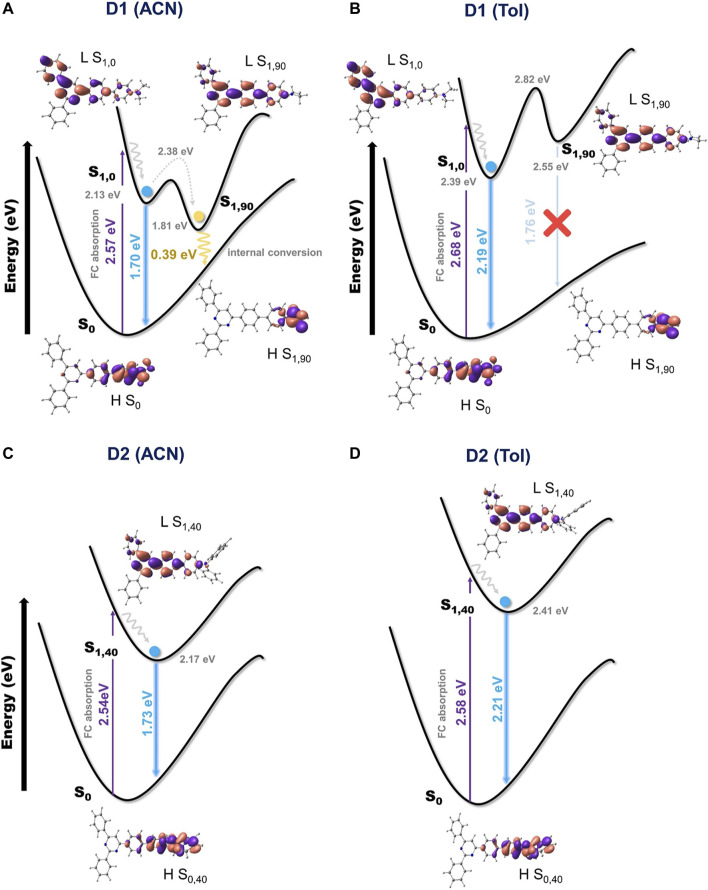
Schematic representation of the S_0_ and S_1_ PESs of **D1** **(A**, **B)** and **D2 (C, D)** in polar (ACN) and non-polar (toluene) solvents.

The drastic decrease observed experimentally for the value of *Φ*_F_ of **D1** in polar solvents can be rationalized by the presence of the S_1,90_ structure, which is instead absent in **D2**. The S_1,90_ minimum corresponds, in polar solvents, to the lowest S_1_ minimum-energy structure (i.e., lower than the S_1,0_ structure), and consequently, it will be the geometry toward which the S_1_ population will evolve (Supplementary Table S6 for **D1** and Supplementary Table S7 for **D2**). Considering the geometrical similarities between the S_0_ and S_1,0_ structures (Supplementary Figures S4, S9, S12), it is, however, plausible to assume that the molecule will initially decay to the S_1,0_ minimum. The energy barrier that separates the S_1,0_ and S_1,90_ structures was evaluated by computing the corresponding minimum energy path (MEP, Figure 5). From S_1,0_, an energy barrier of 0.25 and 0.24 eV was computed to reach S_1,90_ in ACN and DMSO, respectively. These relatively small values confirm the ability of the system to attain the S_1,90_ structure. Even more relevant is the fact that the S_1,90_ structure is separated from the ground state by a small energy gap of only 0.39 eV in polar solvents like ACN (0.40 eV in DMSO), which consequently favors the non-radiative decay back to the ground state (Figure 5A). The S_1,90_ structure is then able to promote the non-radiative decay of **D1** in polar solvents, thus explaining the experimentally recorded low *Φ*_F_ values for such environments. In non-polar solvents instead, S_1,0_ is the lowest S_1_ minimum (Supplementary Table S6) to which consequently the system will tend to evolve. The barrier from the S_1,0_ to S_1,90_ minima in non-polar solvents was computed to have values of 0.44 and 0.43 eV for hexane and toluene, respectively. The population of S_1,90_ in non-polar solvents is then much less probable than in polar solvents for two reasons: first, S_1,90_ is not the lowest S_1_ minimum; second, it requires to surmount a significantly higher energy barrier. Moreover, in non-polar solvents, the energy gap with the ground state at the S_1,90_ structure is of 1.94 and 1.76 eV in hexane and toluene, respectively, and will not allow a non-radiative decay (Figure 5B). This, together with the impossibility to emit fluorescence due to the small overlap between HOMO and LUMO orbitals, makes the contribution of the S_1,90_ structure to the photophysics of the **D1** molecule in non-polar environments of marginal relevance. These theoretical findings support the high *Φ*_F_ values experimentally observed for **D1** in non-polar solvents.

Finally, the absence of an S_1,90_ structure for **D2** explains why for such a system high *Φ*_F_ values are recorded in both non-polar and polar solvents (Figure 1; Table 1). The influence of the solvent polarity on the fluorescence properties of the **D2** molecule is thus reduced to a redshift of the emission passing from non-polar to polar solvents (Figures 5C,D).

## Conclusion

A large fluorosolvatochromism is observed for biphenylpyrimidine-based push–pull systems bearing dimethylamino (**D1**) or diphenylamino (**D2**) as donor groups, where the *N*,*N*-substituent (dimethyl or diphenyl) determines their emissive properties according to the polarity of the solvent. Theoretical calculations have demonstrated to be a key tool to explain this effect. In the case of **D1**, the presence of a twisted geometry for the lowest-energy, charge-transfer excited state (S_1,90_) was found to promote the non-radiative decay in polar solvents due to the lower energy of this structure and its energy proximity to the ground S_0_ state. In contrast, in non-polar solvents, the S_1,90_ structure is higher in energy and less attainable, making the non-radiative decay less likely. In the case of **D2**, the S_1,90_ structure is not a minimum due to the steric hindrance between the phenyl rings of the amine group, and consequently, the fluorescence quantum yield is maintained independently of the polarity of the solvent. These systems are of high interest as possible hole transporters in electroluminescent devices based on semiconductor materials such as perovskites, due to their stunning emissive and electronic properties.

## Data Availability

The original contributions presented in the study are included in the article/Supplementary Material; further inquiries can be directed to the corresponding authors.
