# Supplementary material for: Governing the emissive properties of 4-aminobiphenyl-2-pyrimidine push–pull systems via the restricted torsion of N,N-disubstituted amino groups
Source: Front Chem. 2023 Nov 10;11:1292541. doi: 10.3389/fchem.2023.1292541 (PMC10667708; doi:10.3389/fchem.2023.1292541)
Supplement: Supplementary file 1 [file DataSheet1.docx]

Supplementary Material

**Governing the emissive properties of 4-aminobiphenyl-2-pyrimidine push-pull systems via restricted torsion of the *N*,*N*-disubstituted amino groups**

Alejandro Cortés-Villena,^1^ Iván Soriano-Díaz,^1^ Moisés Domínguez,^2^ Matías Vidal,^2^ Pablo Rojas,^2^ Carolina Aliaga,^2,3*^ Angelo Giussani,^1^ Antonio Doménech-Carbó,^4^ Enrique Ortí,^1*^ Raquel E. Galian,^1*^ Julia Pérez-Prieto^1*^

^1^Instituto de Ciencia Molecular, Universidad de Valencia, Catedrático José Beltrán Martínez 2, 46980, Paterna (Valencia), España

^2^Facultad de Química y Biología, Universidad de Santiago de Chile, Av. Libertador Bernardo O'Higgins 3363, Santiago, Chile

^3^Centro para el Desarrollo de la Nanociencia y la Nanotecnología, CEDENNA, Universidad de Santiago de Chile, Av. Libertador Bernardo O'Higgins 3363, Santiago, Chile

^4^Departamento de Química Analítica, Universidad de Valencia, Dr. Moliner 50, 46100, Burjassot (Valencia), España

| **Table of contents** |  |
| --- | --- |
| **Table S1.** Fluorescence kinetic parameters. | **S3-S5** |
| **Table S2.** Radiative/non-radiative rate constants. | **S5-S7** |
| **Figure S1.** Comparison of the radiative/non-radiative rate constants. | **S7** |
| **Figure S2.** Cyclic voltammograms. | **S8** |
| **Figure S3.** Thermal properties. | **S8** |
| **Figure S4.** DFT-optimized ground-state (S_0_) geometries. | **S9** |
| **Figure S5.** Molecular orbitals of **D1** at the S_0_ geometry. | **S10** |
| **Figure S6.** Molecular orbitals of **D2** at the S_0_ geometry. | **S11** |
| **Table S3.** Main atomic contribution to the HOMO and LUMO. | **S12** |
| **Table S4.** Vertical energies (eV), oscillator strength, dipole moment (D) and main monoexcitations for the S_1_, S_2_, S_3_, S_4_ states at the S_0_ minimum for the **D1** molecule. | **S13-S14** |
| **Table S5.** Vertical energies (eV), oscillator strength, dipole moment (D) and main monoexcitations for the S_1_, S_2_, S_3_, S_4_ states at the S_0_ minimum for the **D2** molecule. | **S14-S15** |
| **Figure S7.** Natural transition orbital (NTO) pairs at the S_0_ minimum-energy geometry for **D1**. | **S16** |
| **Figure S8.** Natural transition orbital (NTO) pairs at the S_1,0_ and S_1,90_ excited-state minimum-energy geometries for **D1**. | **S17** |
| **Figure S9.** TD-DFT-optimized geometries of the S_1_ excited state. | **S18-S19** |
| **Figure S10.** Molecular orbitals of **D1** at the S_1_ excited state geometry. | **S20** |
| **Figure S11.** Molecular orbitals of **D2** at the S_1_ excited state geometry. | **S21** |
| **Figure S12.** Optimized bond distances for the S_0_ and S_1_ states of **D1** and **D2**. | **S22** |
| **Table S6.** Relative energies for the S_0_ and S_1_ states of **D1**. | **S23** |
| **Table S7.** Relative energies for the S_0_ and S_1_ states of **D2**. | **S24** |

Table S1. Fluorescence kinetic parameters of D1 and D2 in different solvents of decreasing dielectric constant under anaerobic conditions in TCSPC mode.

| **Compound** | **Solvent, dielectric constant (*ε*)** | ***τ*_1_ ± sd**  **(ns)** | ***A*_1_**  **(%)** | ***τ*_2_ ± sd**  **(ns)** | ***A*_2_**  **(%)** | ***τ*_av_ ± sd**  **(ns)** |
| --- | --- | --- | --- | --- | --- | --- |
| **D1** | Dimethylsulfoxide (DMSO, *ε* = 46.7) | 0.83 ± 0.003 | 86 | 1.88 ± 0.021 | 14 | 0.98 ± 0.007 |
|  | Acetonitrile  (ACN, *ε* = 37.5) | 1.13 ± 0.006 | 78 | 1.95 ± 0.027 | 22 | 1.31 ± 0.013 |
|  | Dimethylformamide (DMF, *ε* = 36.7) | 0.87 ± 0.013 | 31 | 1.65 ± 0.007 | 69 | 1.4 ± 0.007 |
|  | Methanol  (MeOH, *ε* = 32.7) | - | - | - | - | - |
|  | Ethanol  (EtOH, *ε* = 24.5) | - | - | - | - | - |
|  | Dichloromethane  (DCM, *ε* = 8.9) | 3.36 ± 0.002 | 100 | - | - | 3.36 ± 0.002 |
|  | Ethyl acetate  (AcOEt, *ε* = 6.0) | 3.13 ± 0.002 | 100 | - | - | 3.13 ± 0.002 |
|  | Chloroform  (CHCl_3_, *ε* = 4.8) | 2.66 ± 0.002 | 100 | - | - | 2.66 ± 0.002 |
|  | Toluene  (Tol, *ε* = 2.4) | 1.86 ± 0.001 | 100 | - | - | 1.86 ± 0.001 |
|  | Benzene  (Bz, *ε* = 2.3) | 1.87 ± 0.002 | 100 | - | - | 1.87 ± 0.002 |
|  | Hexane  (Hx, *ε* = 1.9) | 1.43 ± 0.001 | 100 | - | - | 1.43 ± 0.001 |
| **D2** | Dimethylsulfoxide (DMSO, *ε* = 46.7) | 4.25 ± 0.002 | 100 | - | - | 4.25 ± 0.002 |
|  | Acetonitrile  (ACN, *ε* = 37.5) | 4.64 ± 0.002 | 100 | - | - | 4.64 ± 0.002 |
|  | Dimethylformamide (DMF, *ε* = 36.7) | 4.29 ± 0.002 | 100 | - | - | 4.29 ± 0.002 |
|  | Methanol  (MeOH, *ε* = 32.7) | - | - | - | - | - |
|  | Ethanol  (EtOH, *ε* = 24.5) | 1.72 ± 0.001 | 100 | - | - | 1.72 ± 0.001 |
|  | Dichloromethane  (DCM, *ε* = 8.9) | 3.47 ± 0.001 | 100 | - | - | 3.47 ± 0.001 |
|  | Ethyl acetate  (AcOEt, *ε* = 6.0) | 2.99 ± 0.001 | 100 | - | - | 2.99 ± 0.001 |
|  | Chloroform  (CHCl_3_, *ε* = 4.8) | 2.76 ± 0.001 | 100 | - | - | 2.76 ± 0.001 |
|  | Toluene  (Tol, *ε* = 2.4) | 1.85 ± 0.049 | 100 | - | - | 1.85 ± 0.049 |
|  | Benzene  (Bz, *ε* = 2.3) | 1.84 ± 0.001 | 100 | - | - | 1.84 ± 0.001 |
|  | Hexane  (Hx, *ε* = 1.9) | 1.51 ± 0.001 | 100 | - | - | 1.51 ± 0.001 |

*τ*_1_, *τ*_2_ and *τ*_av_ are the 1 and 2 lifetime components and the average lifetime, respectively. *A*_1_ and *A*_2_ are the respective contributions of each lifetime component. A 375 nm pulsed laser (10 MHz) was used as an excitation source. Decays were fitted to a single- or biexponential decay function of the following form: $I\left( t \right)= I\left( 0 \right)\sum_{i} {A_{i}\cdot e}^{\frac{-t}{\tau_{i}}}$ taking into consideration the IRF (reconvolution analysis). The average lifetime (*τ*_av_) parameter was calculated by using the following expression: $\tau_{av}= \frac{\sum B_{i}\cdot\tau_{i}^{2}}{\sum B_{i}\cdot\tau_{i}}$. The relative contribution ($A_{i}$) was calculated through: $A_{i}(\%)= \frac{B_{1}\cdot\tau_{1}}{\sum B_{i}\cdot\tau_{i}}\cdot100$.

**Table S2.** Radiative and non-radiative rate constants of **D1** and **D2** in different solvents of decreasing dielectric constant determined in anaerobic conditions.

| **Compound** | **Solvent, dielectric constant (*ε*)** | ***τ*_av_ ± sd**  **(ns)** | ***Φ*_F_ ± sd** | ***k*_r_ ± sd**  **× 10^8^ (s^–1^)** | ***k*_nr_ ± sd**  **× 10^8^ (s^–1^)** |
| --- | --- | --- | --- | --- | --- |
| **D1** | Dimethylsulfoxide (DMSO, *ε* = 46.7) | 0.83 ± 0.003 | 0.16 ± 0.1 | 1.63 ± 0.066 | 8.57 ± 0.069 |
|  | Acetonitrile  (ACN, *ε* = 37.5) | 1.13 ± 0.006 | 0.19 ± 0.1 | 1.45 ± 0.058 | 6.18 ± 0.054 |
|  | Dimethylformamide (DMF, *ε* = 36.7) | 0.87 ± 0.013 | 0.29 ± 0.1 | 2.07 ± 0.049 | 5.07 ± 0.062 |
|  | Methanol  (MeOH, *ε* = 32.7) | - | 0.02 ± 0.1 | - | - |
|  | Ethanol  (EtOH, *ε* = 24.5) | - | 0.07 ± 0.1 | - | - |
|  | Dichloromethane  (DCM, *ε* = 8.9) | 3.36 ± 0.002 | 0.82 ± 0.1 | 2.44 ± 0.013 | 0.54 ± 0.015 |
|  | Ethyl acetate  (AcOEt, *ε* = 6.0) | 3.13 ± 0.002 | 0.54 ± 0.1 | 1.73 ± 0.019 | 1.47 ± 0.021 |
|  | Chloroform  (CHCl_3_, *ε* = 4.8) | 2.66 ± 0.002 | 0.41 ± 0.1 | 1.54 ± 0.025 | 2.22 ± 0.027 |
|  | Toluene  (Tol, *ε* = 2.4) | 1.86 ± 0.001 | 0.87 ± 0.1 | 4.68 ± 0.012 | 0.7 ± 0.013 |
|  | Benzene  (Bz, *ε* = 2.3) | 1.87 ± 0.002 | 0.96 ± 0.1 | 5.13 ± 0.011 | 0.21 ± 0.013 |
|  | Hexane  (Hx, *ε* = 1.9) | 1.43 ± 0.001 | 0.87 ± 0.1 | 6.08 ± 0.012 | 0.91 ± 0.013 |
| **D2** | Dimethylsulfoxide (DMSO, *ε* = 46.7) | 4.25 ± 0.002 | 0.79 ± 0.1 | 1.86 ± 0.013 | 0.49 ± 0.015 |
|  | Acetonitrile  (ACN, *ε* = 37.5) | 4.64 ± 0.002 | 0.78 ± 0.1 | 1.68 ± 0.013 | 0.47 ± 0.015 |
|  | Dimethylformamide (DMF, *ε* = 36.7) | 4.29 ± 0.002 | 0.87 ± 0.1 | 2.03 ± 0.012 | 0.3 ± 0.014 |
|  | Methanol  (MeOH, *ε* = 32.7) | - | 0.03 ± 0.1 | - | - |
|  | Ethanol  (EtOH, *ε* = 24.5) | 1.72 ± 0.001 | 0.31 ± 0.1 | 1.8 ± 0.033 | 4.01 ± 0.034 |
|  | Dichloromethane  (DCM, *ε* = 8.9) | 3.47 ± 0.001 | 0.91 ± 0.1 | 2.62 ± 0.011 | 0.26 ± 0.012 |
|  | Ethyl acetate  (AcOEt, *ε* = 6.0) | 2.99 ± 0.001 | 0.82 ± 0.1 | 2.74 ± 0.013 | 0.6 ± 0.014 |
|  | Chloroform  (CHCl_3_, *ε* = 4.8) | 2.76 ± 0.001 | 0.84 ± 0.1 | 3.04 ± 0.012 | 0.58 ± 0.013 |
|  | Toluene  (Tol, *ε* = 2.4) | 1.85 ± 0.049 | 0.96 ± 0.1 | 5.19 ± 0.037 | 0.22 ± 0.086 |
|  | Benzene  (Bz, *ε* = 2.3) | 1.84 ± 0.001 | 0.69 ± 0.1 | 3.75 ± 0.015 | 1.68 ± 0.016 |
|  | Hexane  (Hx, *ε* = 1.9) | 1.51 ± 0.001 | 0.75 ± 0.1 | 4.97 ± 0.014 | 1.66 ± 0.015 |

*Φ*_F_ is the fluorescence quantum yield, *k*_r_ and *k*_nr_ are the radiative and non-radiative rate constants calculated as $k_{r} = \frac{\Phi_{Fl}}{\tau_{av}}$ and $k_{nr} = {\tau_{av}}^{-1}-k_{r}$, respectively.





Figure S1. Comparison of the kinetic constants (*k*_r_: radiative constant and *k*_nr_: non-radiative constant) of D1 and D2 in different solvents of increasing dielectric constant.





Figure S2. Cyclic voltammograms of 1 mM solutions of D1 (green curve) and D2 (purple curve) in partially de-aerated 0.10 M TBABF_4_/ACN at a potential scan rate of 100 mV/s.


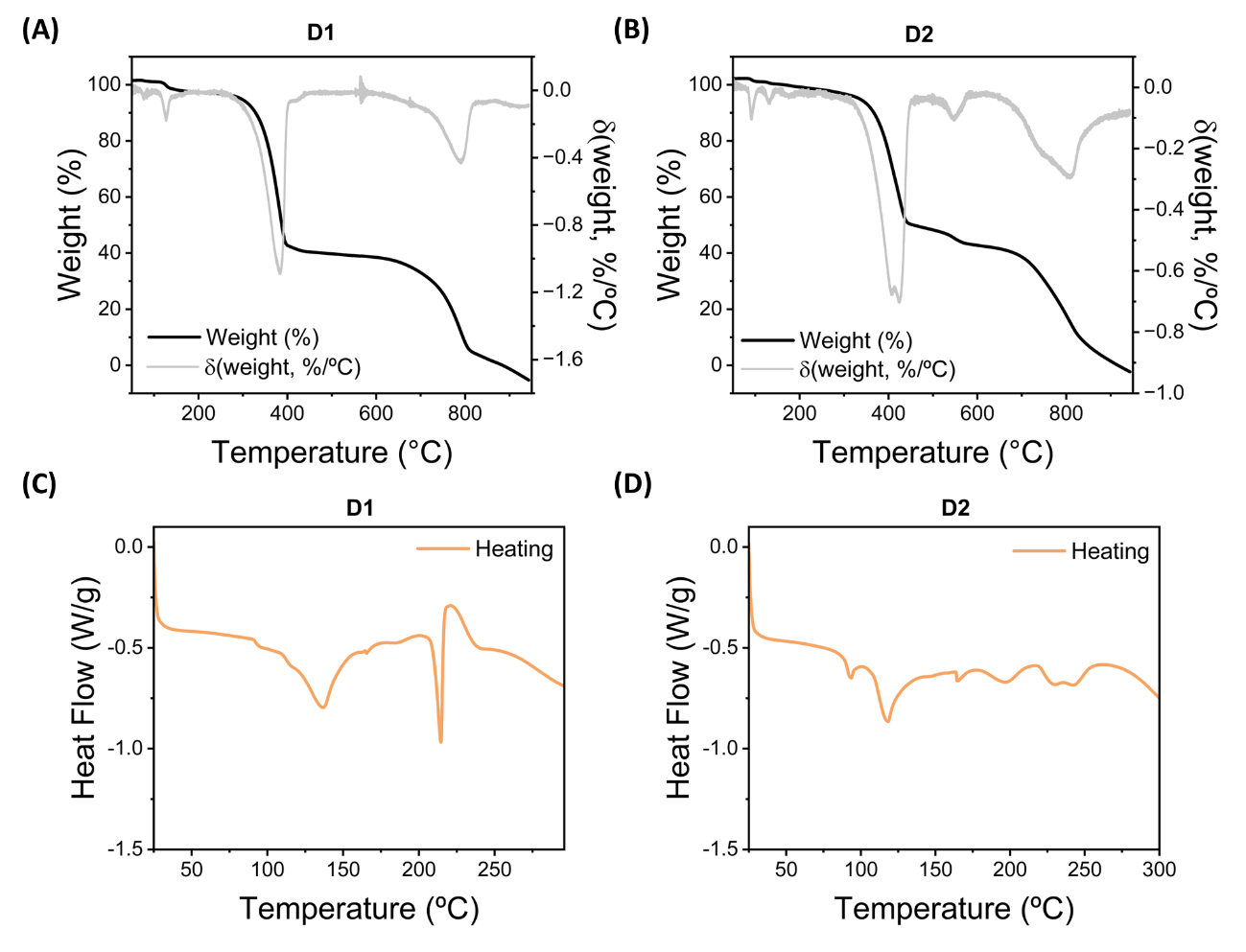


Figure S3. TGA analysis of D1 (A) and D2 (B) compounds. DSC analysis of D1 (C) and D2 (D) compounds.


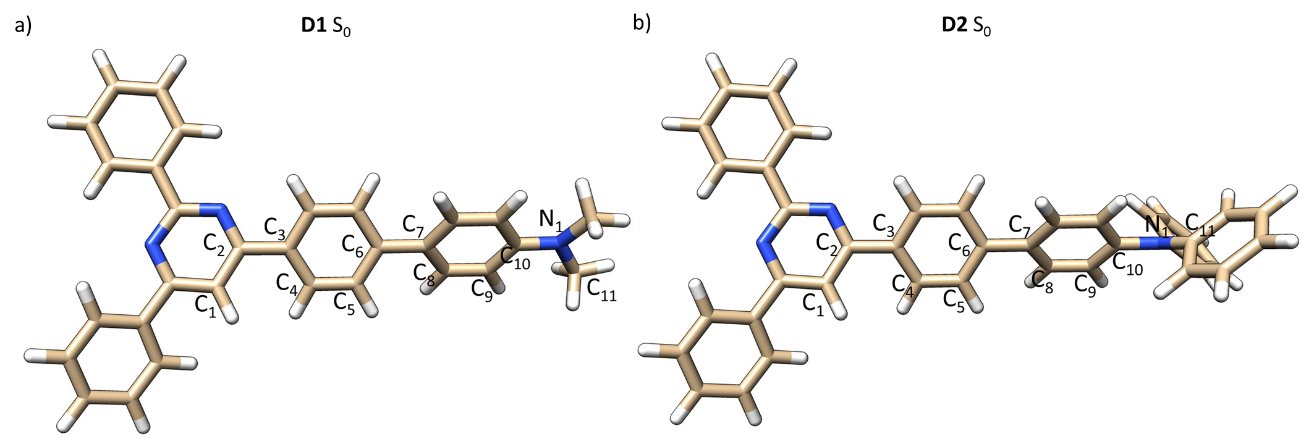


| **Solvent** | **Dihedral** | **D1** | **D2** |
| --- | --- | --- | --- |
| **Hx** | C_1_C_2_C_3_C_4_ | 21.35 | 21.81 |
|  | C_5_C_6_C_7_C_8_ | 34.35 | 35.65 |
|  | C_9_C_10_N_1_C_11_ | 8.59 | 37.59 |
| **Tol** | C_1_C_2_C_3_C_4_ | 21.17 | 21.91 |
|  | C_5_C_6_C_7_C_8_ | 33.93 | 35.39 |
|  | C_9_C_10_N_1_C_11_ | 8.45 | 37.44 |
| **ACN** | C_1_C_2_C_3_C_4_ | 20.81 | 22.60 |
|  | C_5_C_6_C_7_C_8_ | 31.37 | 34.16 |
|  | C_9_C_10_N_1_C_11_ | -8.46 | 36.65 |
| **DMSO** | C_1_C_2_C_3_C_4_ | 20.80 | 22.51 |
|  | C_5_C_6_C_7_C_8_ | 31.34 | 34.12 |
|  | C_9_C_10_N_1_C_11_ | -8.46 | 36.59 |

**Figure S4.** DFT/B3LYP/6-311G**-optimized geometries calculated for the ground state (S_0_) of **D1** (a) and **D2** (b). The values of the dihedral angles defining the optimized structure are given in the table expressed in degrees. Solvents effects (Hx, Tol, ACN and DMSO) were considered using the PCM method.

| 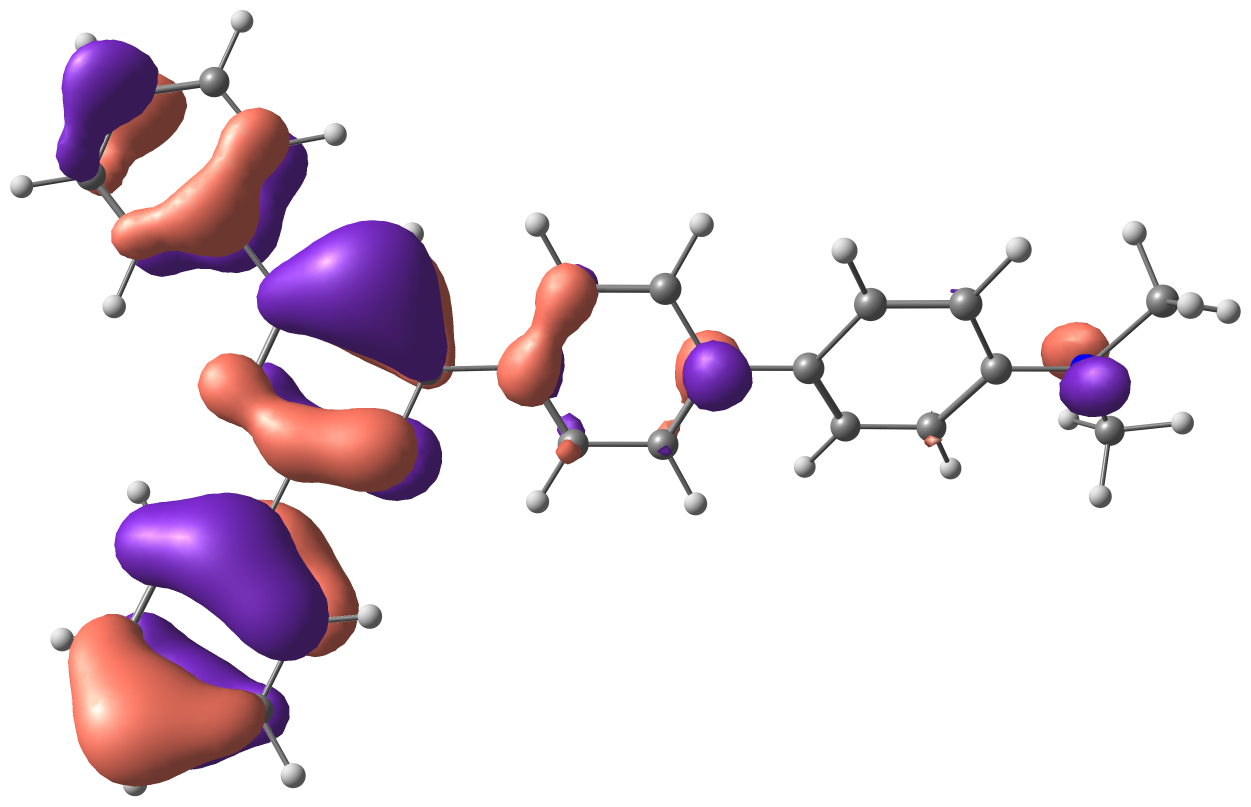  H – 1(Hx) | 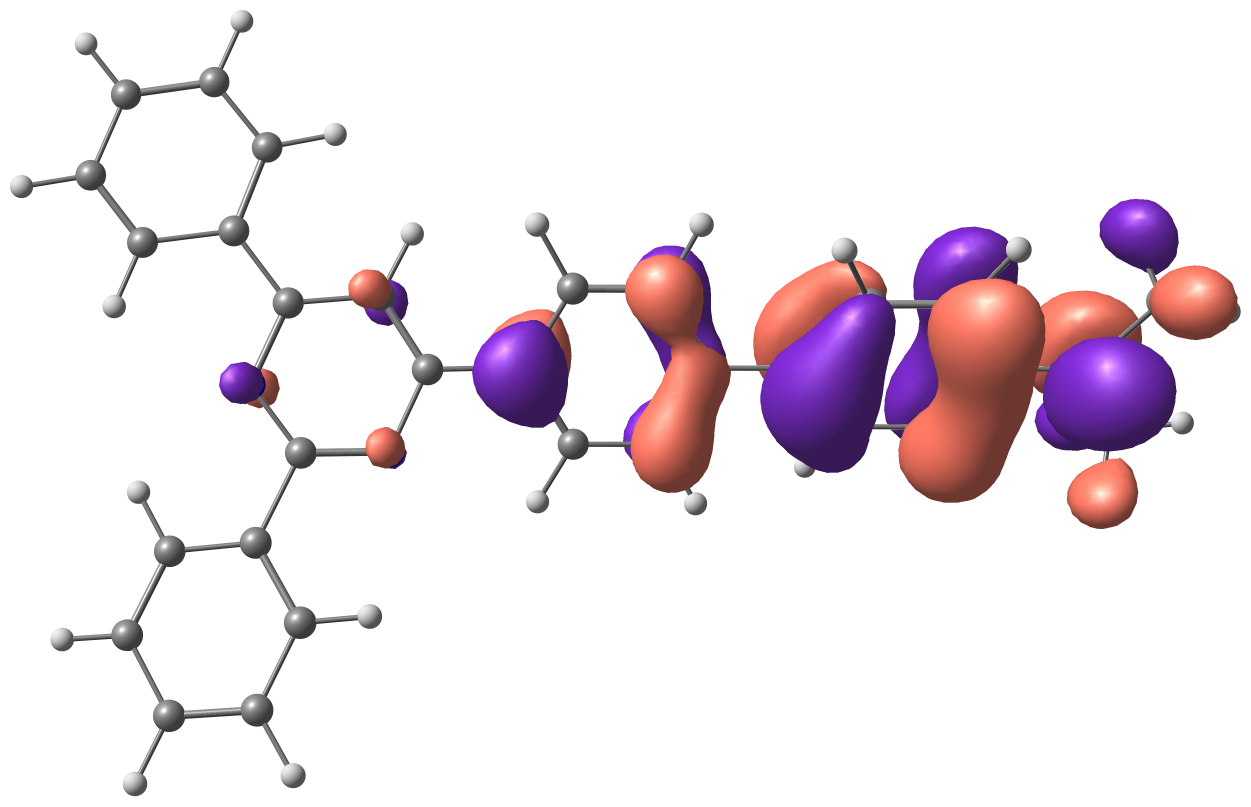  HOMO (Hx) | 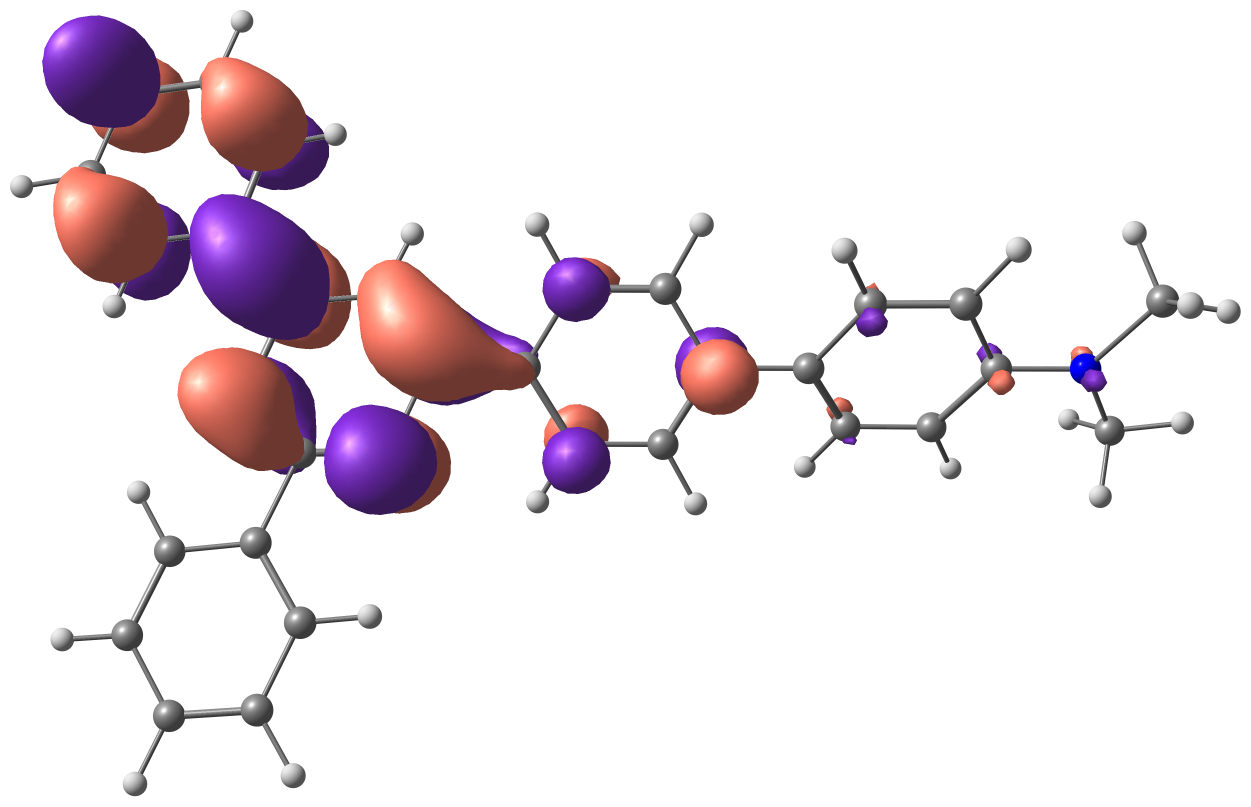  LUMO (Hx) | 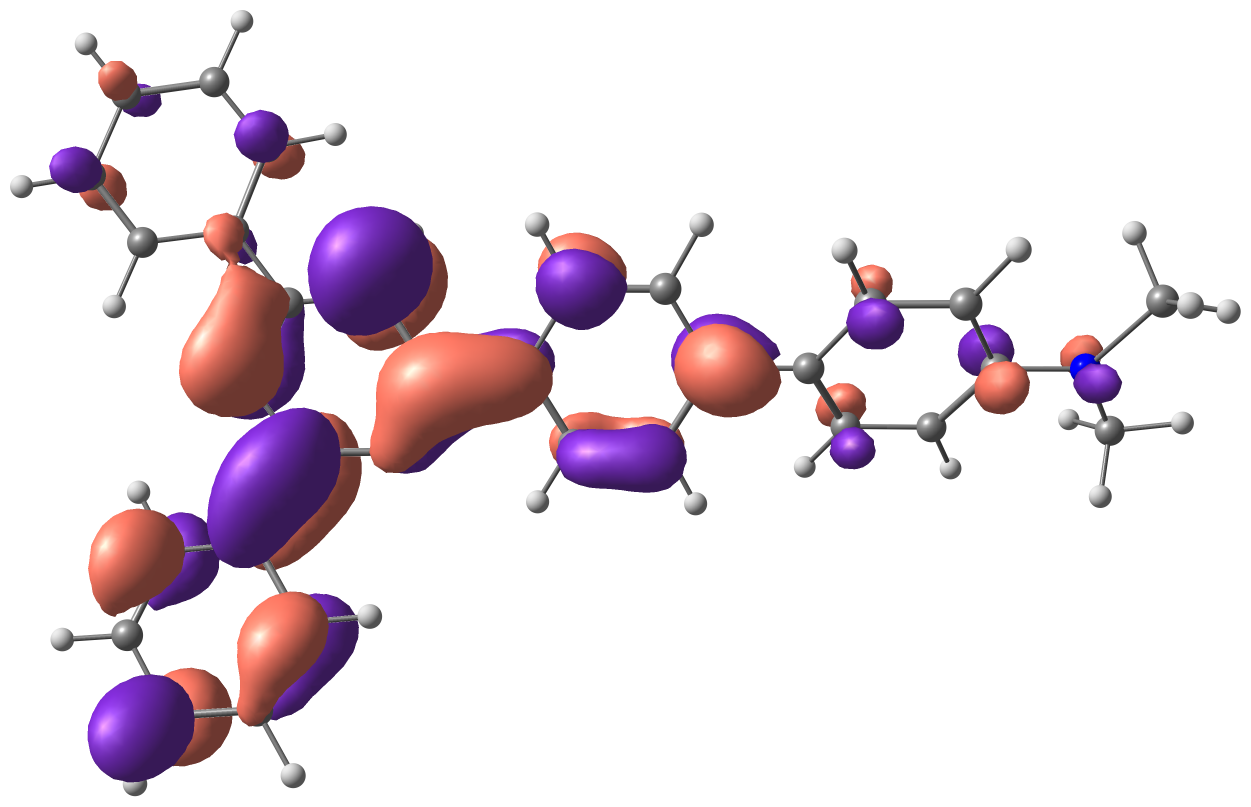  L + 1 (Hx) |
| --- | --- | --- | --- |
| 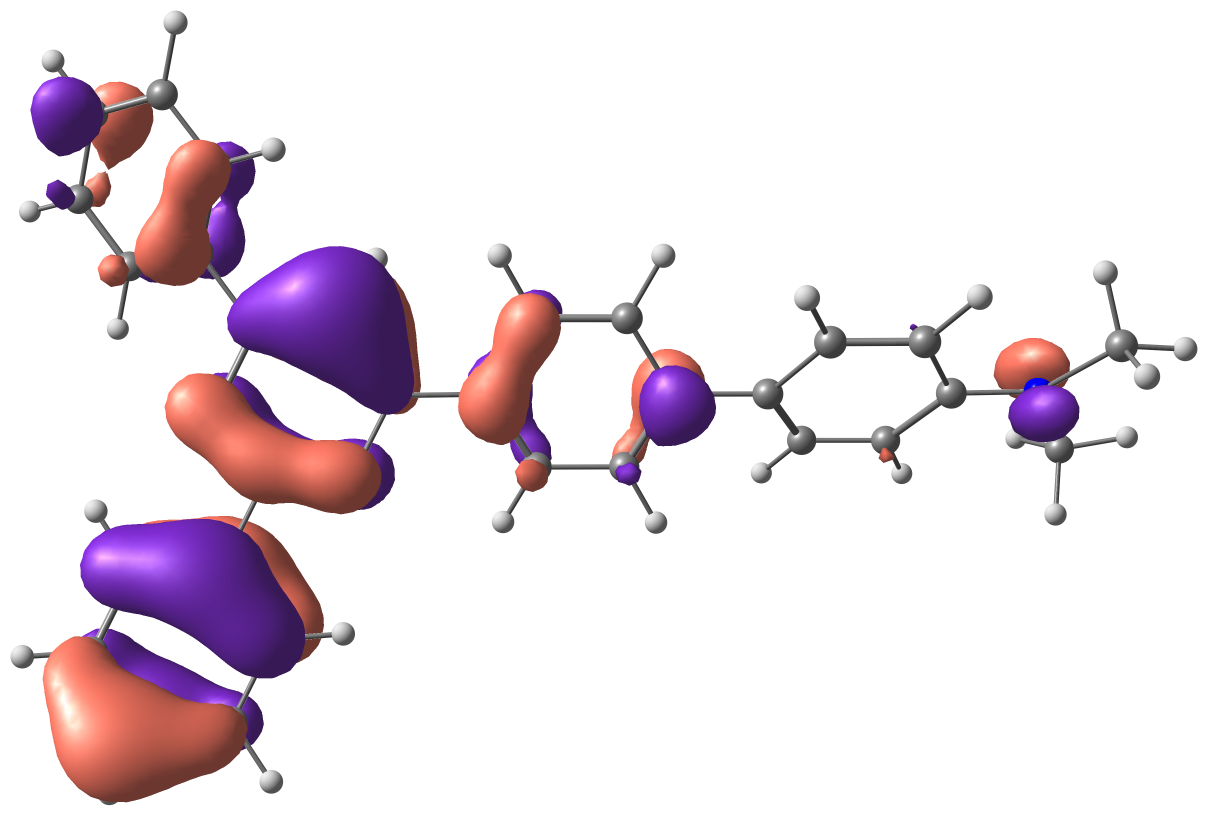  H – 1(Tol) | 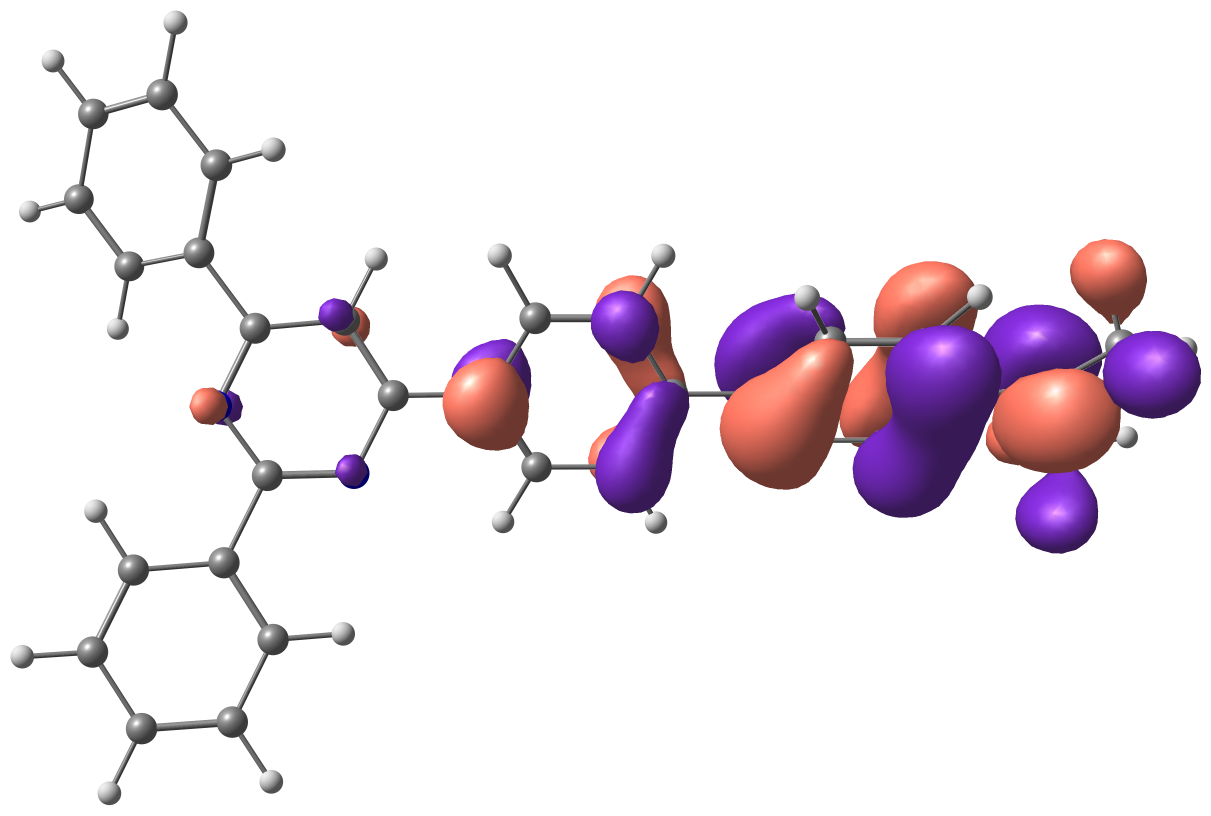  HOMO (Tol) | 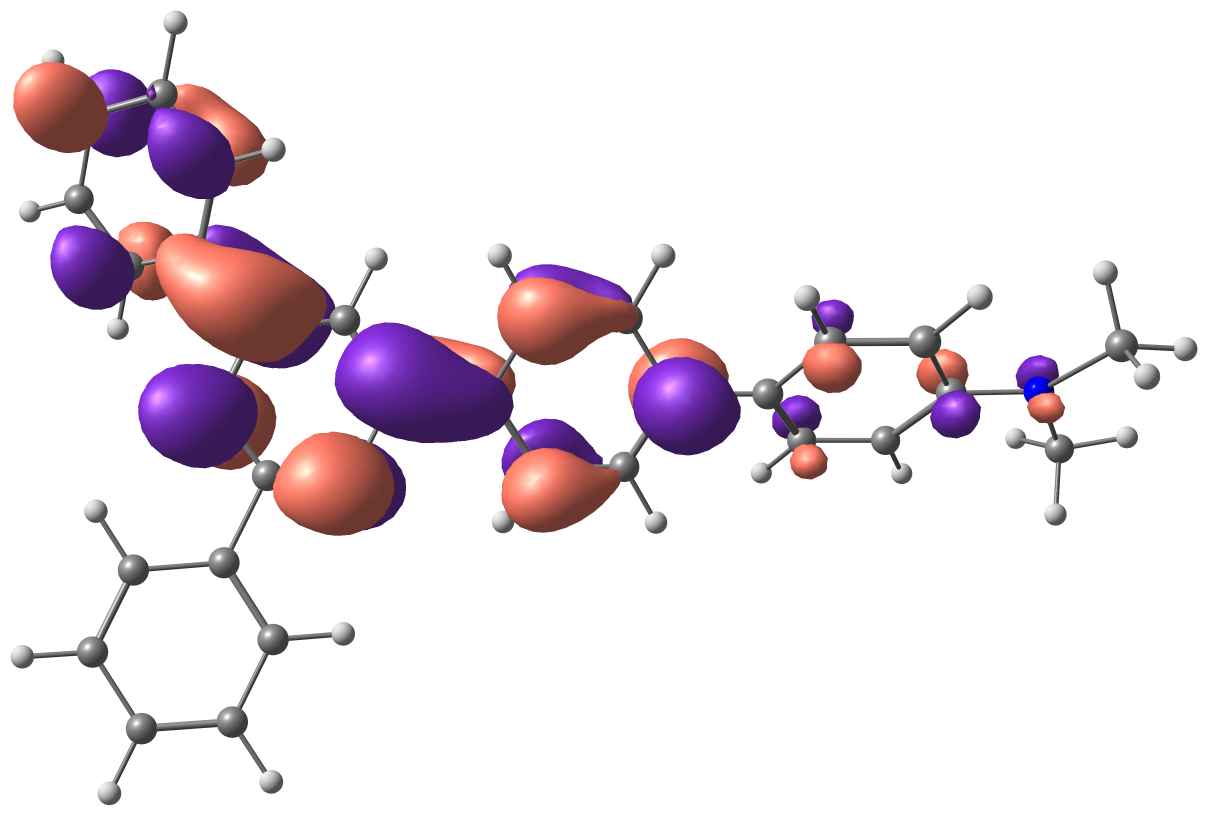  LUMO (Tol) | 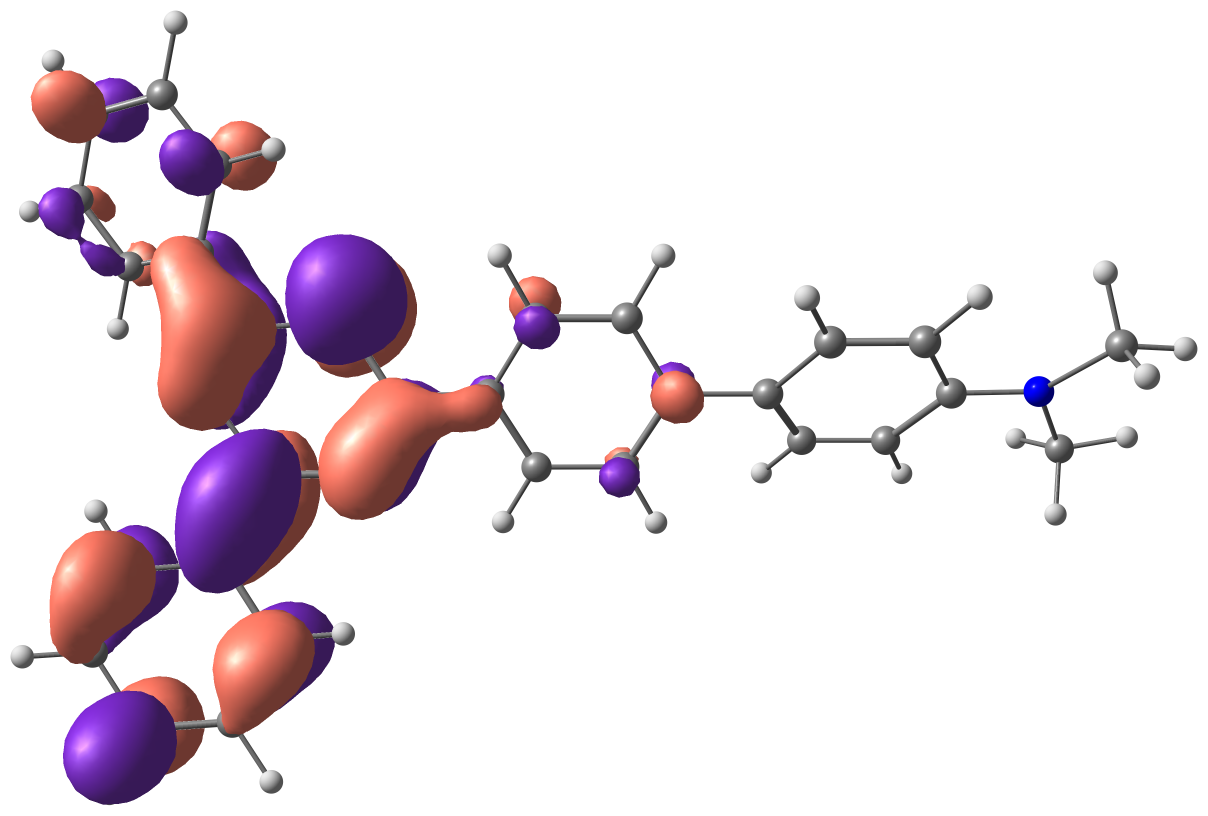  L + 1 (Tol) |
| 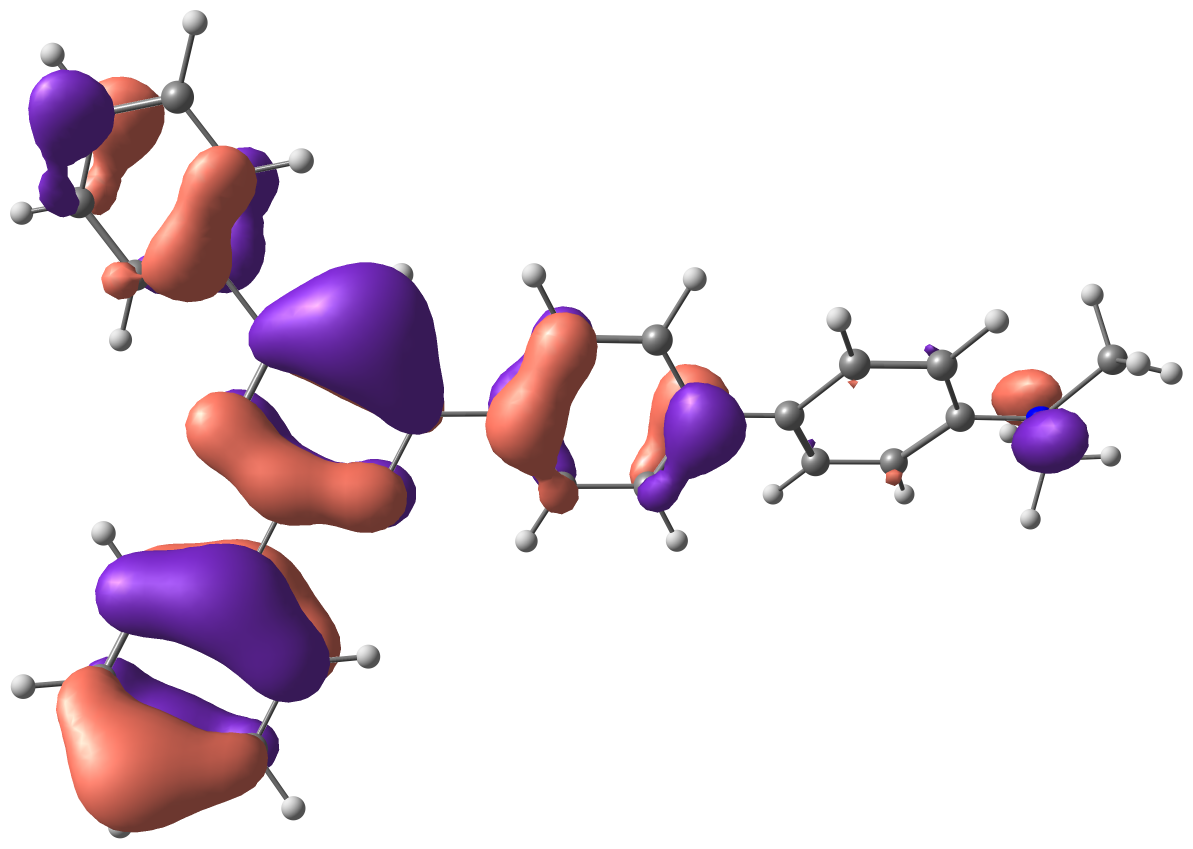  H – 1 (ACN) | 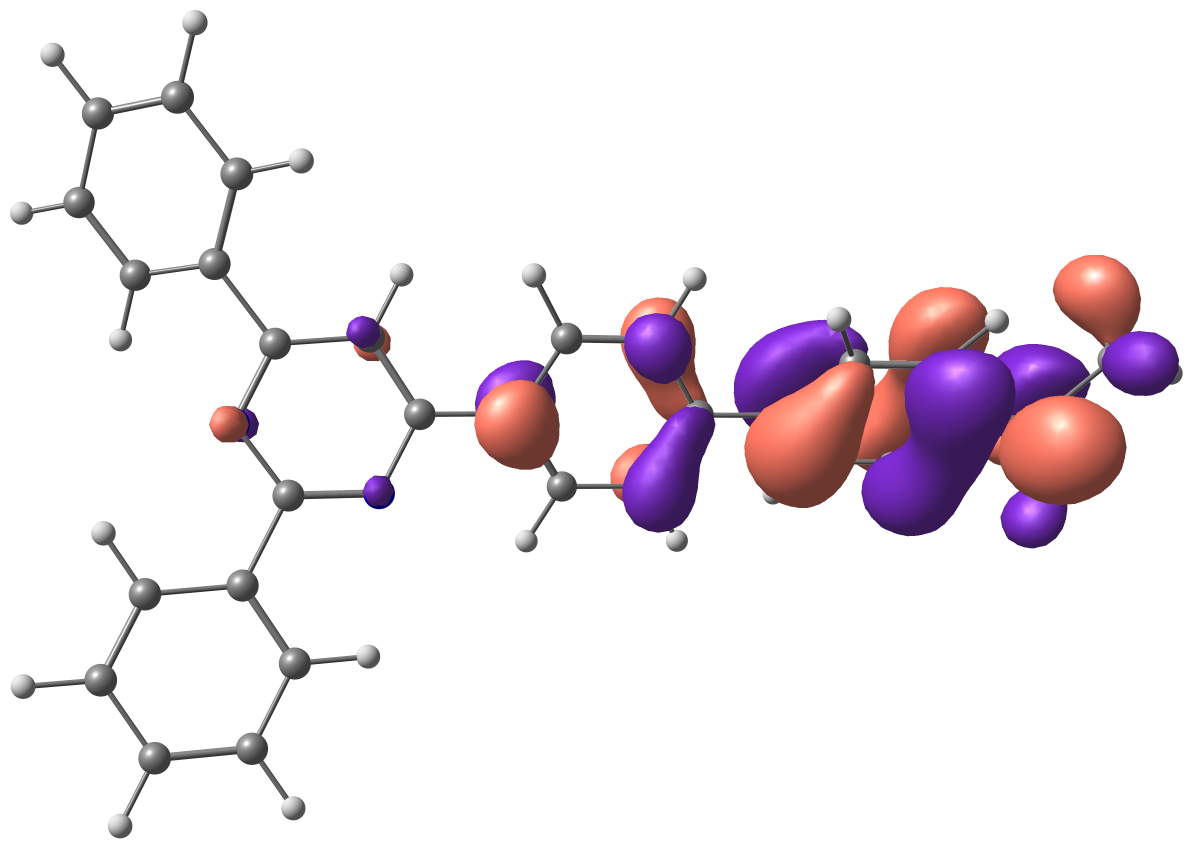  HOMO (ACN) | 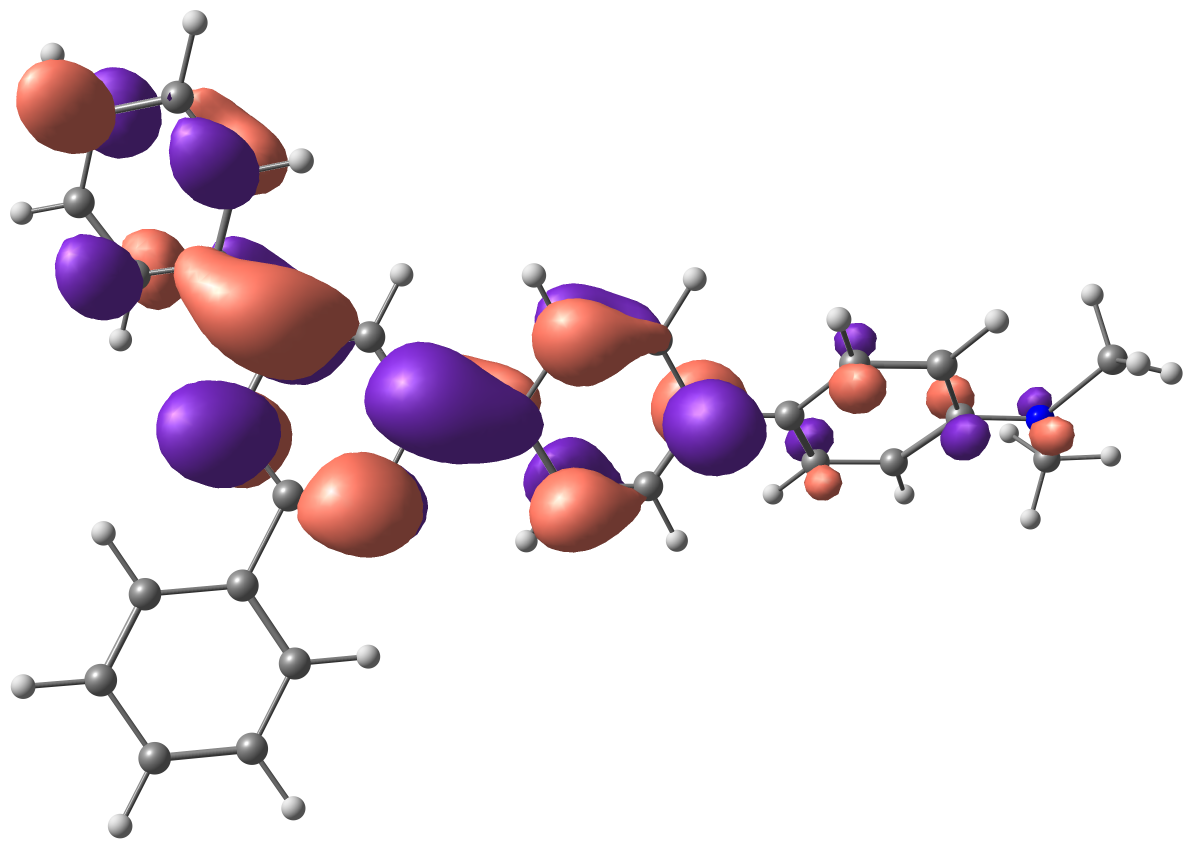  LUMO (ACN) | 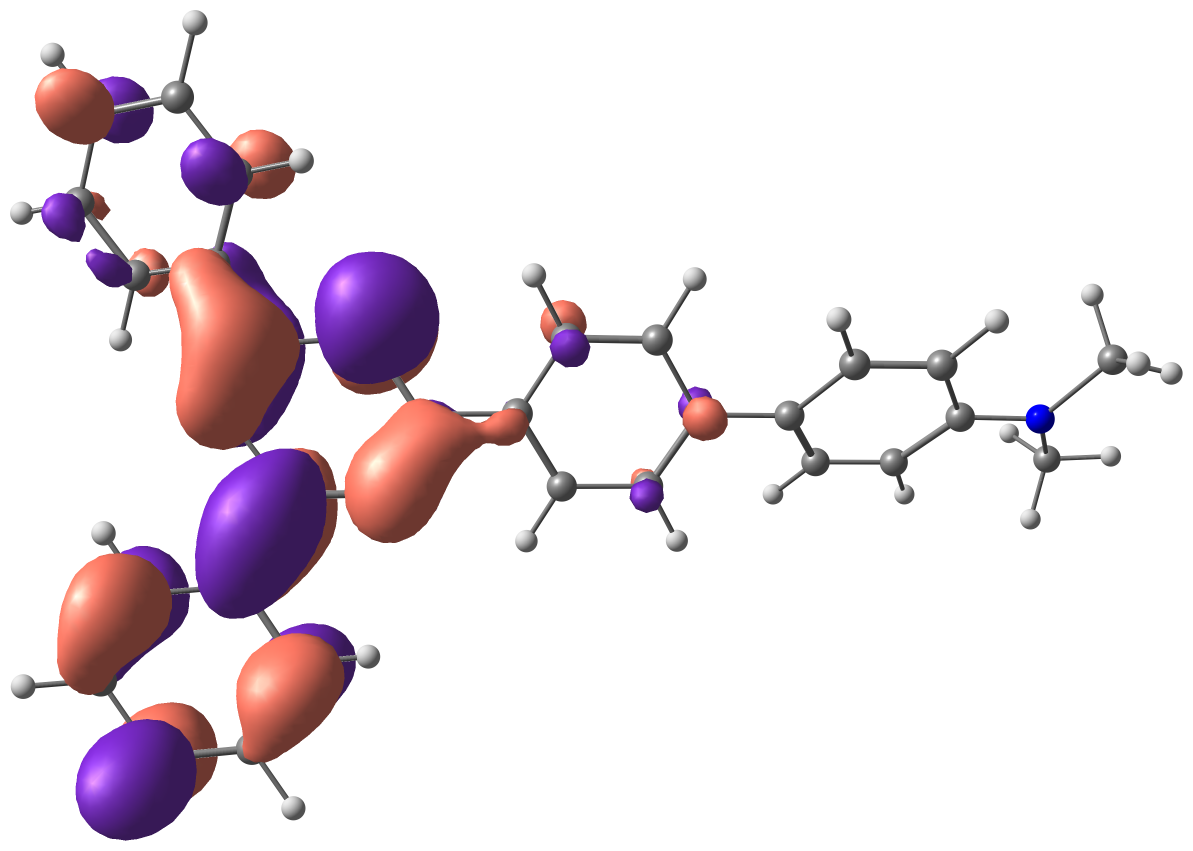  L + 1 (ACN) |
| 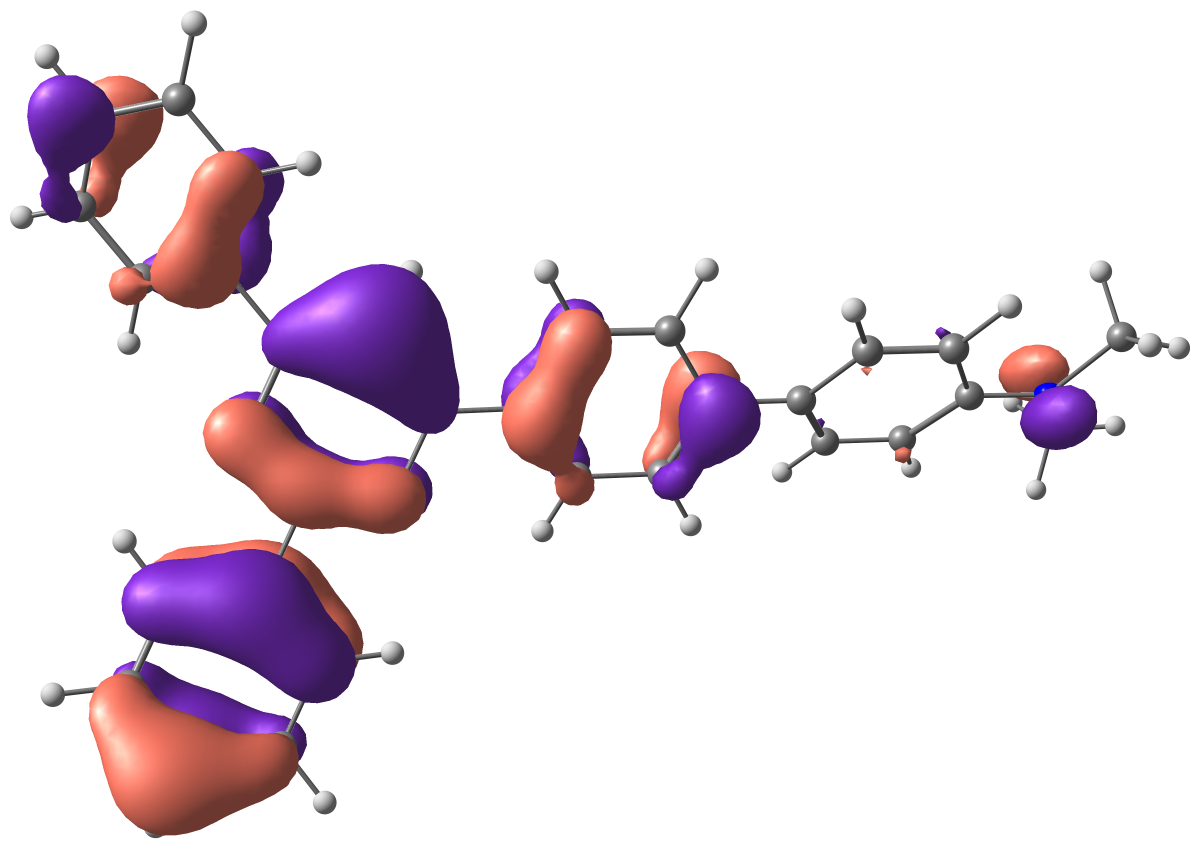  H – 1(DMSO) | 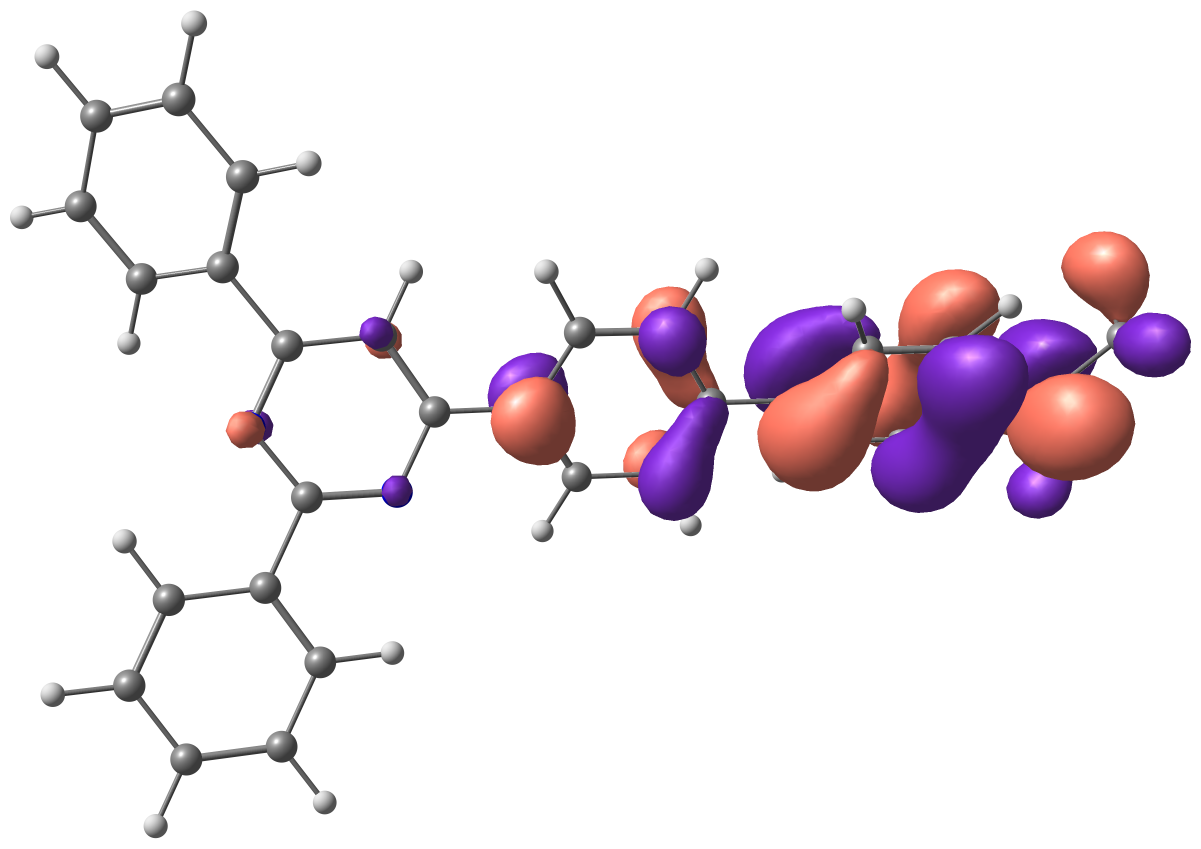  HOMO (DMSO) | 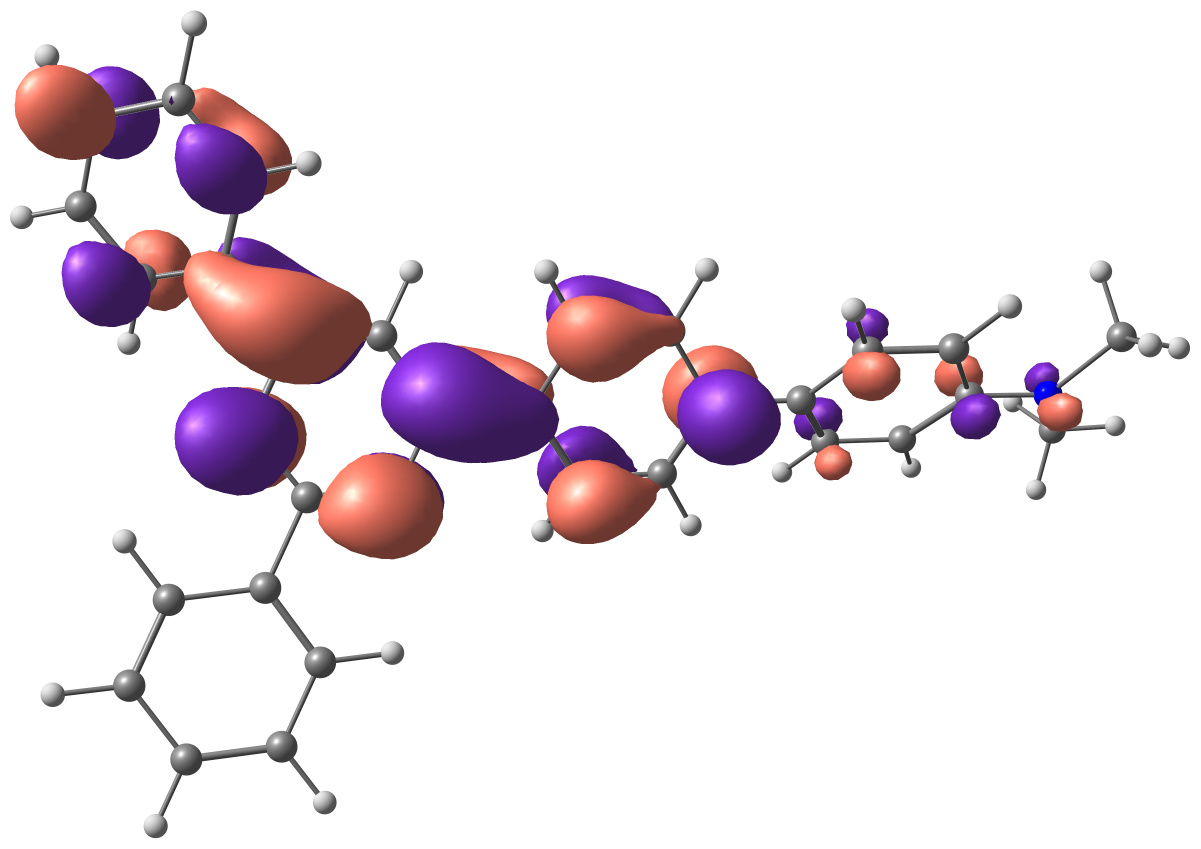  LUMO (DMSO) | 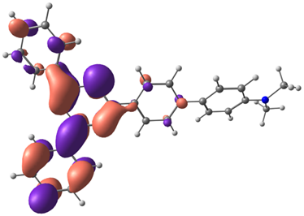  L + 1 (DMSO) |

**Figure S5.** Isosurface contour plots (±0.03 a.u.) calculated at the B3LYP/6-311G**(PCM) level for the frontier MOs of **D1** at the S_0_ minimum-energy geometry in Hx, Tol, ACN and DMSO.

| 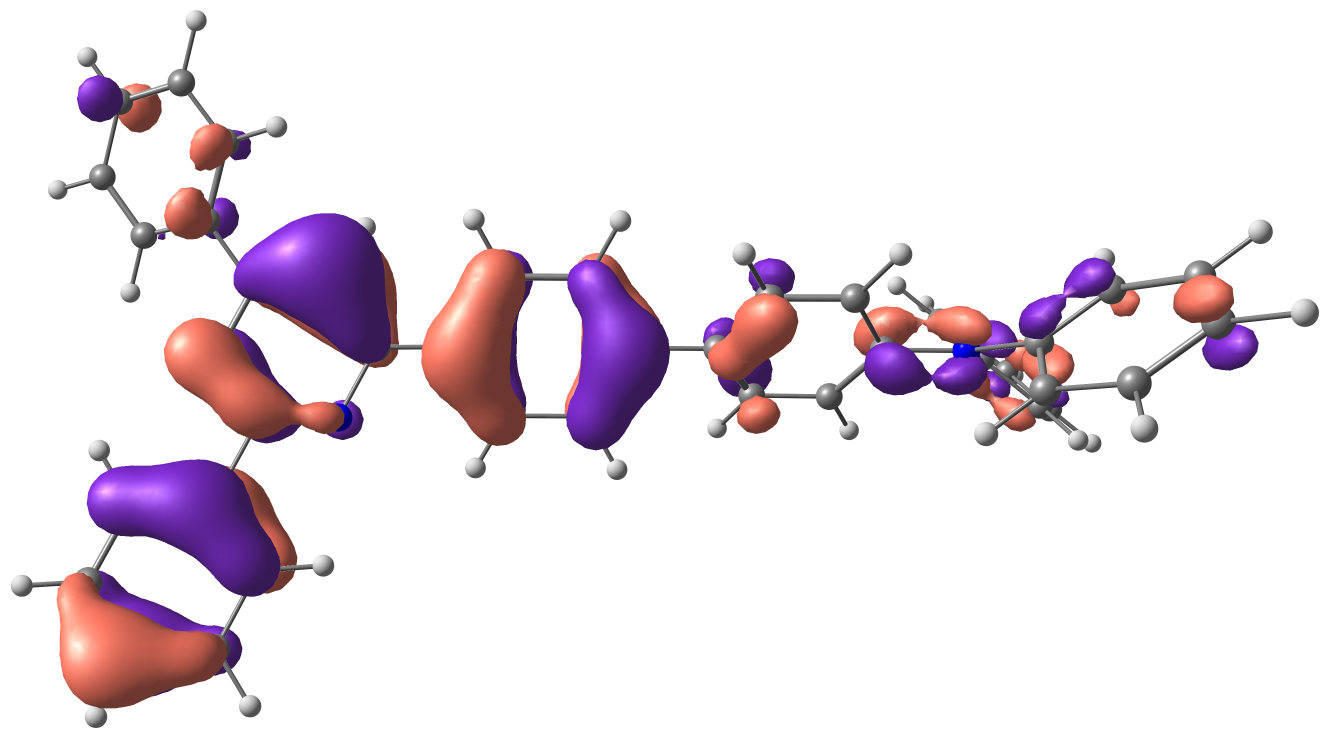  H – 1(Hx) | 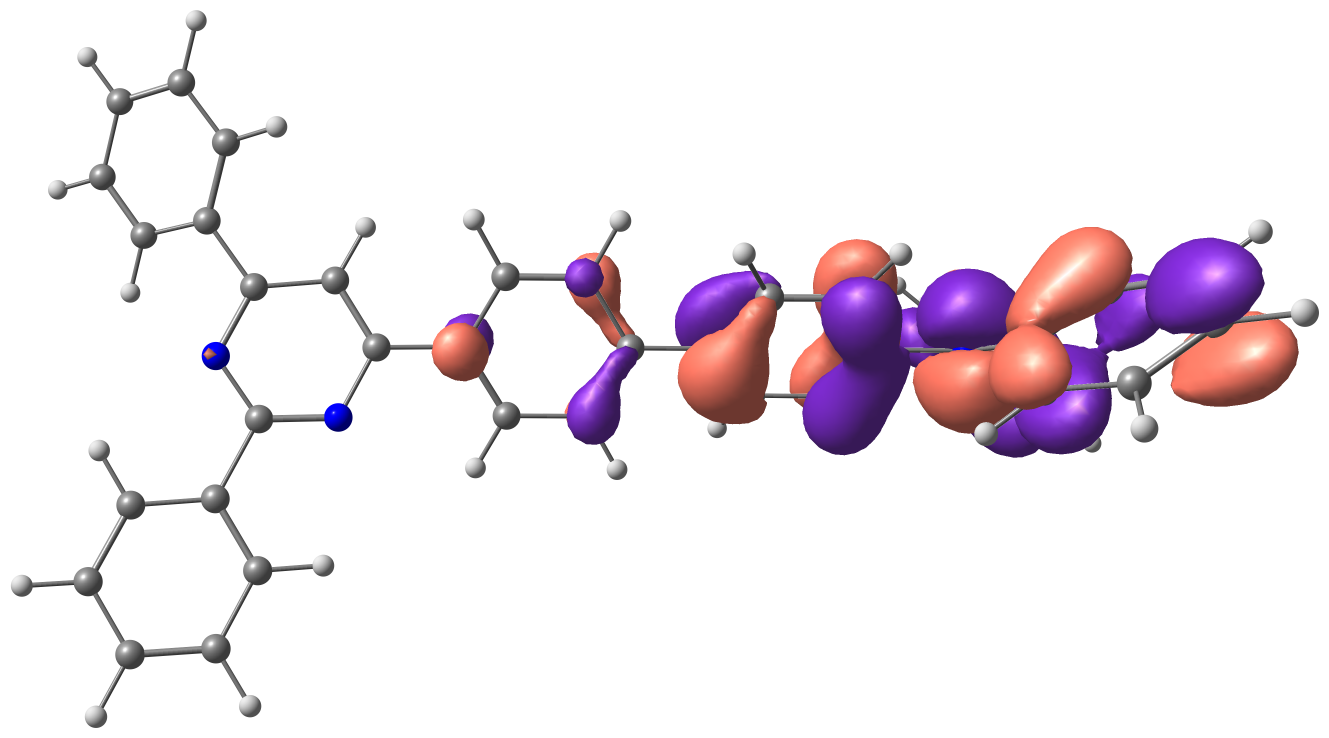  HOMO (Hx) | 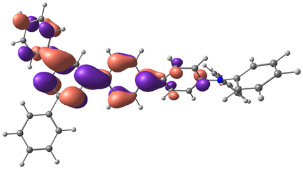  LUMO (Hx) | 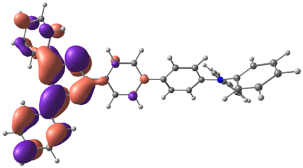  L + 1 (Hx) |
| --- | --- | --- | --- |
| 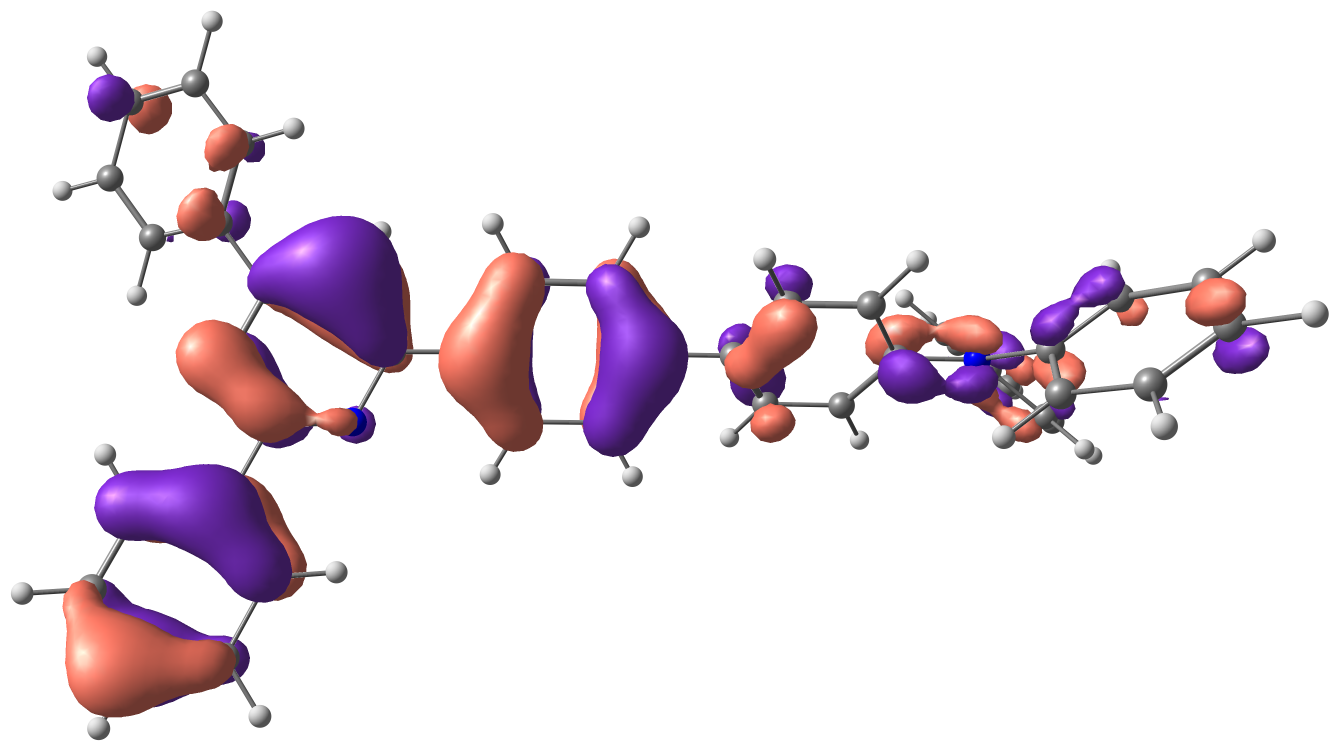  H – 1(Tol) | 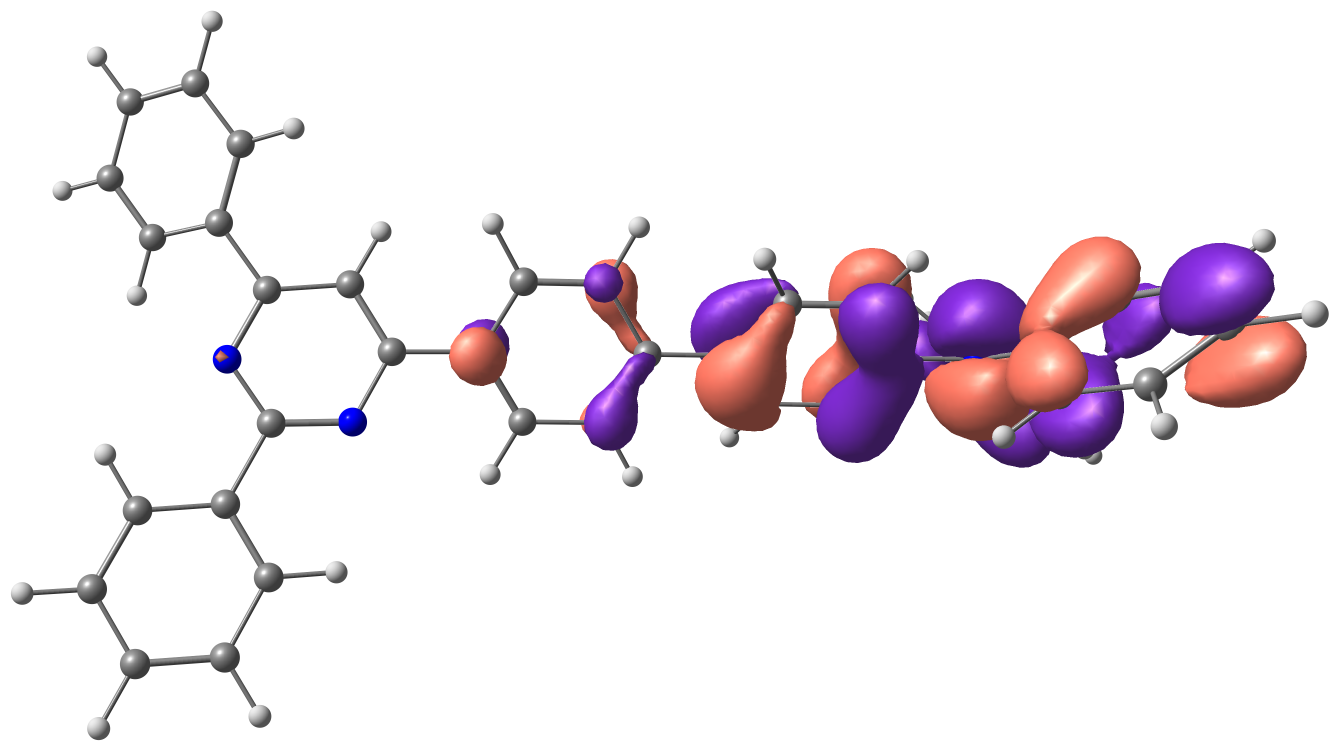  HOMO (Tol) | 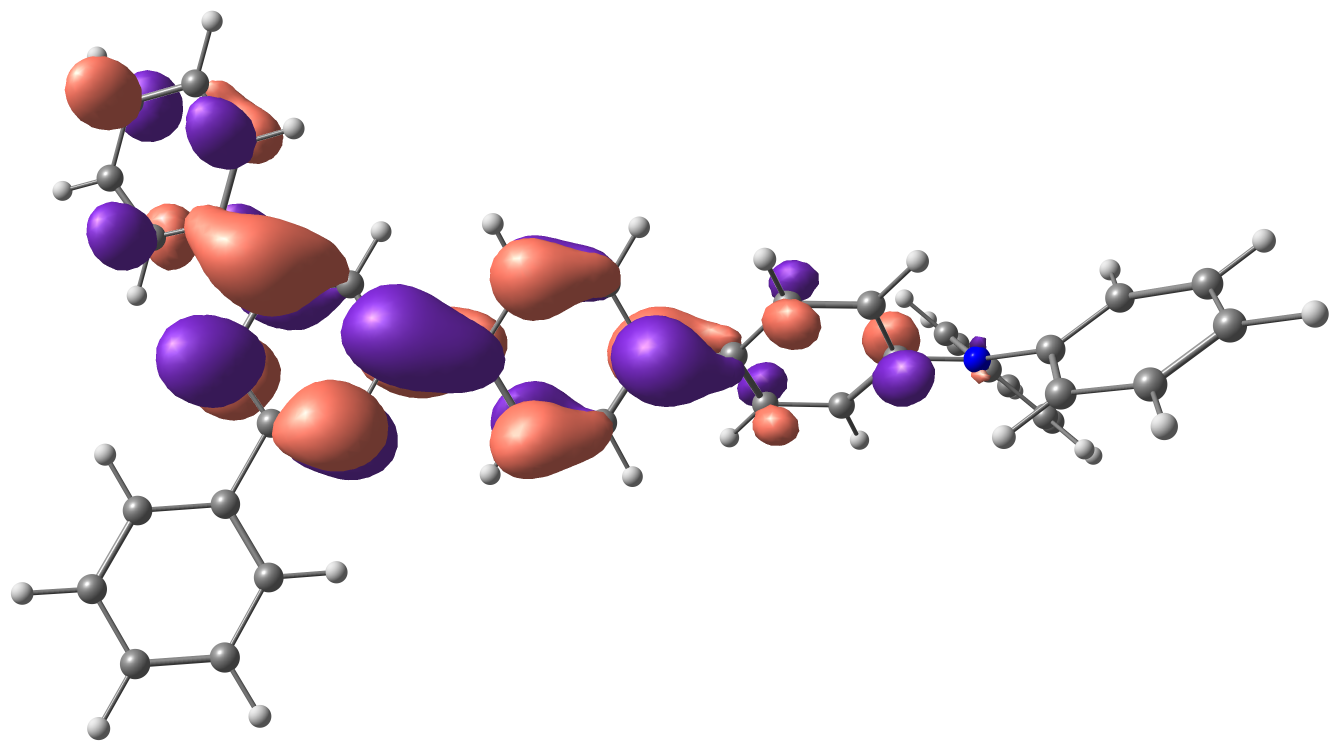  LUMO (Tol) | 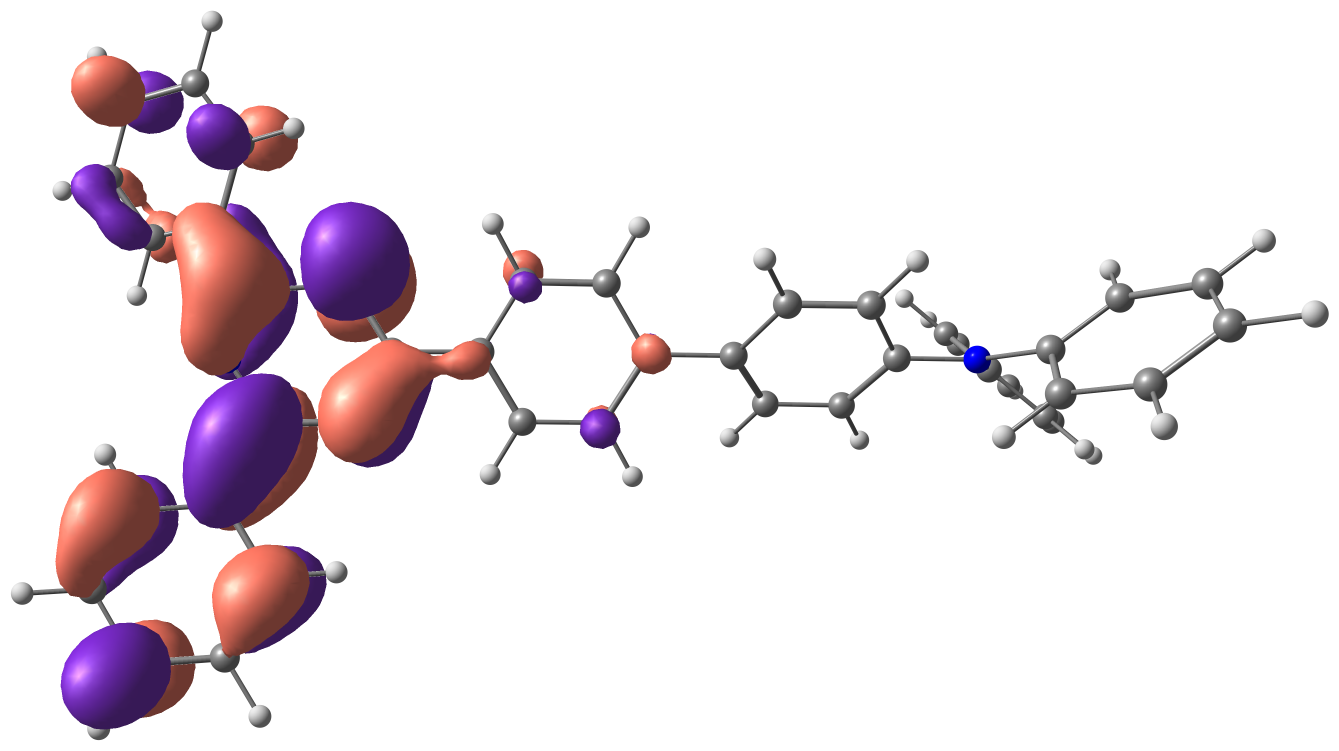  L + 1 (Tol) |
| 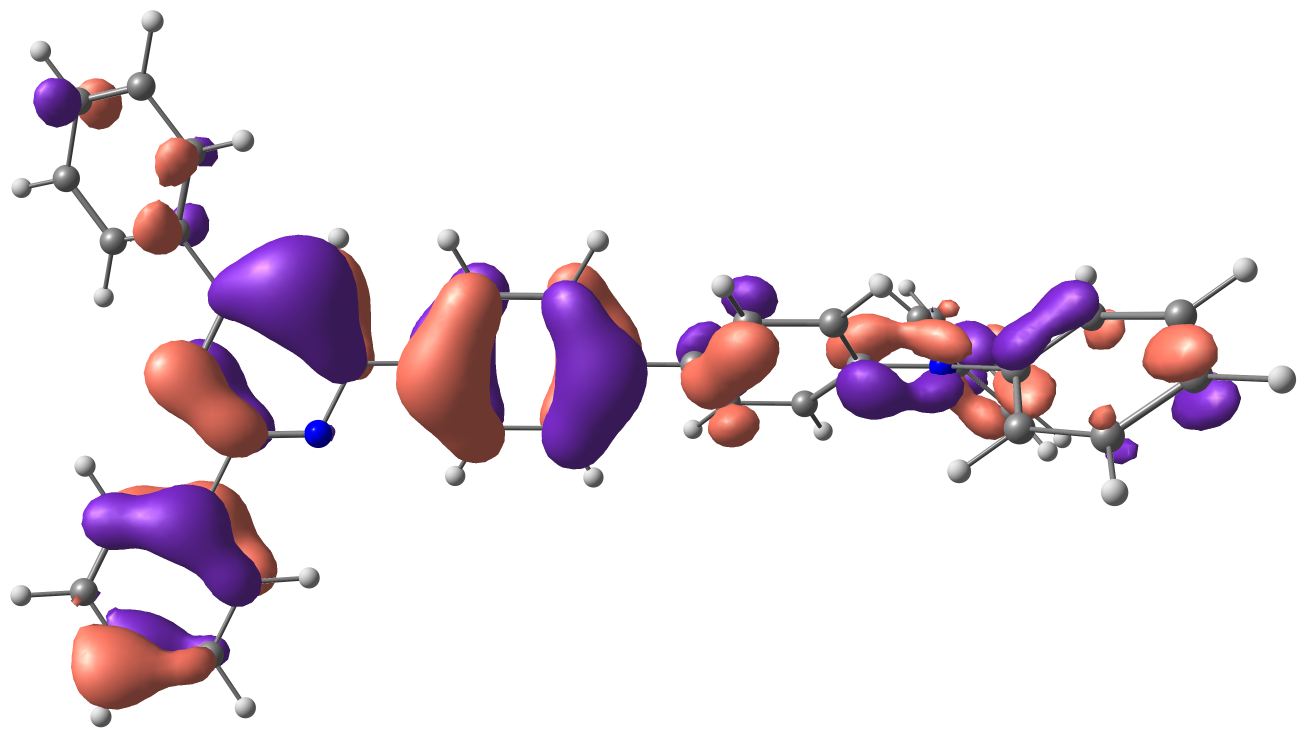  H – 1 (ACN) | 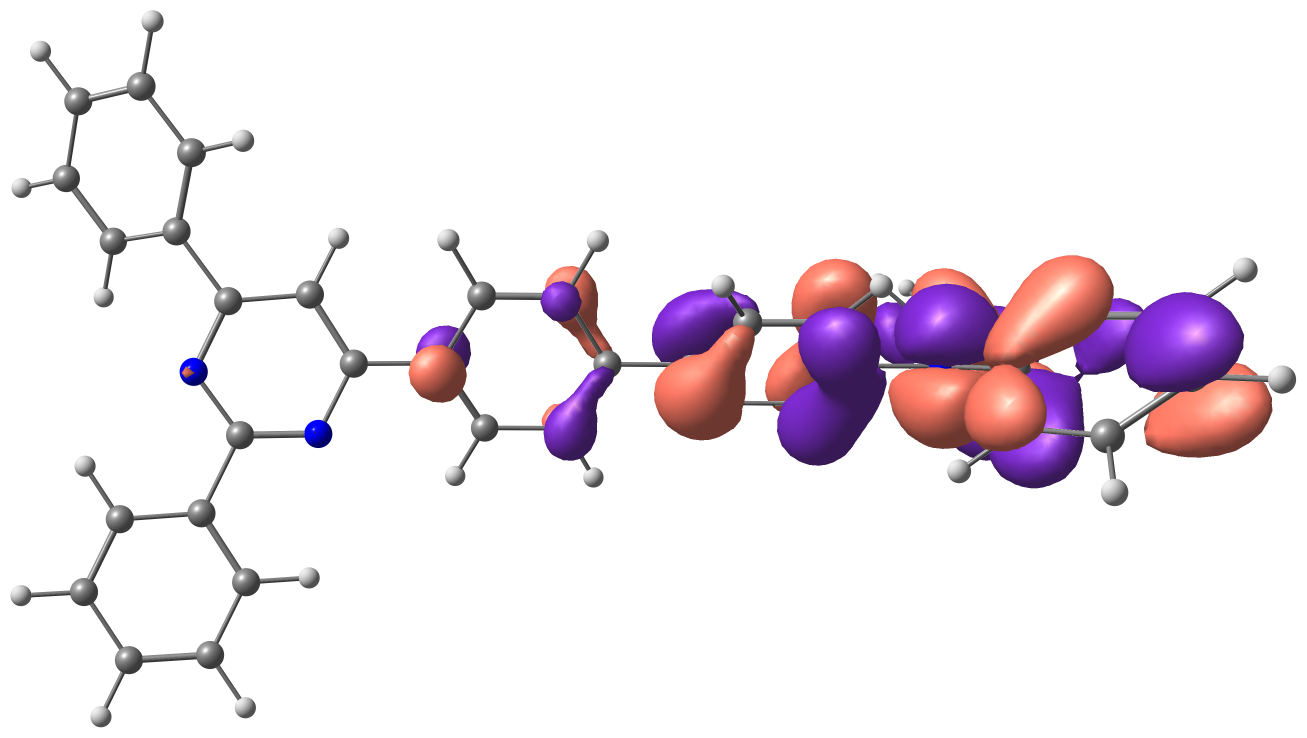  HOMO (ACN) | 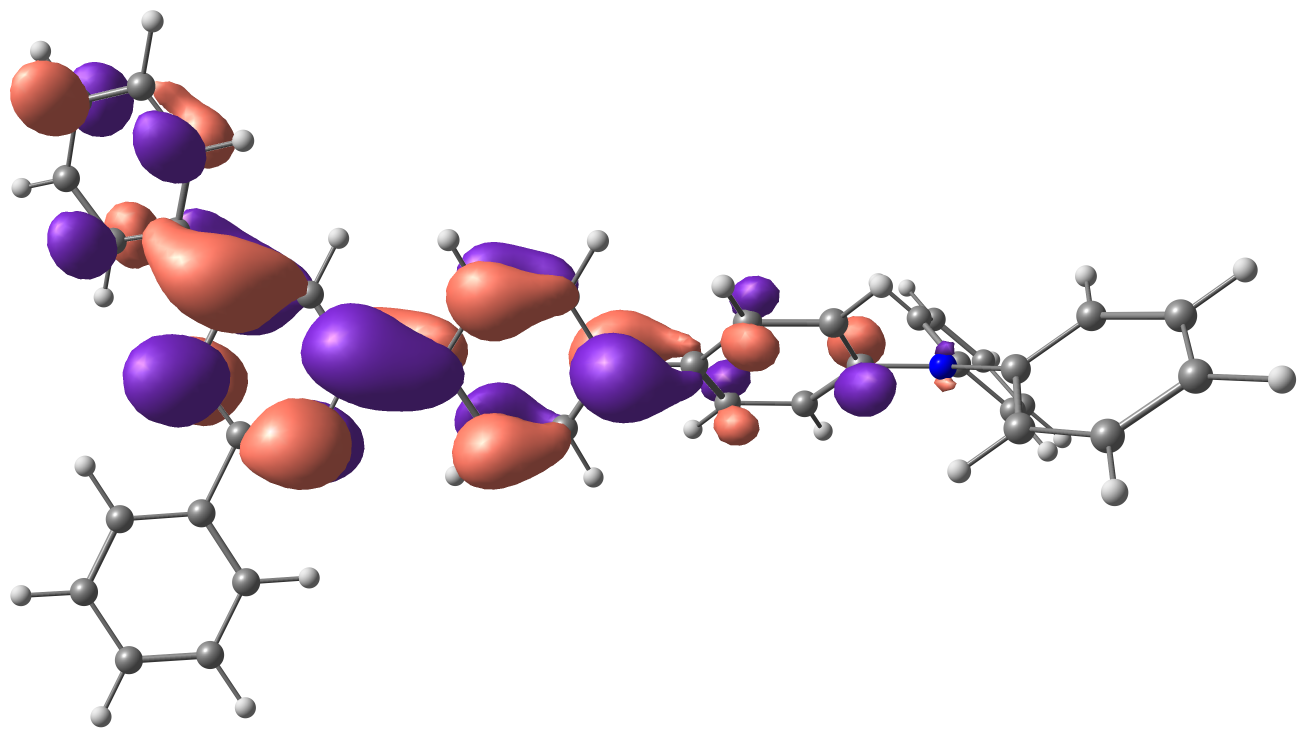  LUMO (ACN) | 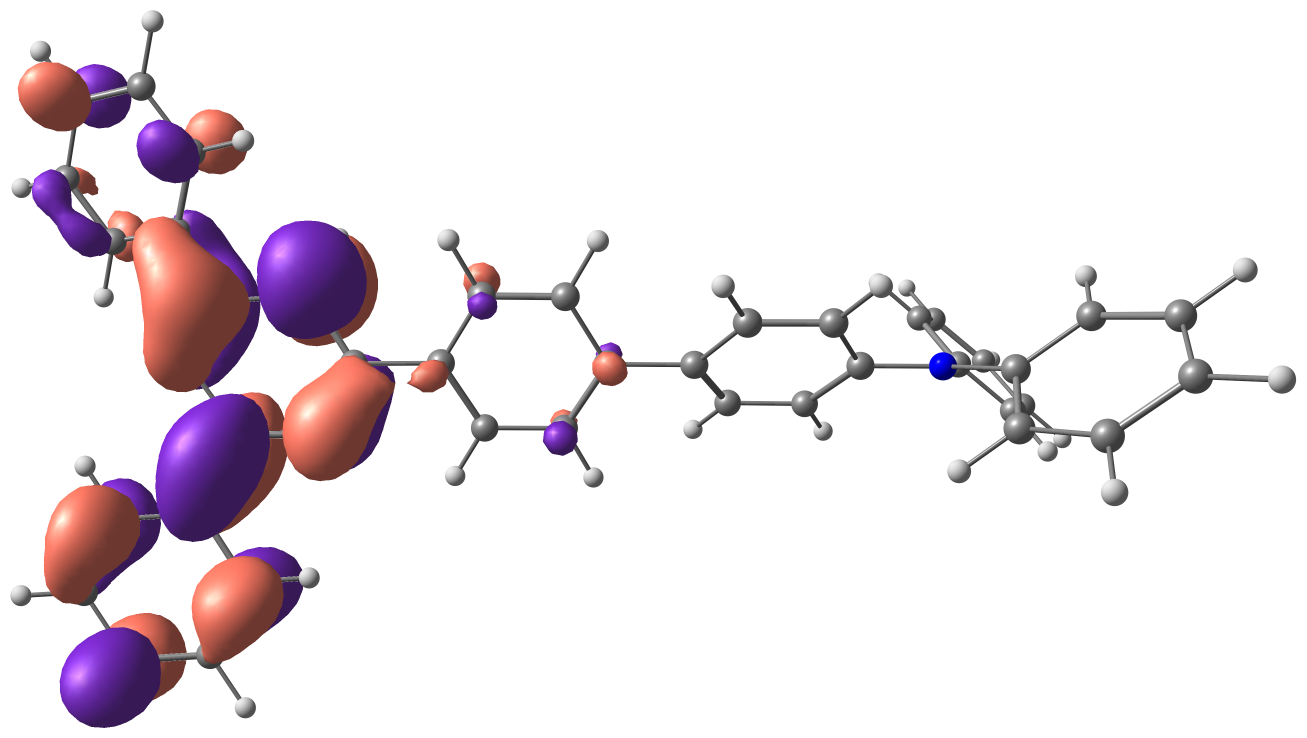  L + 1 (ACN) |
| 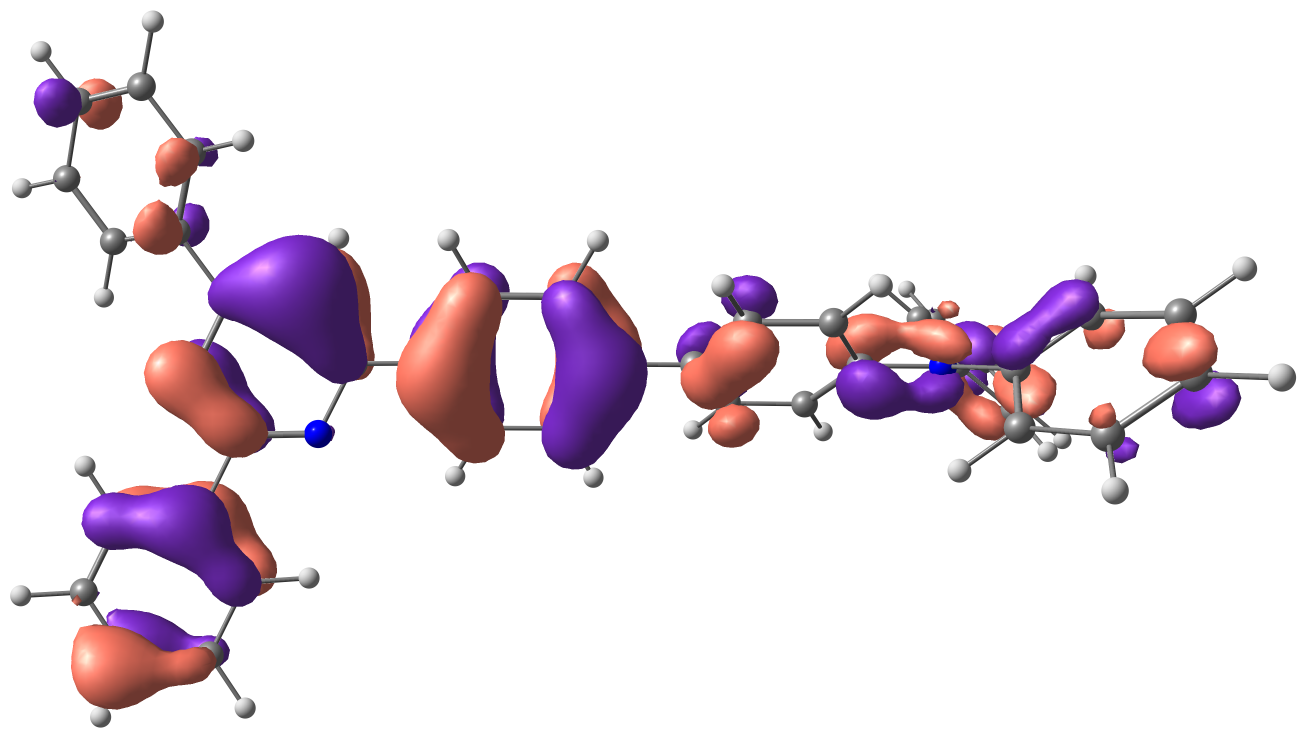  H – 1(DMSO) | 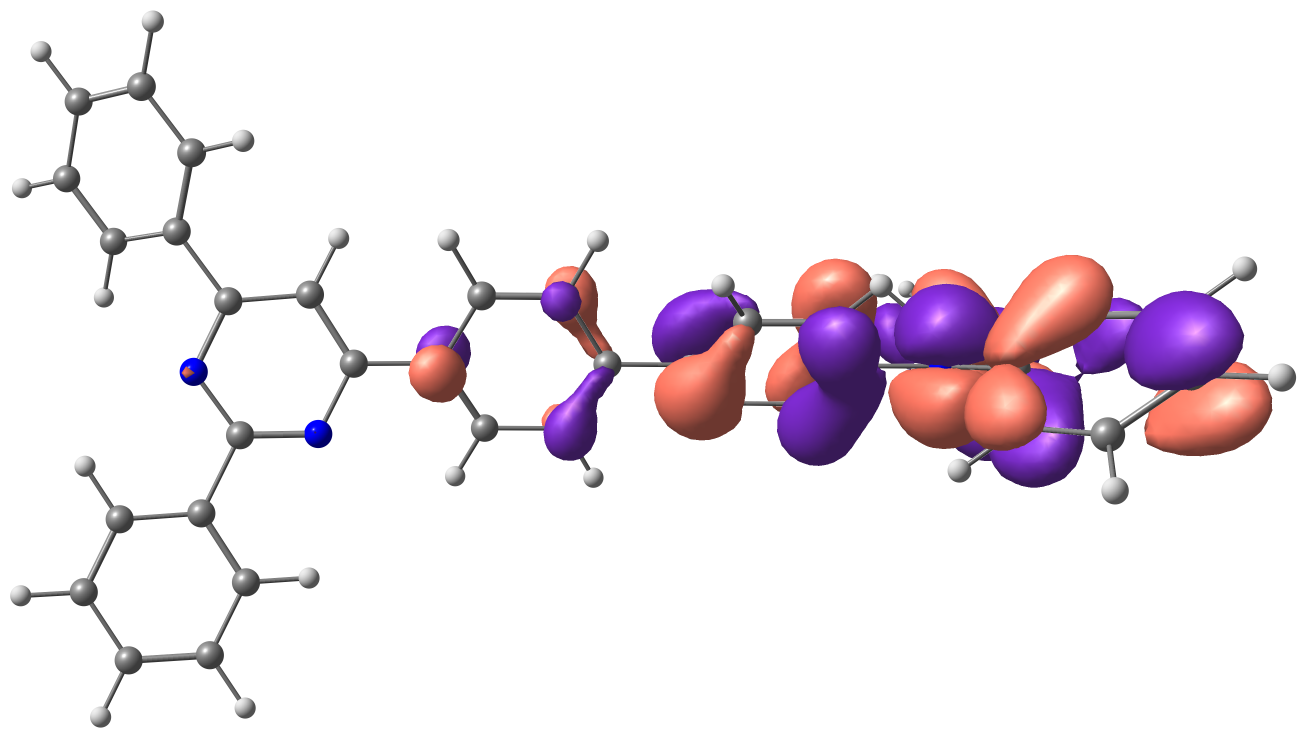  HOMO (DMSO) | 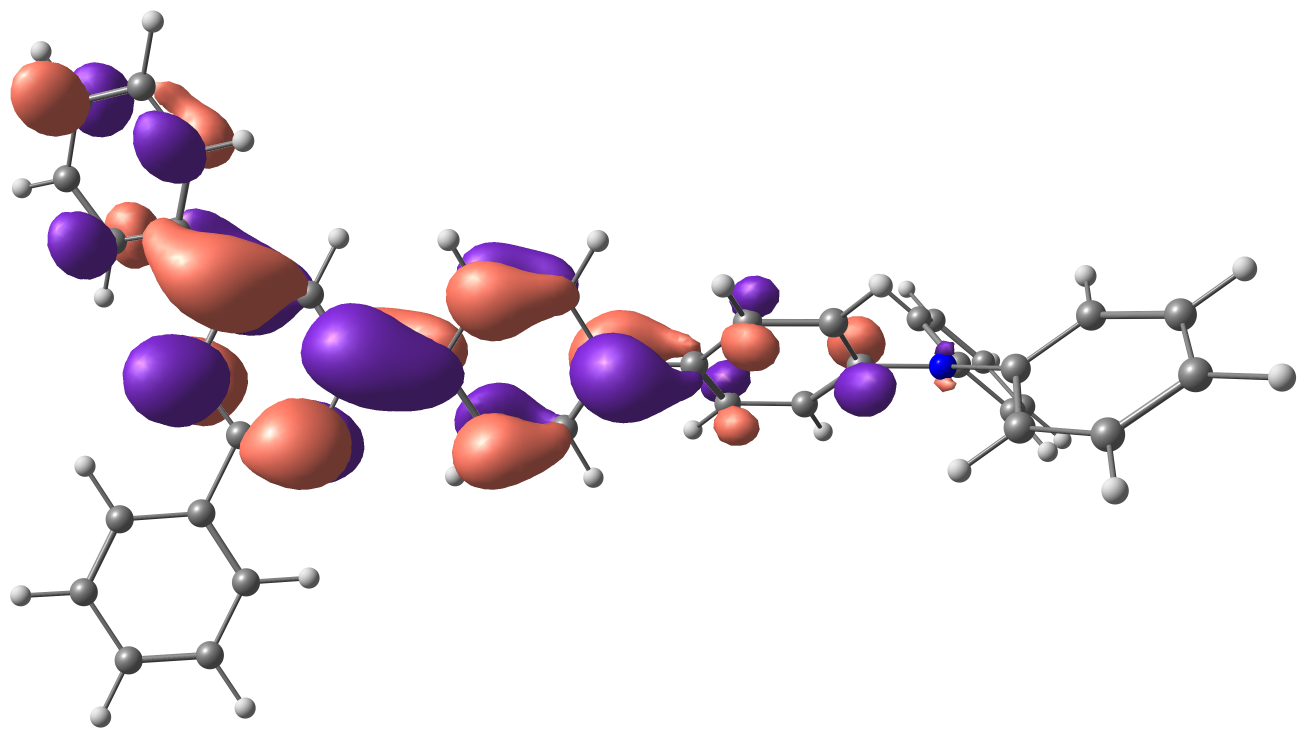  LUMO (DMSO) | 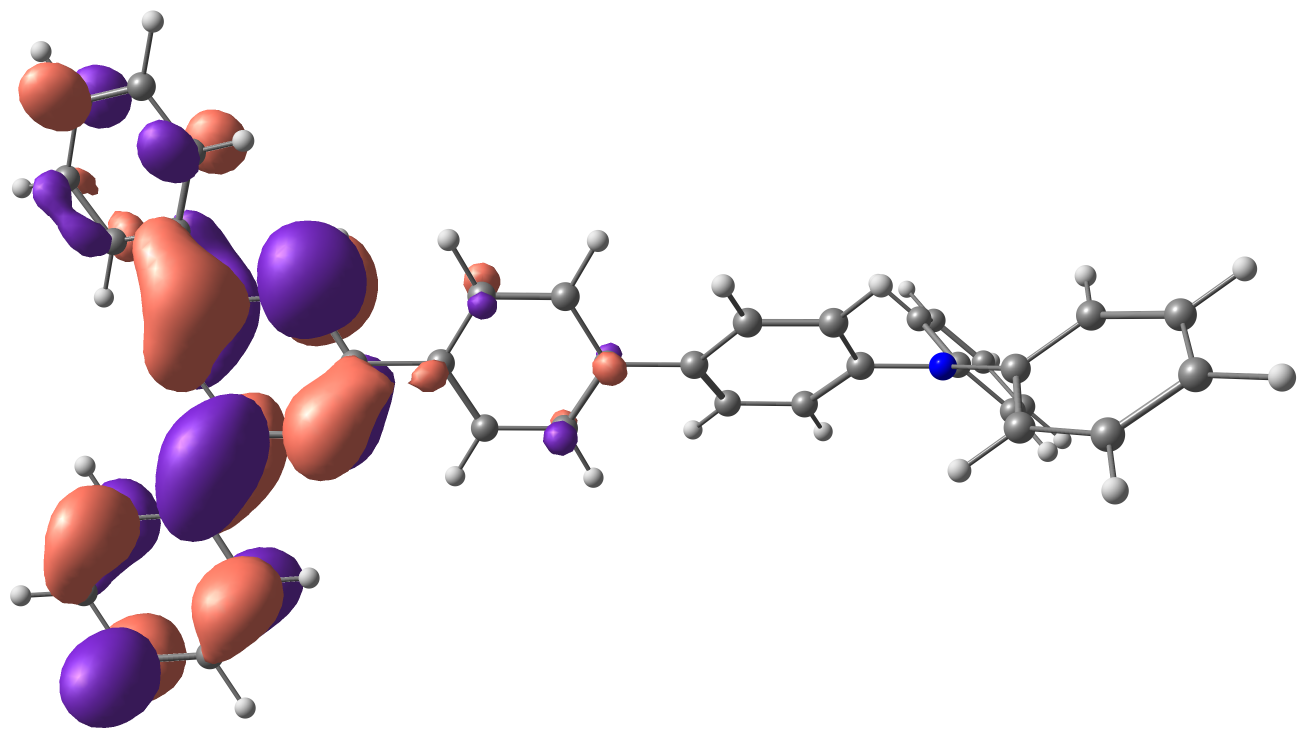  L + 1 (DMSO) |

**Figure S6.** Isosurface contour plots (±0.03 a.u.) calculated at the B3LYP/6-311G**(PCM) level for the frontier MOs of **D2** at the S_0_ minimum-energy geometry in Hx, Tol, ACN and DMSO.

**Table S3.** Main atomic contribution (higher than 2 % to the final wave function) to the HOMO and LUMO computed at the S_0_ minimum at the B3LYP/6-311G**(PCM) level of theory for Hx, Tol, ACN and DMSO solvents. **D1** and **D2** are divided in four molecular fragments as sketched in Scheme 1: pyrimidine environment (fragment 1 in yellow); phenyl attached to the pyrimidine (fragment 2 in red); phenyl attached to the diamine (fragment 3 in blue); diamine environment (fragment 4 in green).

| **Fragment** | **MO** | **D1** | | | | **D2** | | | |
| --- | --- | --- | --- | --- | --- | --- | --- | --- | --- |
|  |  | **Hx** | **Tol** | **ACN** | **DMSO** | **Hx** | **Tol** | **ACN** | **DMSO** |
| **1** | **HOMO** | 0.00 | 0.00 | 0.00 | 0.00 | 0.00 | 0.00 | 0.00 | 0.00 |
|  | **LUMO** | 0.66 | 0.66 | 0.65 | 0.65 | 0.57 | 0.57 | 0.58 | 0.58 |
| **2** | **HOMO** | 0.11 | 0.11 | 0.11 | 0.11 | 0.02 | 0.02 | 0.08 | 0.02 |
|  | **LUMO** | 0.20 | 0.20 | 0.21 | 0.21 | 0.23 | 0.23 | 0.22 | 0.22 |
| **3** | **HOMO** | 0.44 | 0.42 | 0.42 | 0.42 | 0.24 | 0.24 | 0.18 | 0.24 |
|  | **LUMO** | 0.00 | 0.00 | 0.00 | 0.00 | 0.03 | 0.03 | 0.02 | 0.02 |
| **4** | **HOMO** | 0.34 | 0.32 | 0.34 | 0.34 | 0.62 | 0.62 | 0.61 | 0.61 |
|  | **LUMO** | 0.00 | 0.00 | 0.00 | 0.00 | 0.00 | 0.00 | 0.00 | 0.00 |

**Table S4.** Vertical energies (eV), oscillator strengths, dipole moments (D), and main monoexcitations computed at the S_0_ minimum using the TD-DFT B3LYP/6-311G** approach for the S_1_, S_2_, S_3_, and S_4_ states of **D1**. Solvent effects (Hx, Tol, ACN, and DMSO) were simulated with the PCM methodology, without using the CLR approximation.

| **Solvent** | **State** | ***E* (eV)** | ***f*** | ***μ* (D)** | **Monoexcitation (%)** |
| --- | --- | --- | --- | --- | --- |
| **Hx** | **S_1_** | 3.01 | 0.6592 | 32.6357 | H → L (99) |
|  | **S_2_** | 3.27 | 0.0746 | 40.4951 | H → L + 1 (99) |
|  | **S_3_** | 3.88 | 0.0026 | 2.7283 | H - 3 → L (66)  H - 2 → L (20) |
|  | **S_4_** | 3.95 | 0.2339 | 4.9869 | H - 1 → L (90) |
| **Tol** | **S_1_** | 2.98 | 0.6836 | 33.2353 | H → L (99) |
|  | **S_2_** | 3.25 | 0.0704 | 41.6314 | H → L + 1 (99) |
|  | **S_3_** | 3.89 | 0.0027 | 2.7776 | H - 3 → L (67)  H - 2 → L (17) |
|  | **S_4_** | 3.95 | 0.2656 | 5.2650 | H - 1 → L (88) |
| **ACN** | **S_1_** | 2.88 | 0.6559 | 35.9522 | H → L (99) |
|  | **S_2_** | 3.13 | 0.0647 | 46.6985 | H → L + 1 (99) |
|  | **S_3_** | 3.92 | 0.0029 | 10.1949 | H - 3 → L (62)  H - 5 → L (15) |
|  | **S_4_** | 3.94 | 0.4232 | 3.3199 | H - 1 → L (72)  H → L + 2 (21) |
| **DMSO** | **S_1_** | 2.88 | 0.6726 | 36.0125 | H → L (99) |
|  | **S_2_** | 3.13 | 0.0629 | 46.8024 | H → L + 1 (99) |
|  | **S_3_** | 3.92 | 0.0032 | 10.6561 | H - 3 → L (62)  H - 5 → L (16) |
|  | **S_4_** | 3.94 | 0.4365 | 3.1145 | H - 1 → L (72)  H → L + 2 (22) |

**Table S5.** Vertical energies (eV), oscillator strengths, dipole moments (D), and main monoexcitations computed at the S_0_ minimum using the TD-DFT B3LYP/6-311G** approach for the S_1_, S_2_, S_3_, and S_4_ states of **D2** Solvent effects (Hx, Tol, ACN, and DMSO) were simulated with the PCM methodology, without using the CLR approximation.

| **Solvent** | **State** | ***E* (eV)** | ***f*** | ***μ* (D)** | **Monoexcitation (%)** |
| --- | --- | --- | --- | --- | --- |
| **Hx** | **S_1_** | 2.91 | 0.6431 | 34.6579 | H → L (97) |
|  | **S_2_** | 3.26 | 0.0345 | 47.4933 | H → L + 1 (98) |
|  | **S_3_** | 3.74 | 0.0167 | 2.1291 | H → L + 3 (98) |
|  | **S_4_** | 3.80 | 0.5526 | 23.0205 | H → L + 2 (76)  H – 1→ L (16) |
| **Tol** | **S_1_** | 2.90 | 0.6606 | 35.1733 | H → L (97) |
|  | **S_2_** | 3.25 | 0.0337 | 48.3842 | H → L + 1 (98) |
|  | **S_3_** | 3.73 | 0.0181 | 2.2359 | H → L + 3 (97) |
|  | **S_4_** | 3.79 | 0.5473 | 24.4310 | H → L + 2 (79)  H – 1→ L (13) |
| **ACN** | **S_1_** | 2.86 | 0.6350 | 37.3961 | H → L (97) |
|  | **S_2_** | 3.19 | 0.0307 | 52.1116 | H → L + 1 (98) |
|  | **S_3_** | 3.74 | 0.0167 | 3.8128 | H → L + 3 (98) |
|  | **S_4_** | 3.77 | 0.5460 | 27.1132 | H → L + 2 (84) |
| **DMSO** | **S_1_** | 2.85 | 0.6481 | 37.4330 | H → L (97) |
|  | **S_2_** | 3.19 | 0.0304 | 52.1812 | H → L + 1 (98) |
|  | **S_3_** | 3.74 | 0.0177 | 4.2801 | H → L + 3 (97) |
|  | **S_4_** | 3.76 | 0.5459 | 26.5819 | H → L + 2 (84) |

| Solvent | Transition | | | |
| --- | --- | --- | --- | --- |
|  | S_0_-S_1_ | | | |
| Hx | 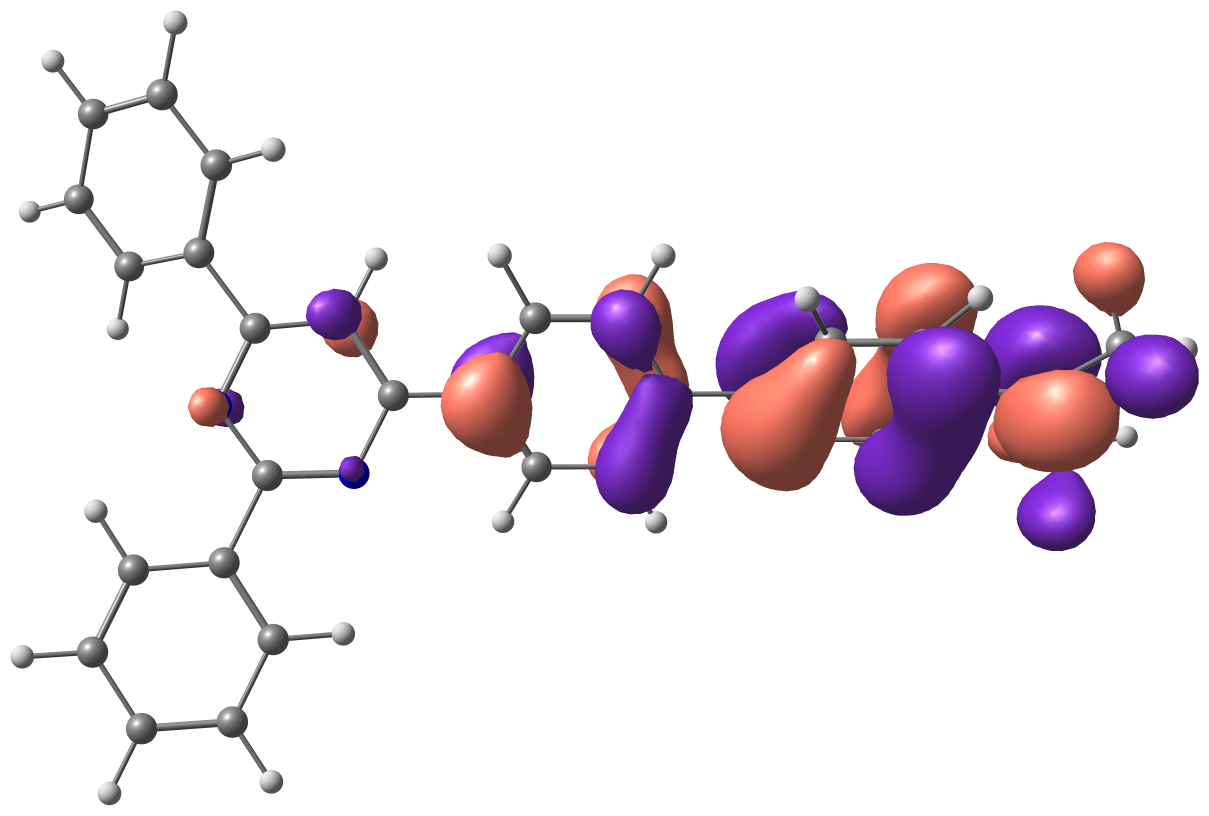 | | 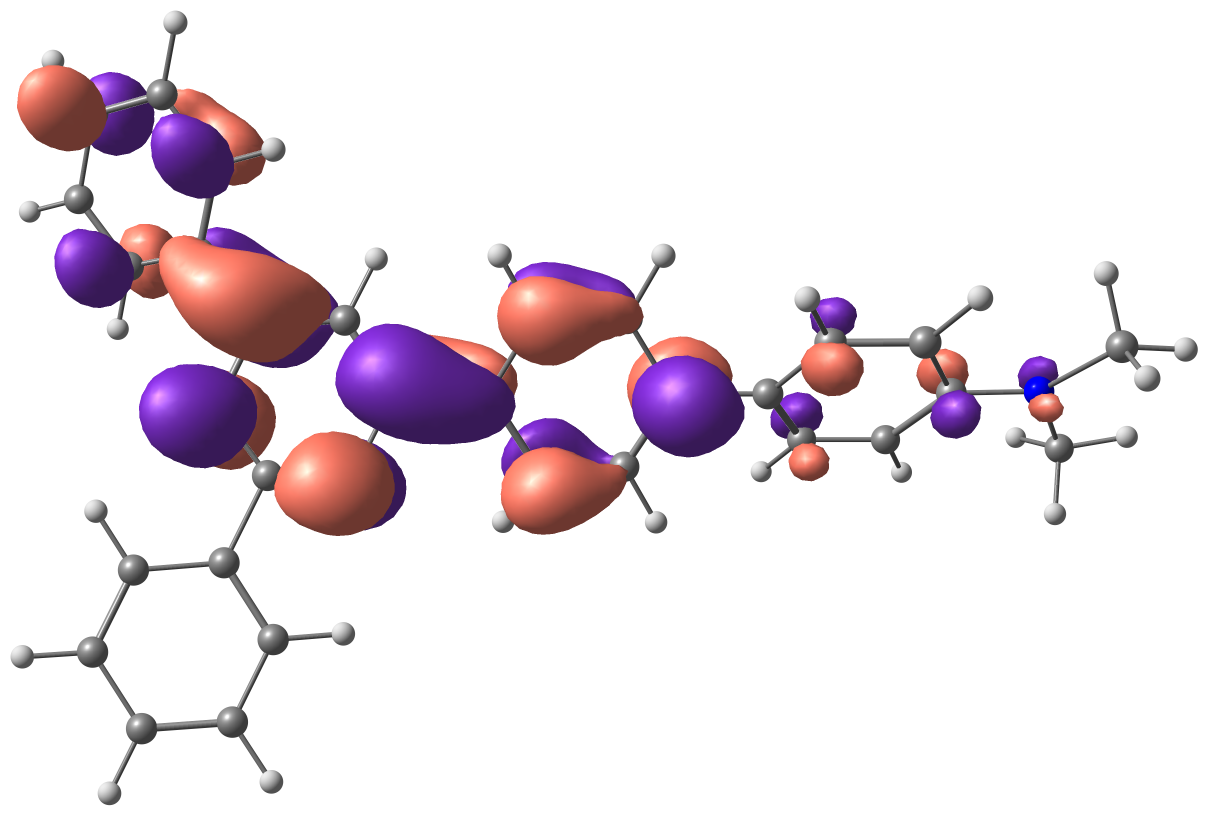 | |
|  | S_0_-S_4_ | | | |
|  | 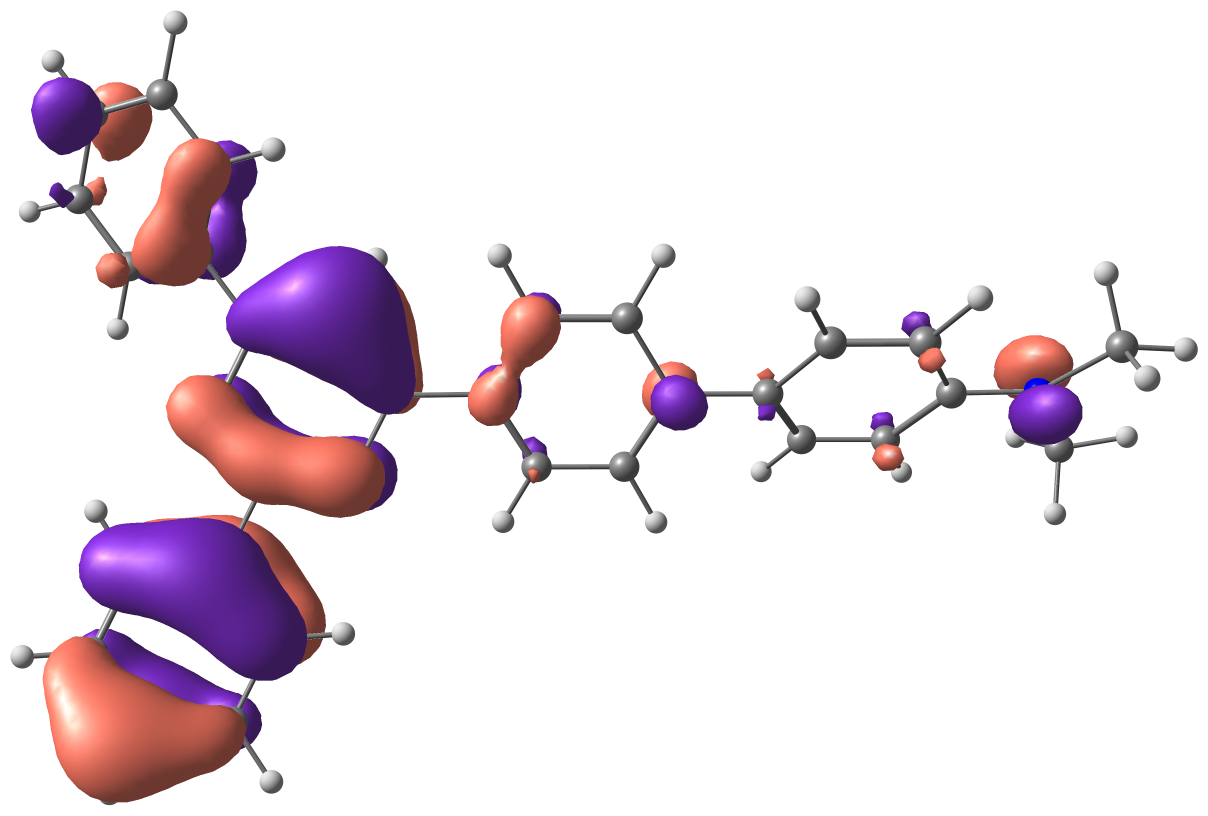 | | 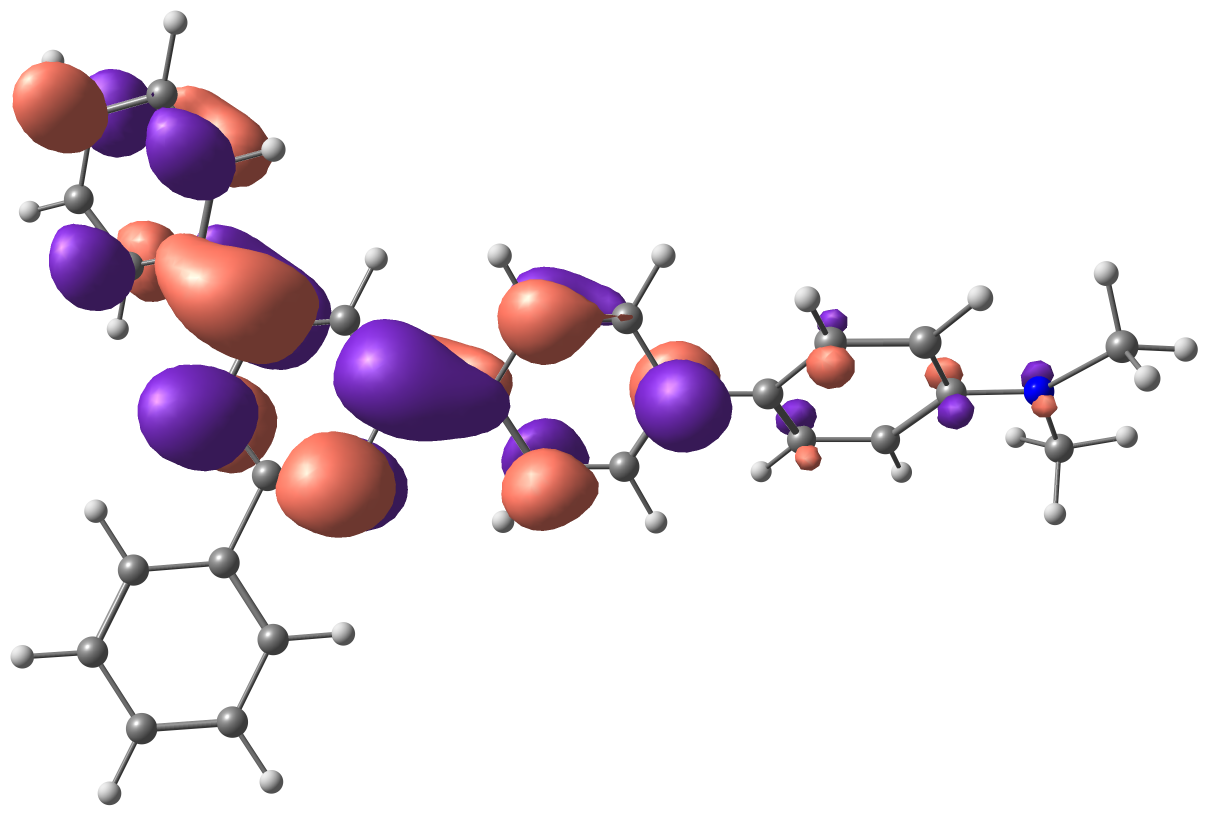 | |
| DMSO | S_0_-S_1_ | | | |
|  | 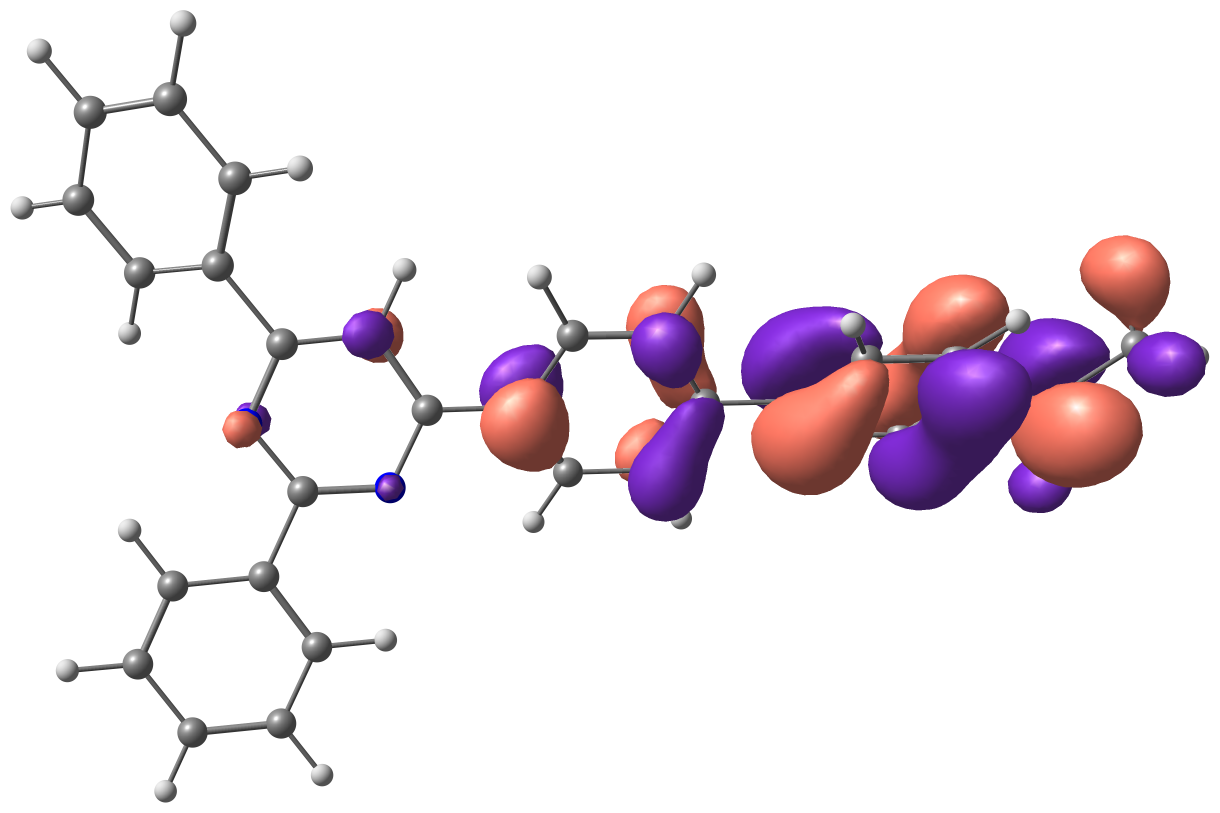 | | 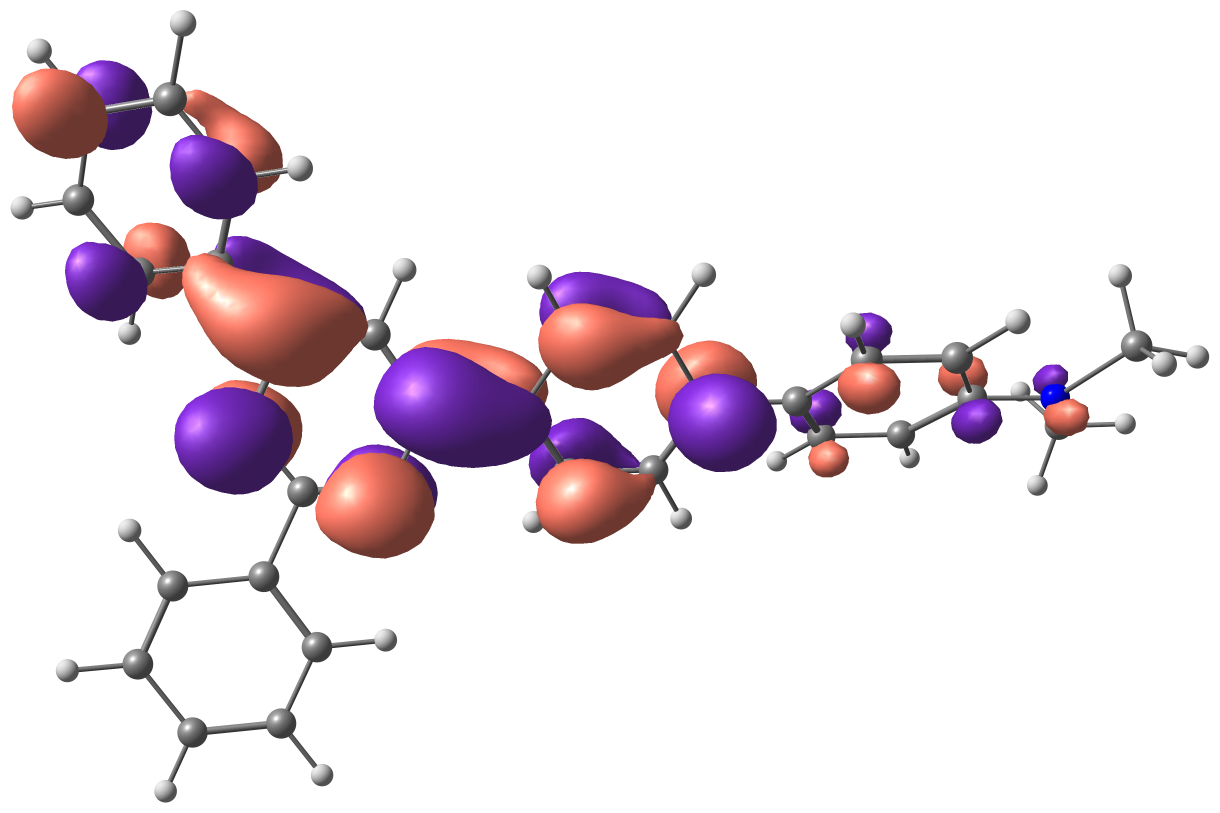 | |
|  | S_0_-S_4_ | | | |
|  | 1^rs^ pair (75 %) | | 2^nd^ pair (22 %) | |
|  | 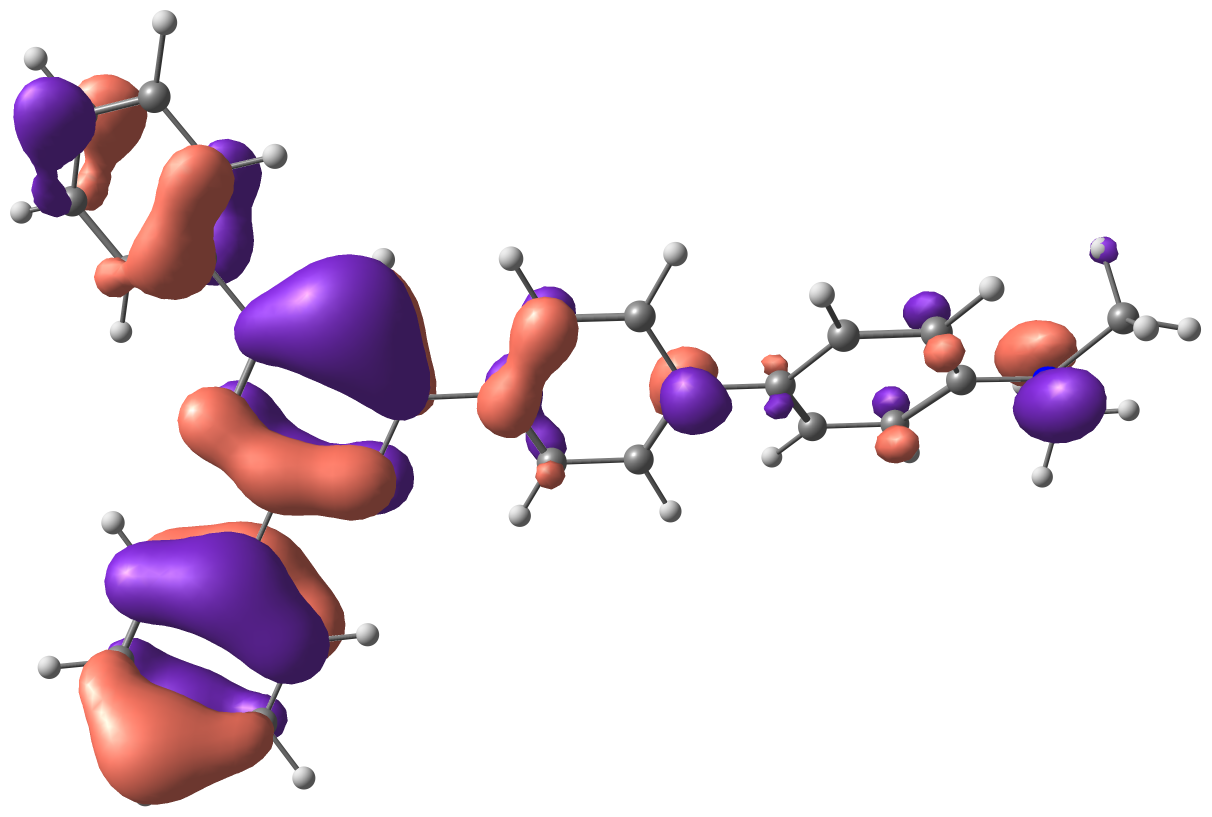 | 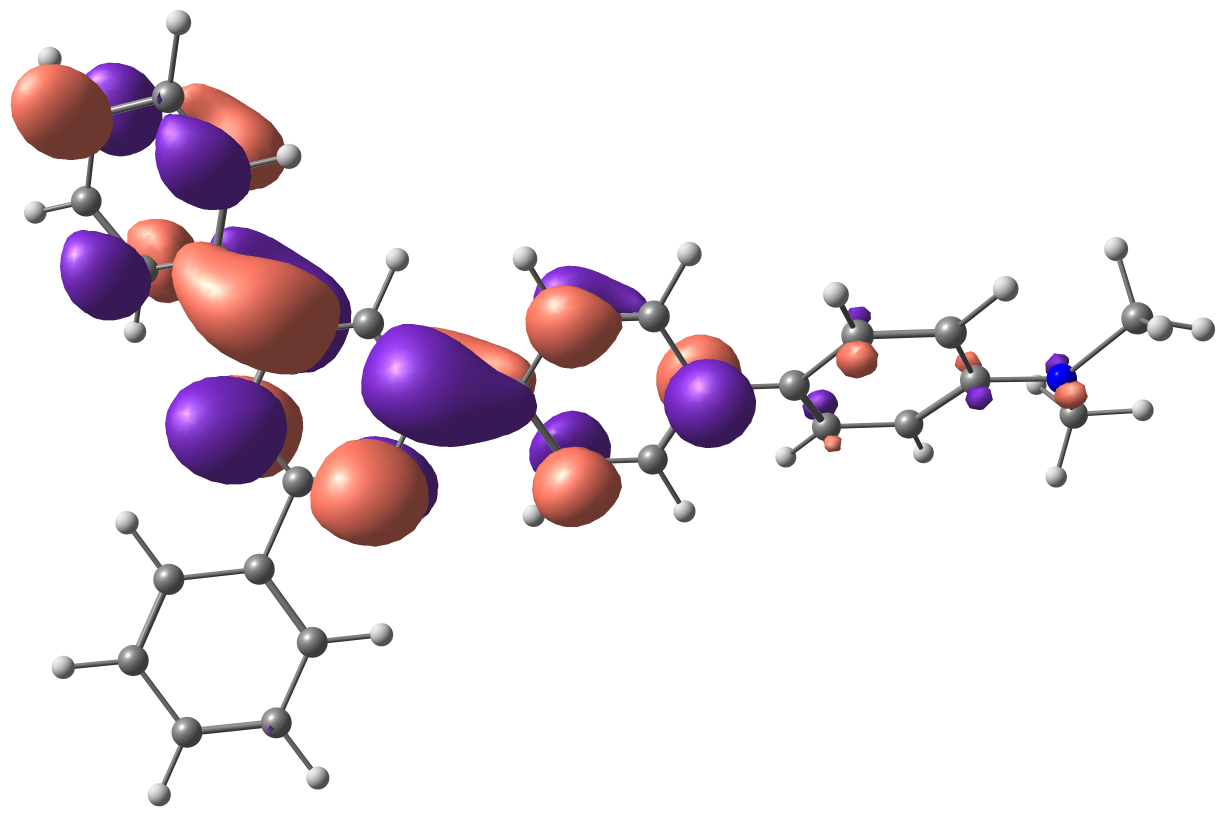 | 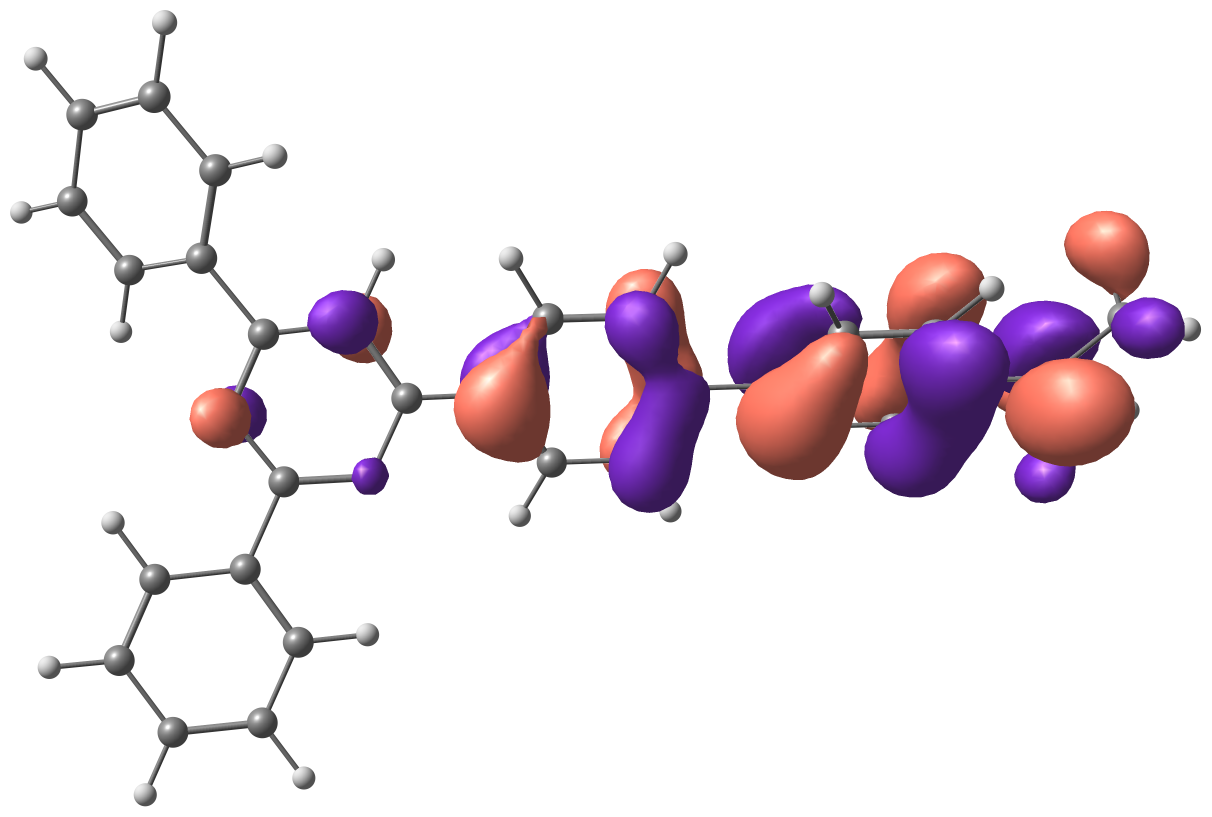 | 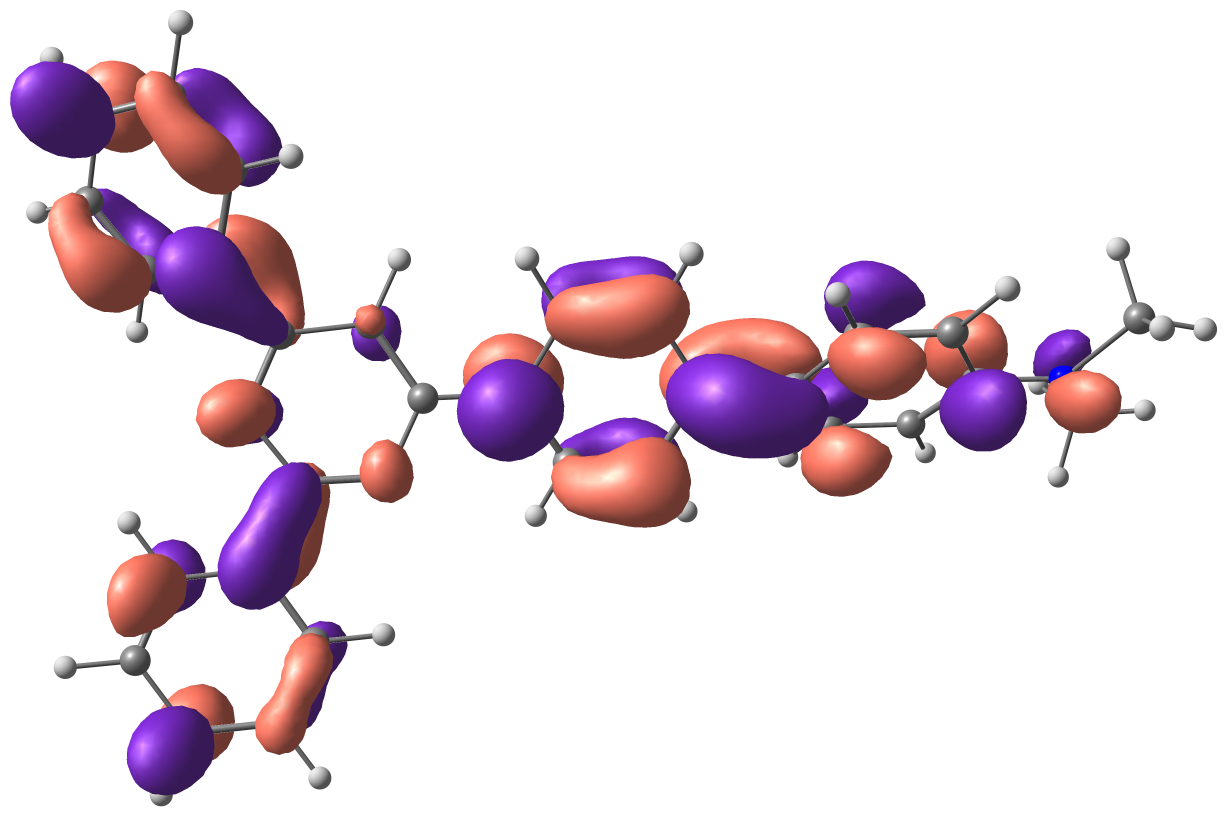 |

**Figure S7.** Natural transition orbital (NTO) pairs (isosurface contour plots: ±0.03 a.u.) describing the electronic transitions to the S_1_ and S_4_ excited states at the S_0_ minimum-energy geometry for the **D1** molecule. Computed at the TD-DFT B3LYP/6-311G**(PCM) level of theory for Hx and DMSO solvents.

| Solvent | Transition | |
| --- | --- | --- |
|  | S_0_-S_1_ (S_1,0_ geometry) | |
| Hx | 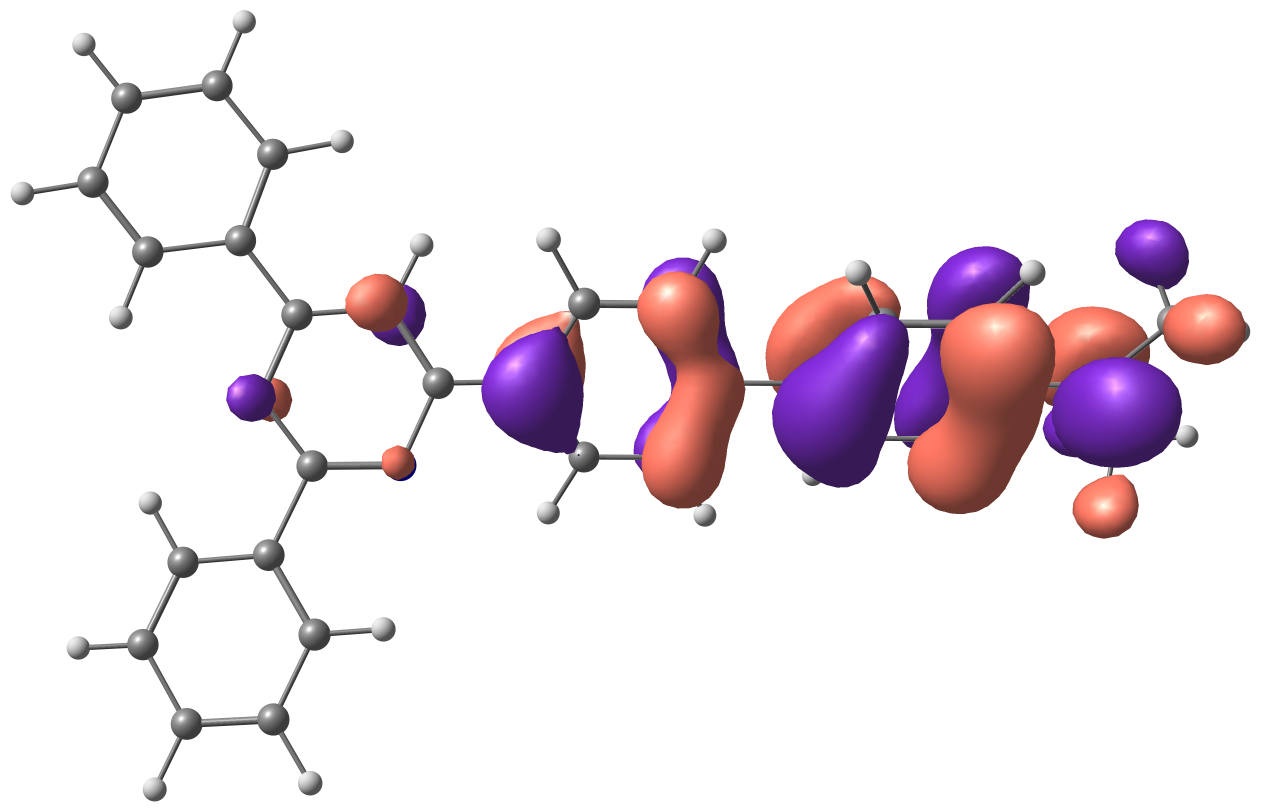 | 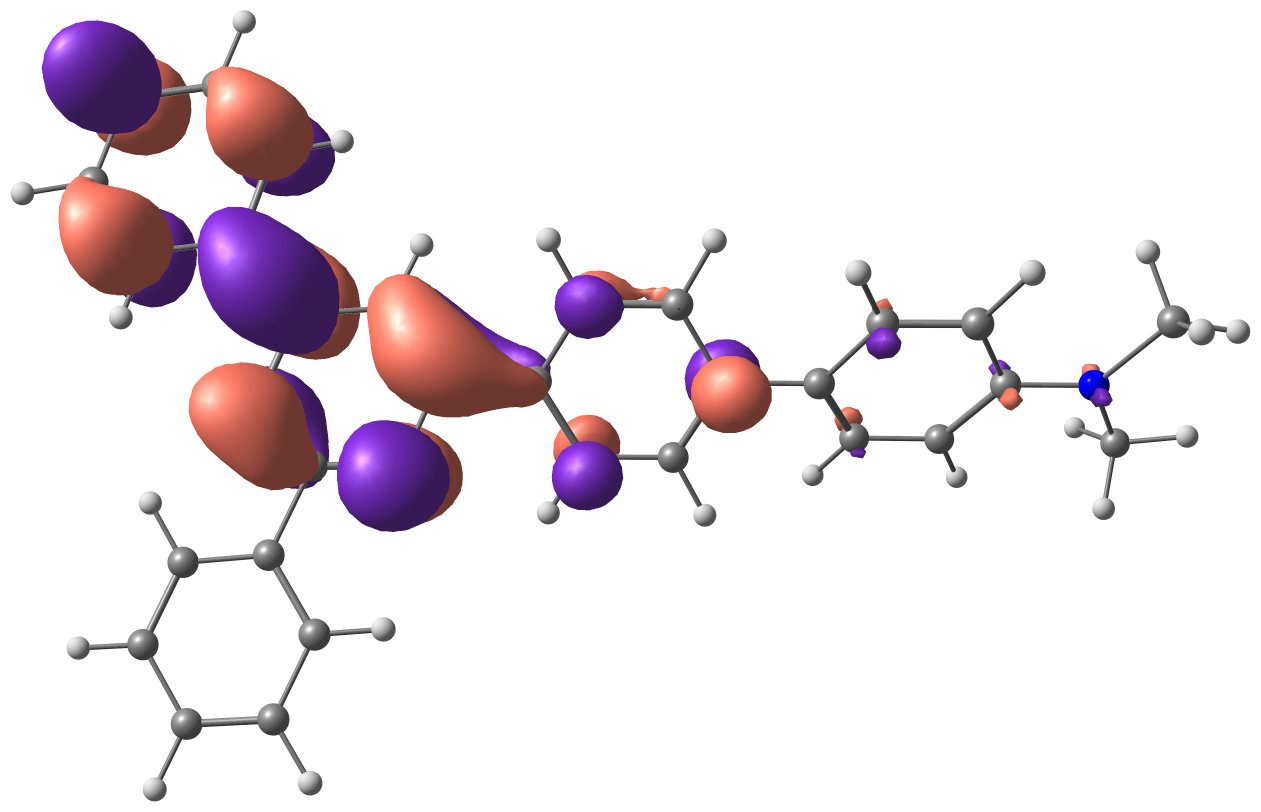 |
|  | S_0_-S_1_ (S_1,90_ geometry) | |
|  | 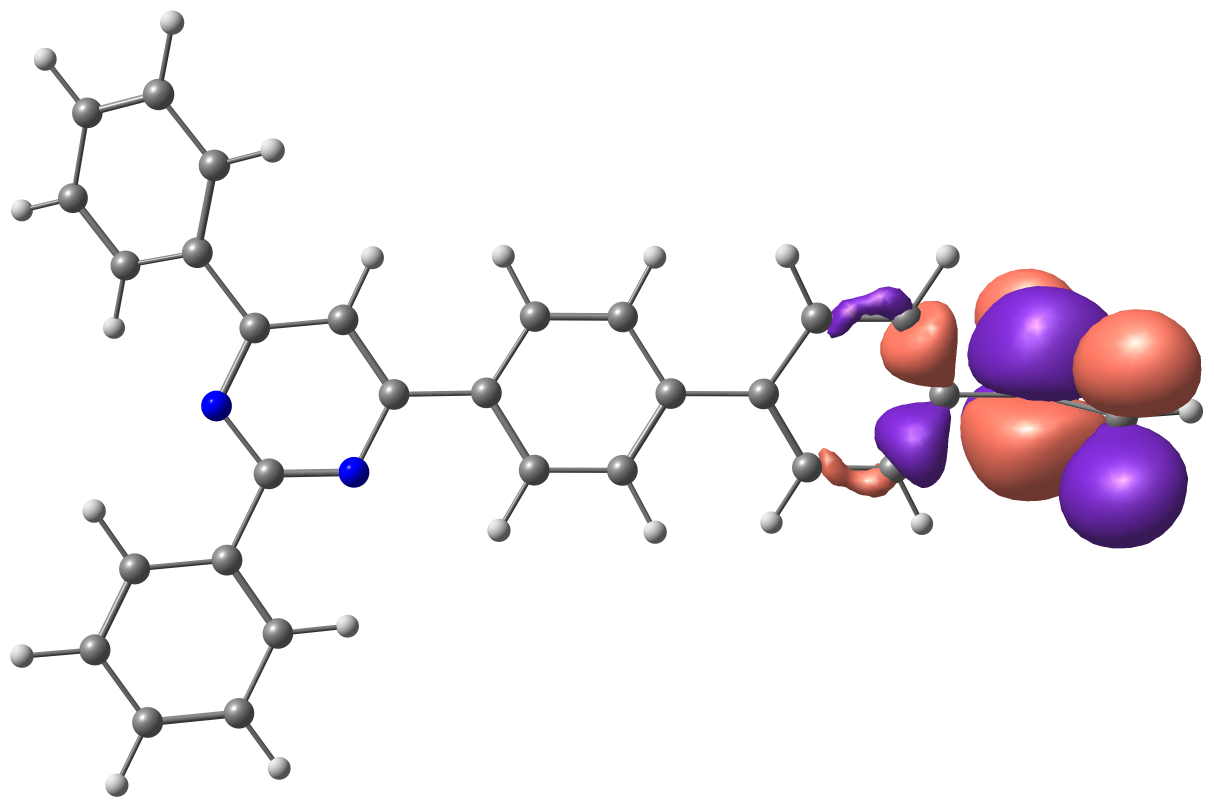 | 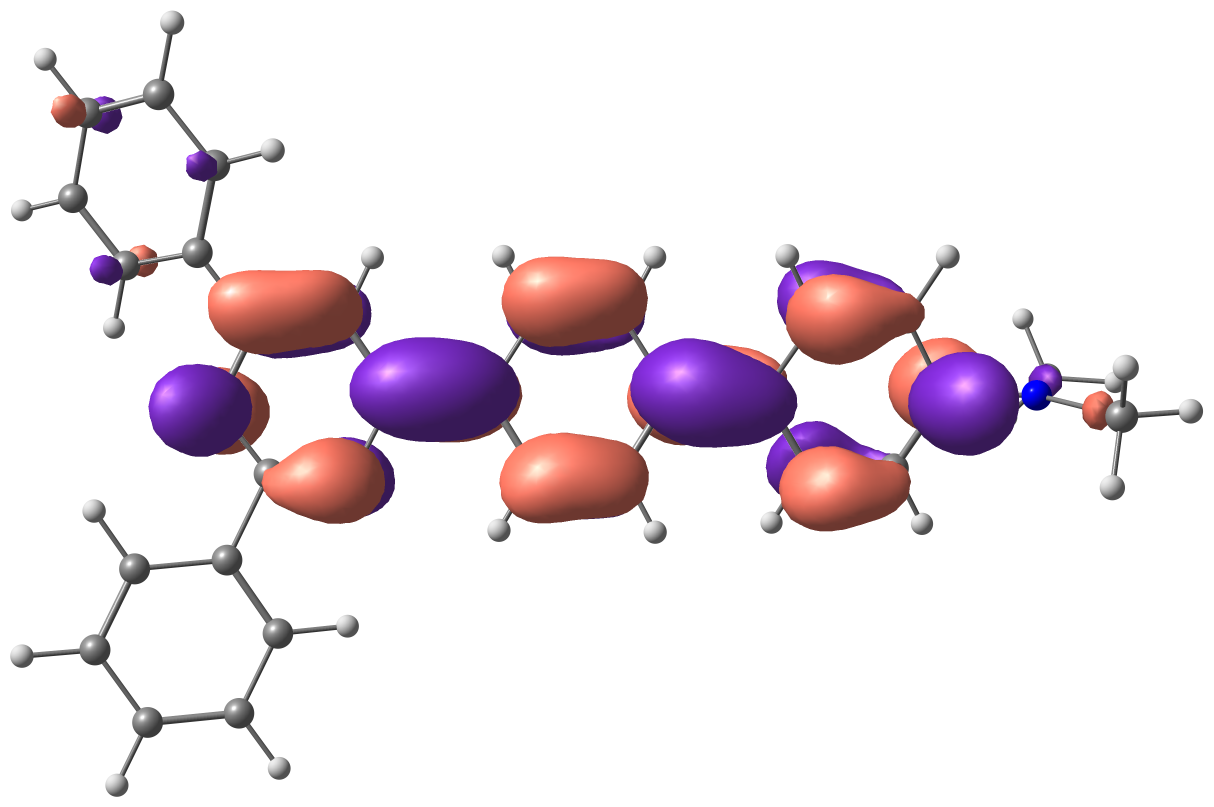 |
| DMSO | S_0_-S_1_ (S_1,0_ geometry) | |
|  | 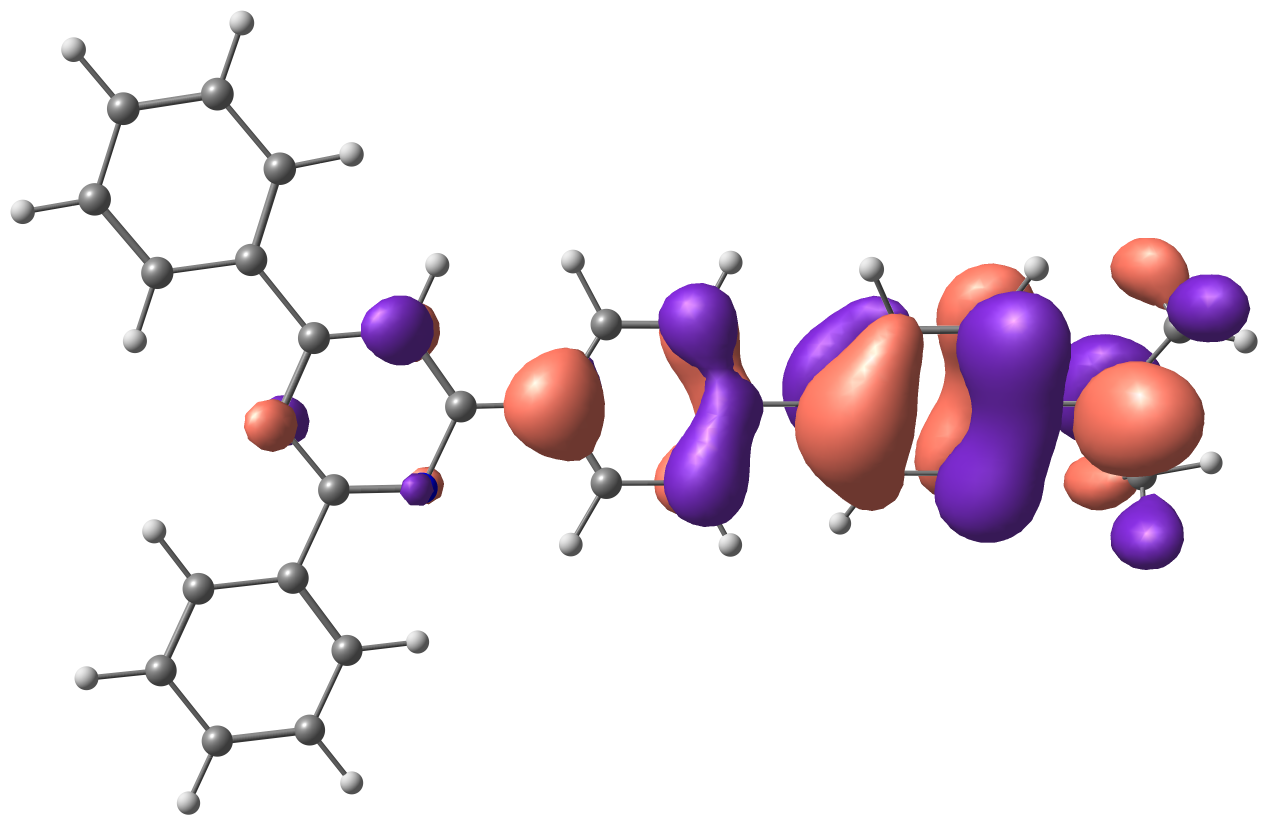 | 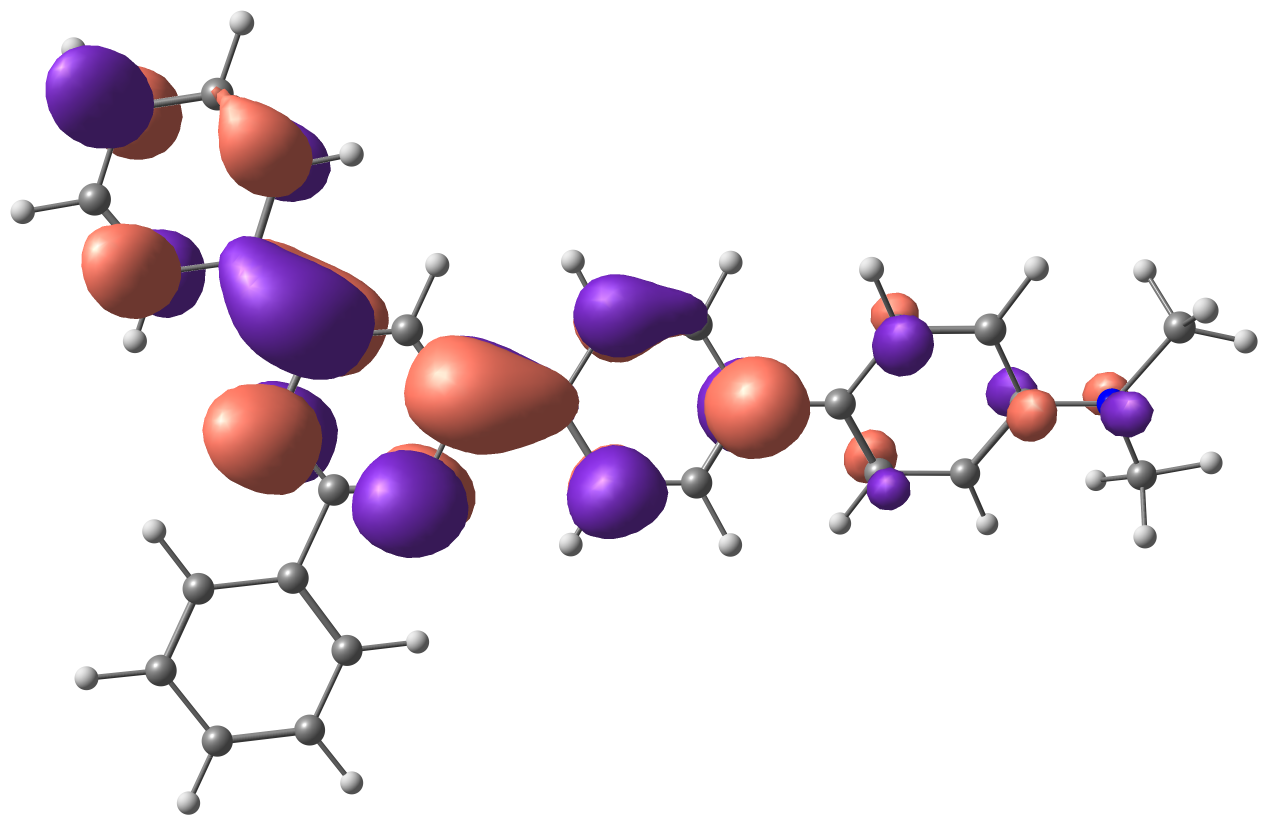 |
|  | S_0_-S_1_ (S_1,90_ geometry) | |
|  | 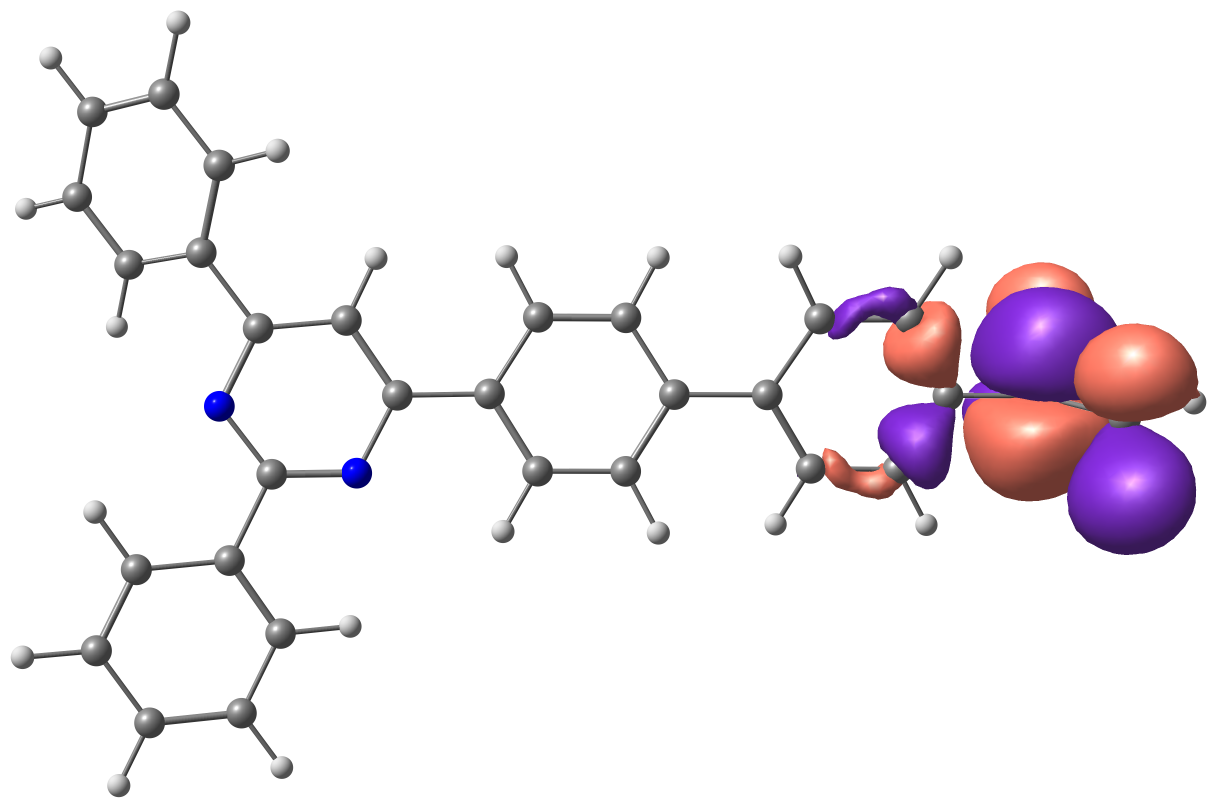 | 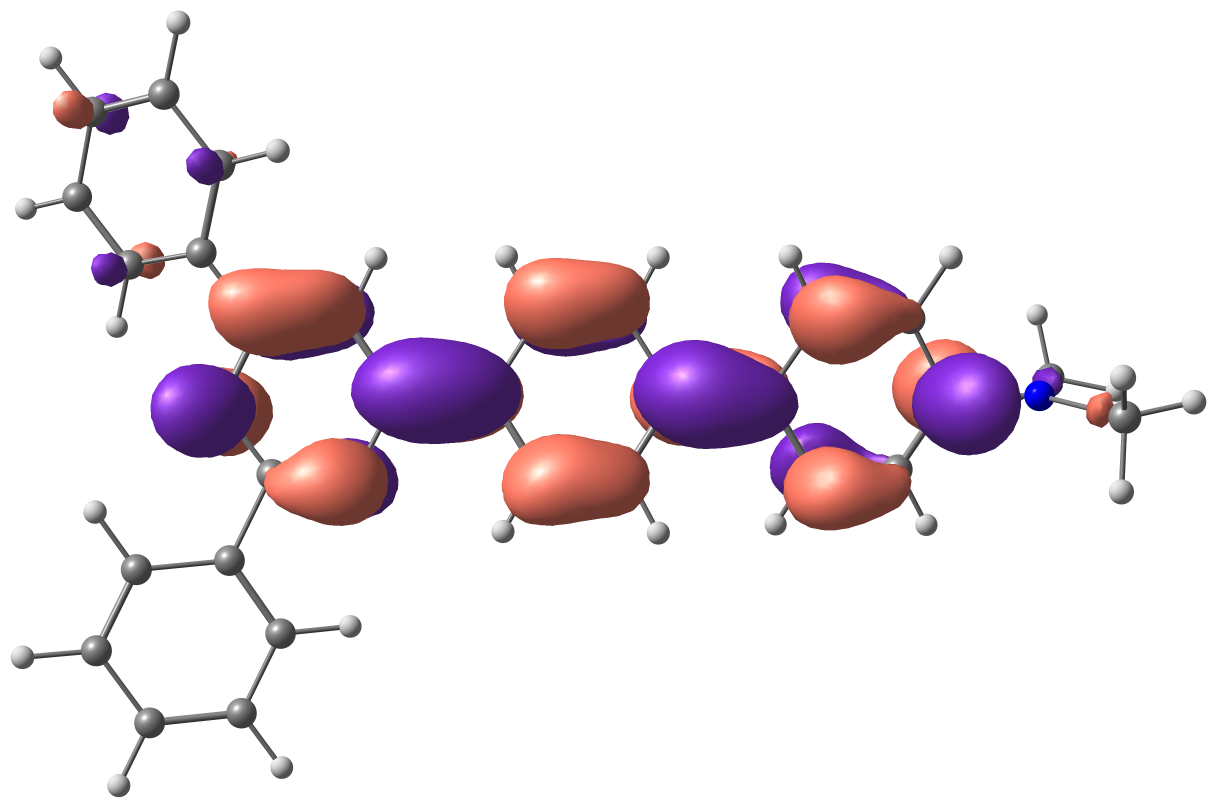 |

**Figure S8.** Natural transition orbital (NTO) pairs (isosurface contour plots: ±0.03 a.u.) describing the electronic transition to the S_1_ excited state at the S_1,0_ and S_1,90_ excited-state minimum-energy geometries for the **D1** molecule. Computed at the TD-DFT B3LYP/6-311G**(PCM) level of theory for Hx and DMSO solvents.


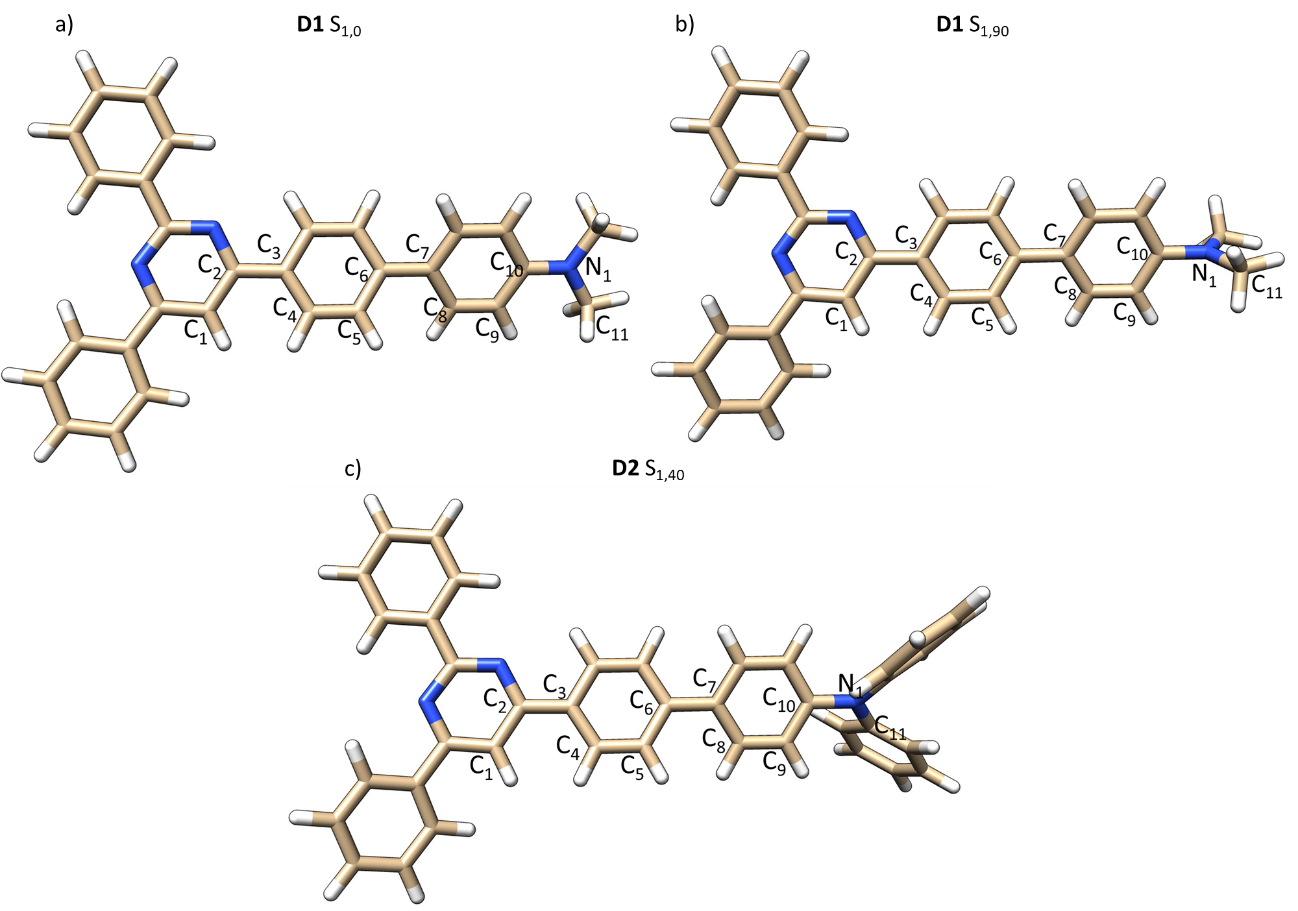


| **Solvent** | **Dihedral** | **D1** | | **D2** |
| --- | --- | --- | --- | --- |
|  |  | **S_1,0_** | **S_1,90_** | **S_1,40_** |
| **Hx** | C_1_C_2_C_3_C_4_ | 19.34 | -2.06 | -1.32 |
|  | C_5_C_6_C_7_C_8_ | 28.22 | 17.80 | 21.66 |
|  | C_9_C_10_N_1_C_11_ | -1.51 | 89.93 | 54.83 |
| **Tol** | C_1_C_2_C_3_C_4_ | 17.09 | -1.93 | -0.72 |
|  | C_5_C_6_C_7_C_8_ | 28.19 | 17.55 | 21.65 |
|  | C_9_C_10_N_1_C_11_ | -3.15 | 89.94 | 51.71 |
| **ACN** | C_1_C_2_C_3_C_4_ | -3.35 | -1.05 | -2.30 |
|  | C_5_C_6_C_7_C_8_ | 24.22 | 16.11 | 16.62 |
|  | C_9_C_10_N_1_C_11_ | -0.16 | 90.84 | 40.20 |
| **DMSO** | C_1_C_2_C_3_C_4_ | -3.22 | -1.01 | -2.30 |
|  | C_5_C_6_C_7_C_8_ | 24.09 | 16.09 | 16.50 |
|  | C_9_C_10_N_1_C_11_ | -0.17 | 90.83 | 40.07 |

**Figure S9.** TD-DFT/B3LYP/6-311G**-optimized geometries calculated for the excited state (S_1_) of **D1** (a and b) and **D2** (c). The values of the dihedral angles defining the optimized structures are given in the table expressed in degrees. Solvents effects (Hx, Tol, ACN and DMSO) were considered using the PCM method.

| 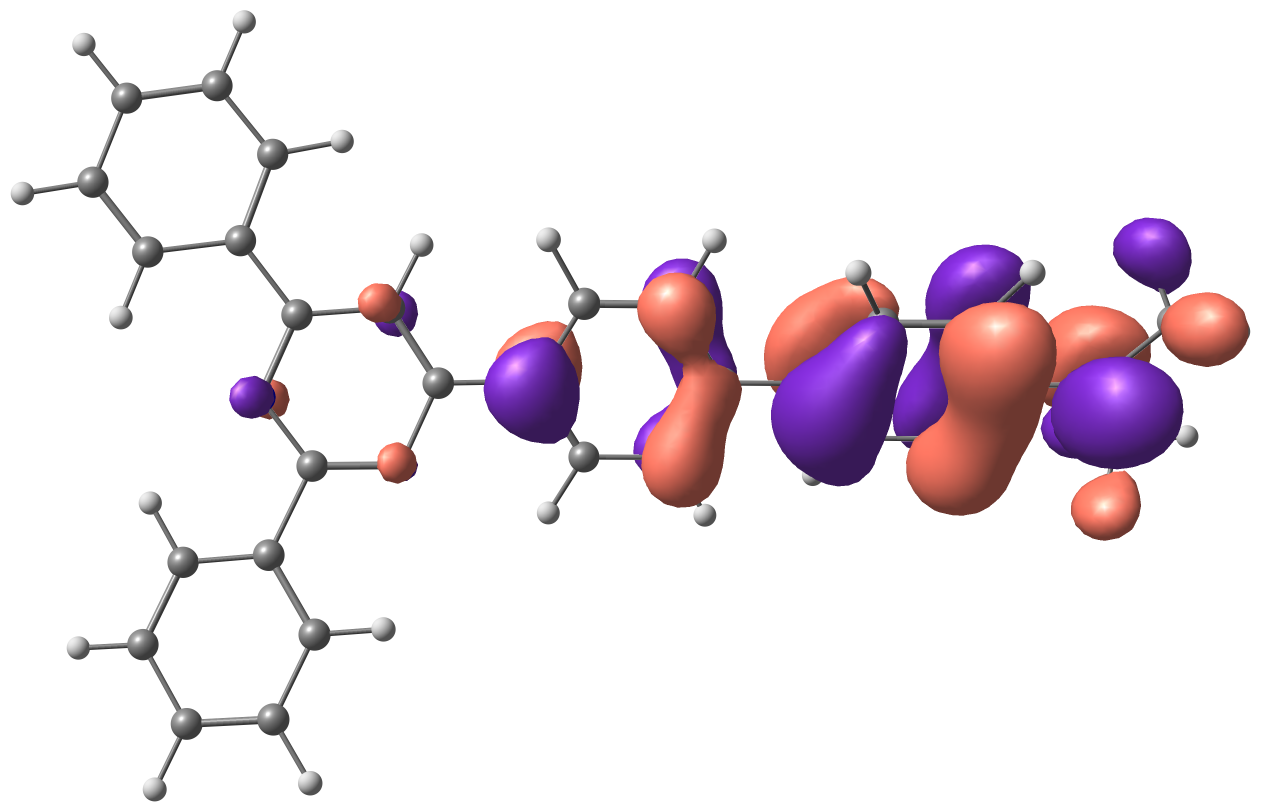  S_1,0_ HOMO (Hx) | 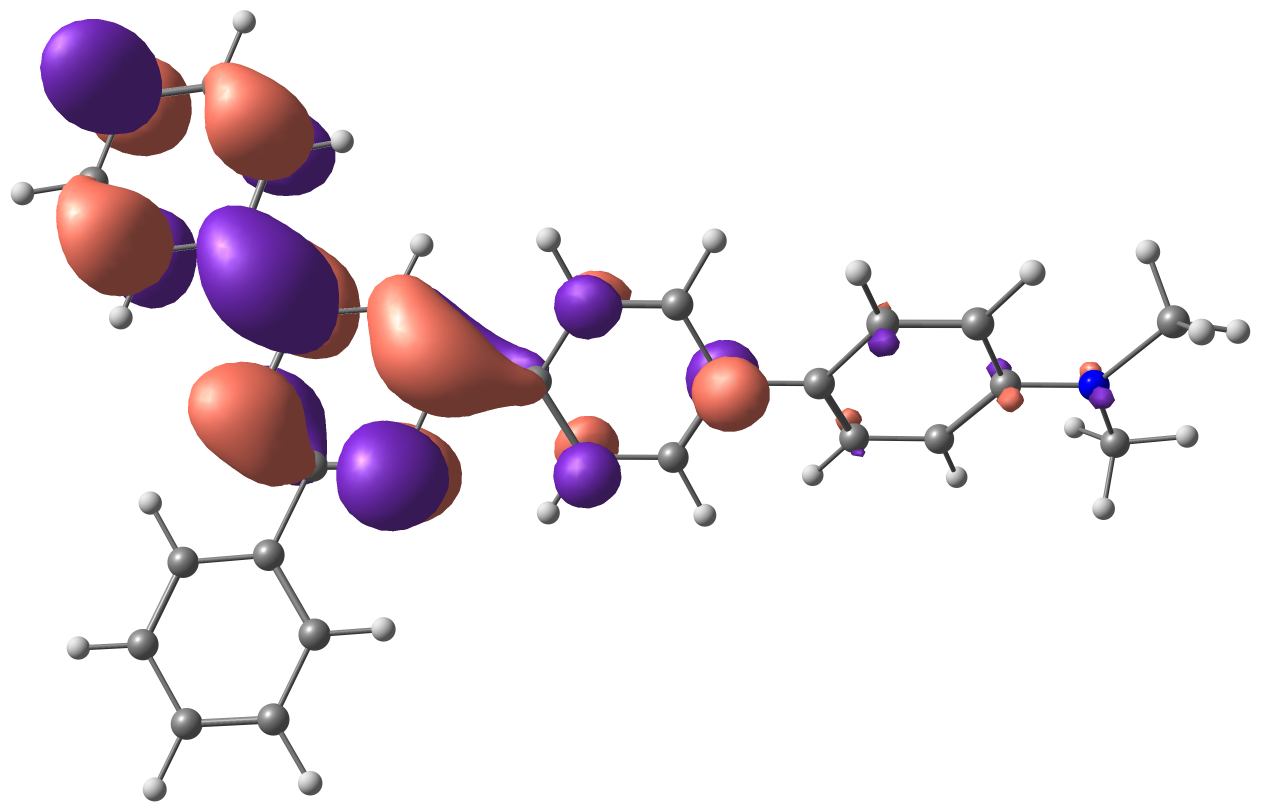  S_1,0_ LUMO (Hx) | 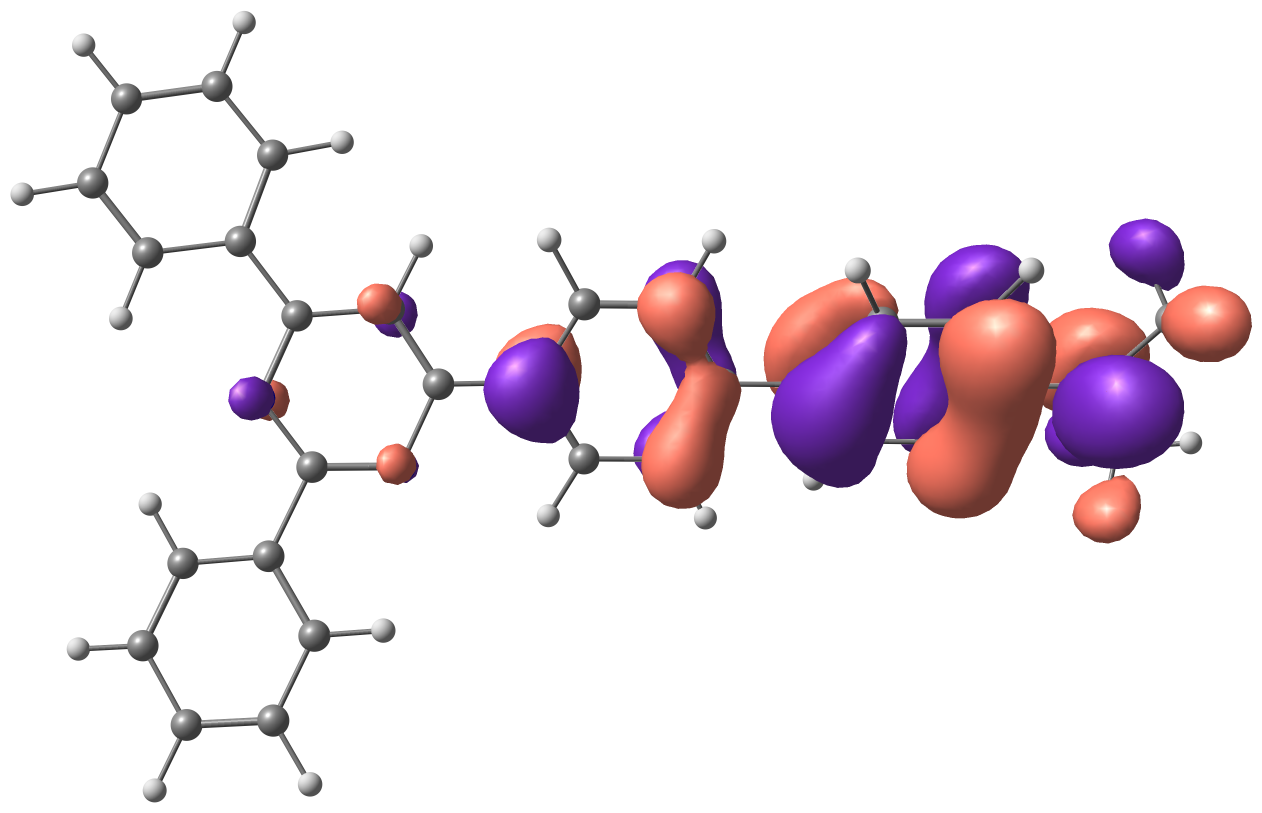  S_1,0_ HOMO (Tol) | 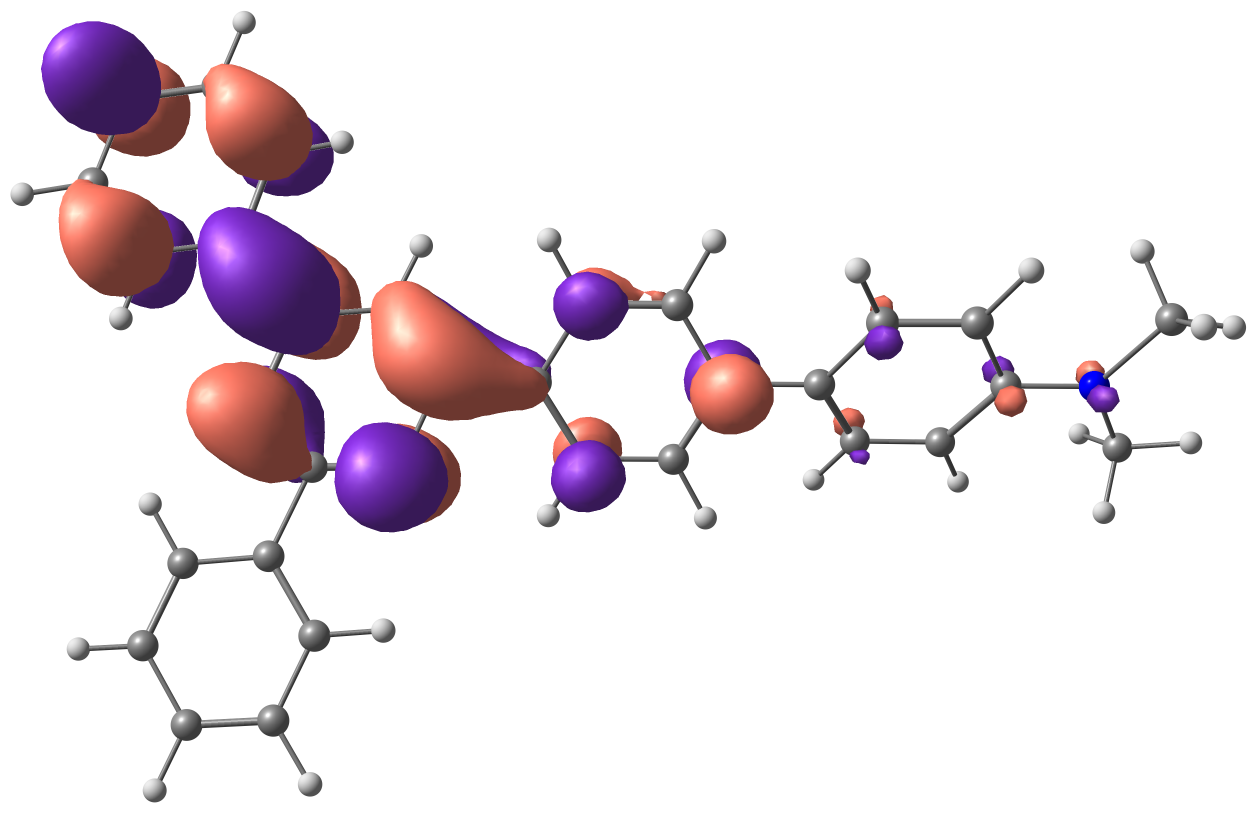  S_1,0_ LUMO (Tol) |
| --- | --- | --- | --- |
| 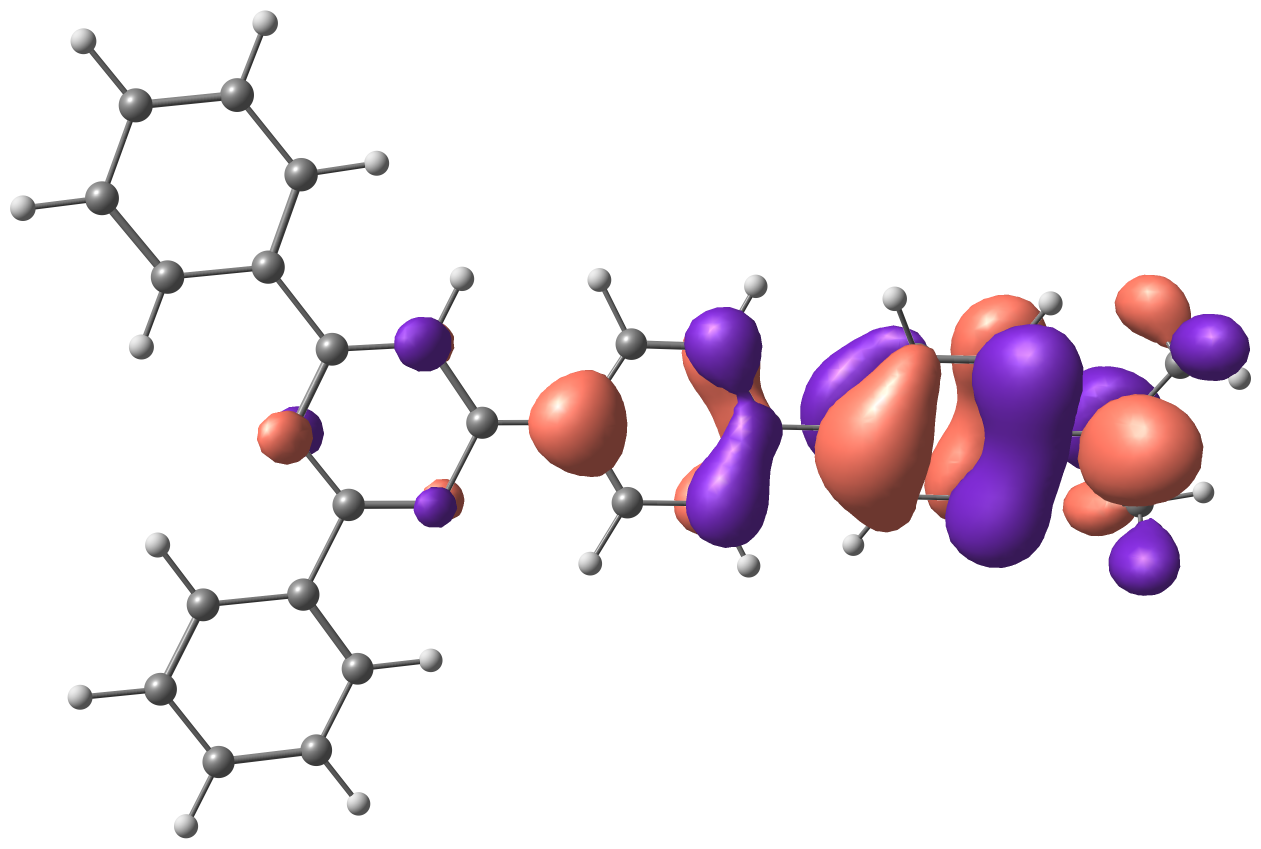 S_1,0_ HOMO (ACN) | 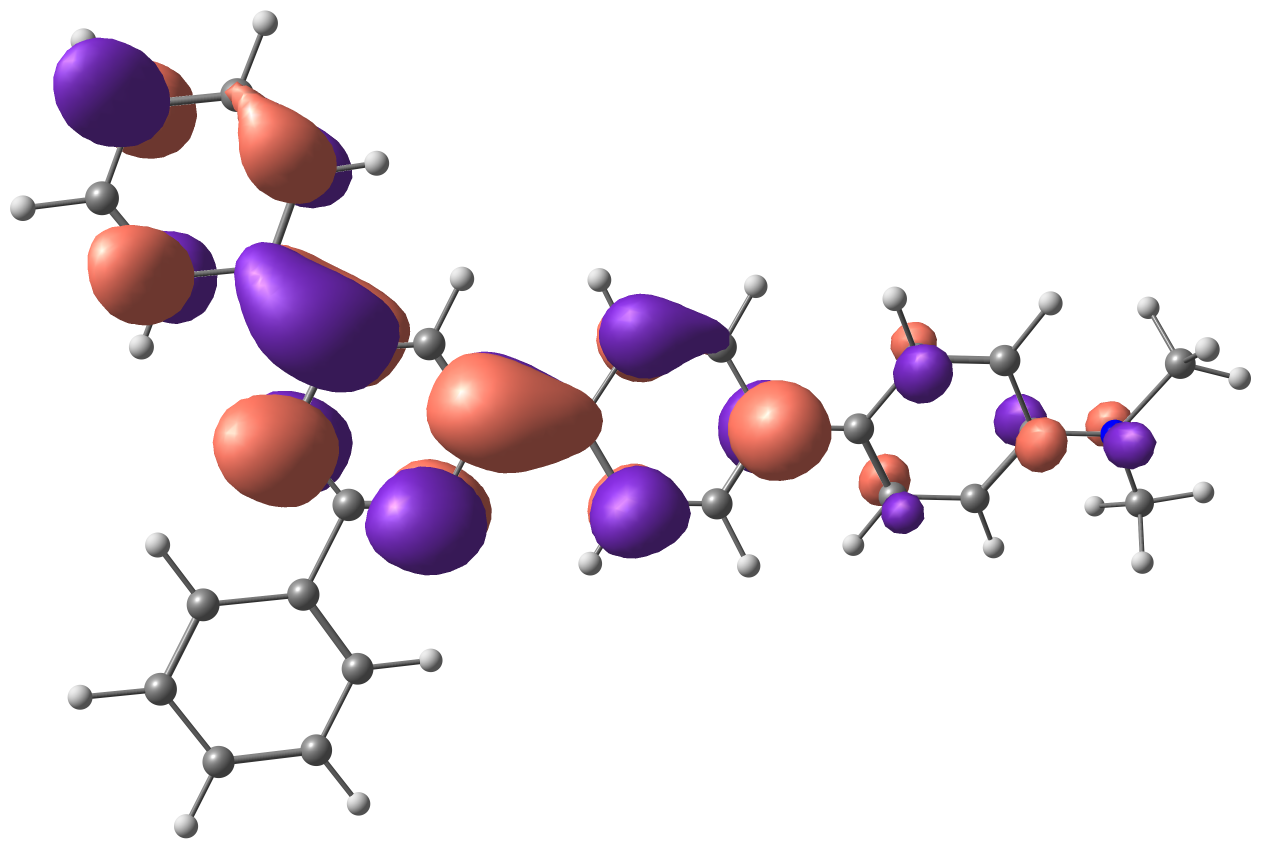 S_1,0_ LUMO (ACN) | 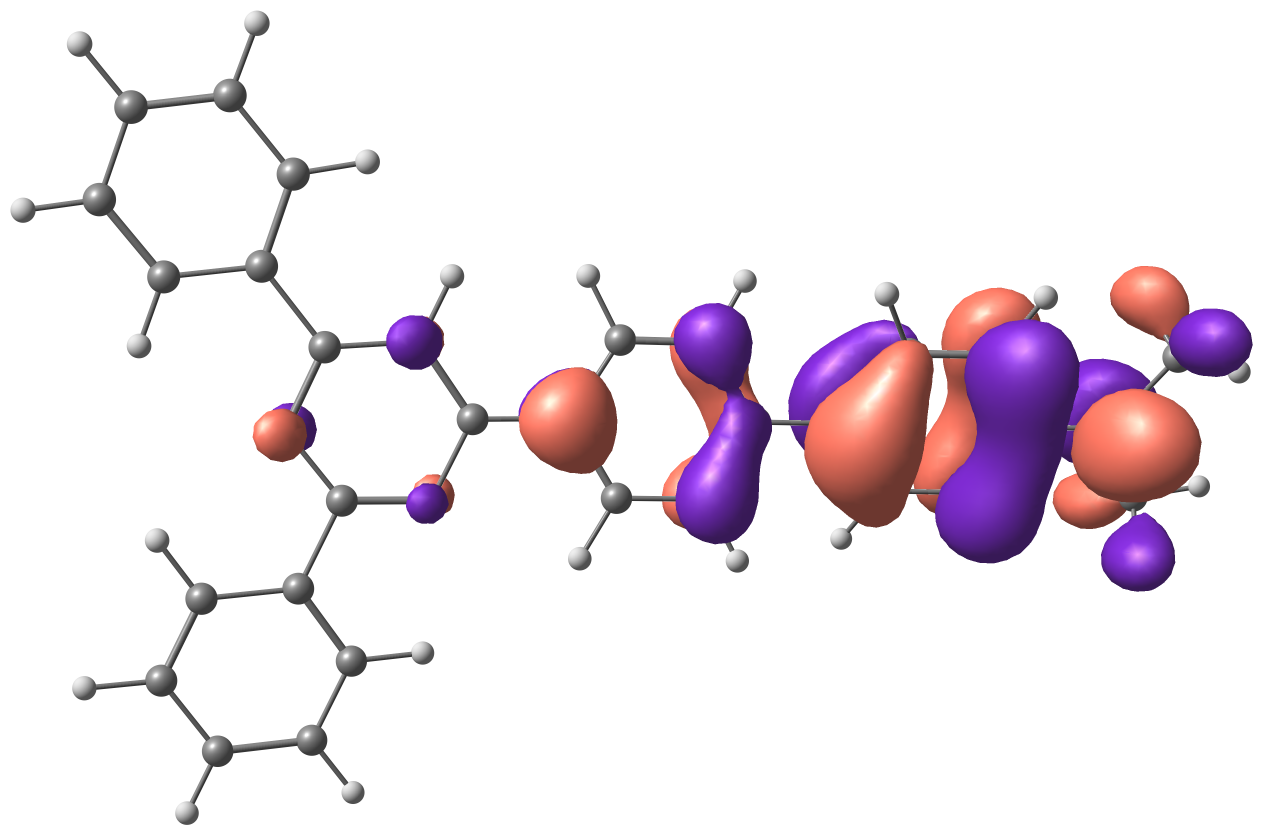  S_1,0_ HOMO (DMSO) | 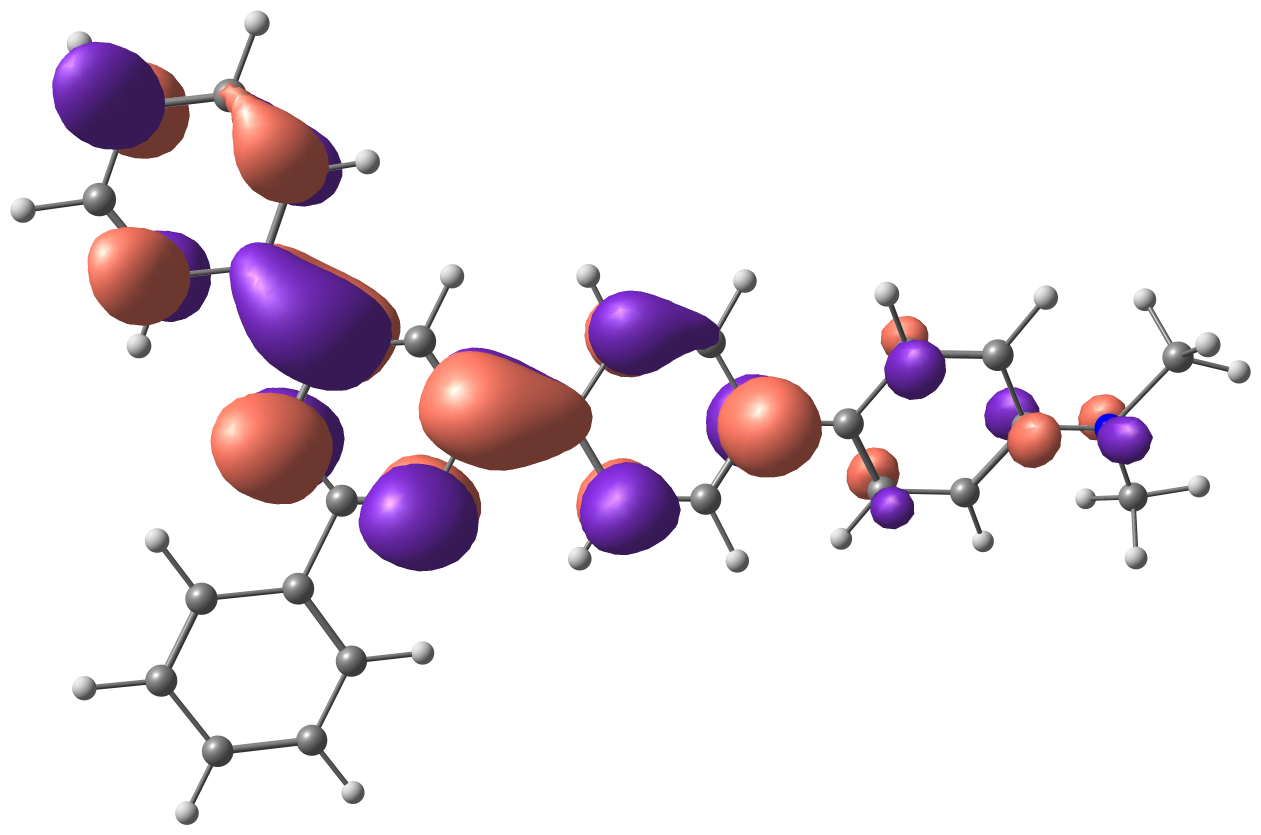  S_1,0_ LUMO (DMSO) |
| 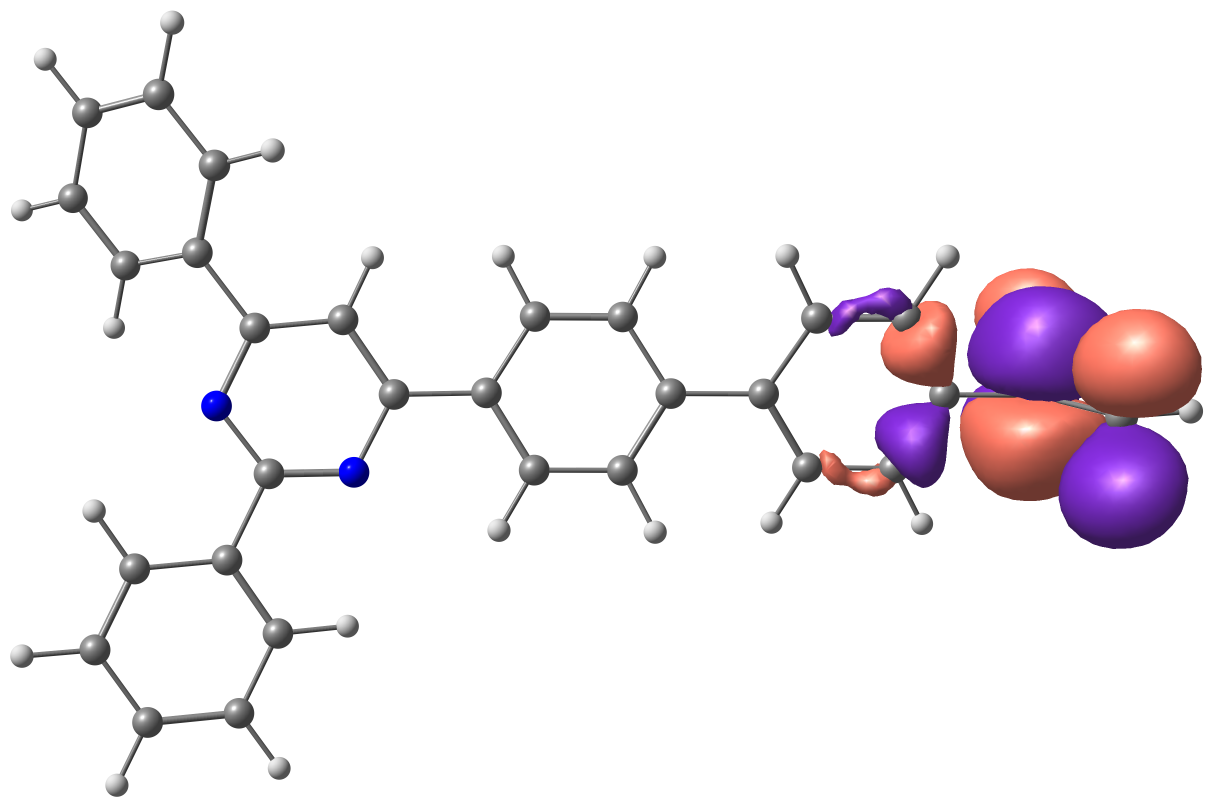  S_1,90_ HOMO (Hx) | 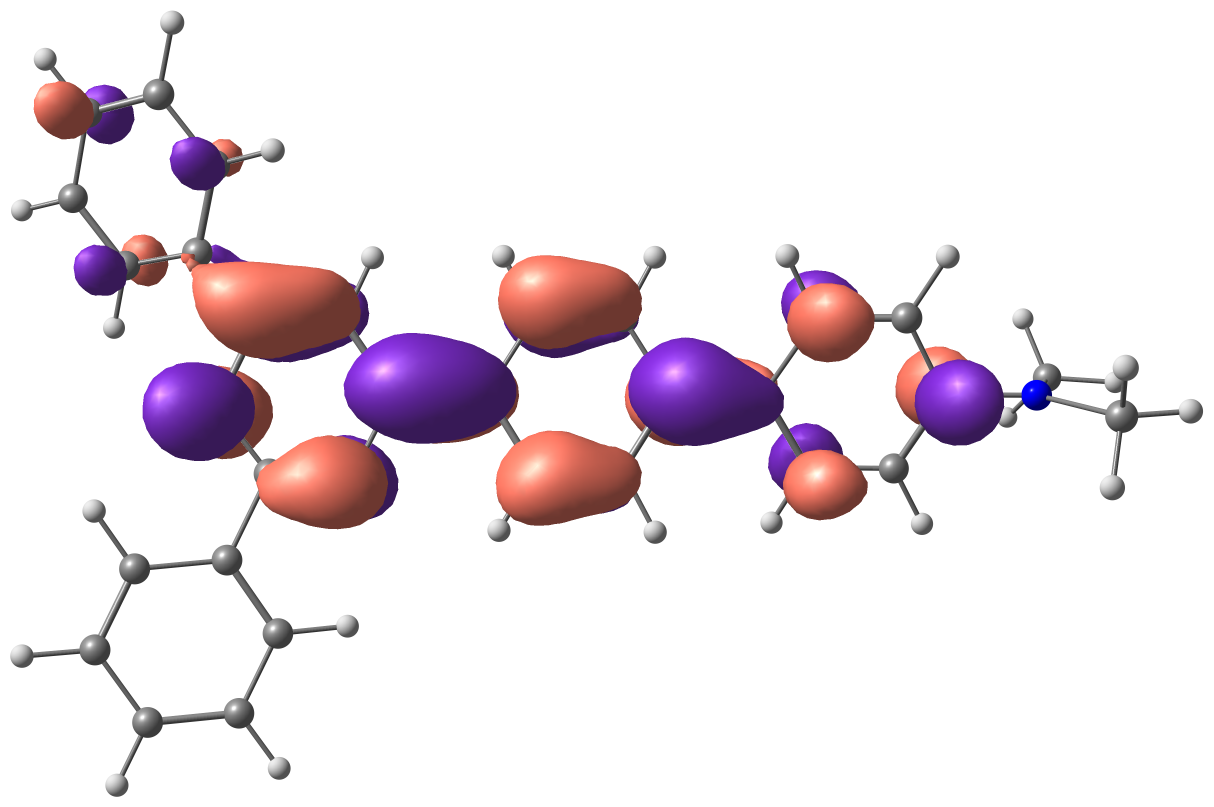  S_1,90_ LUMO (Hx) | 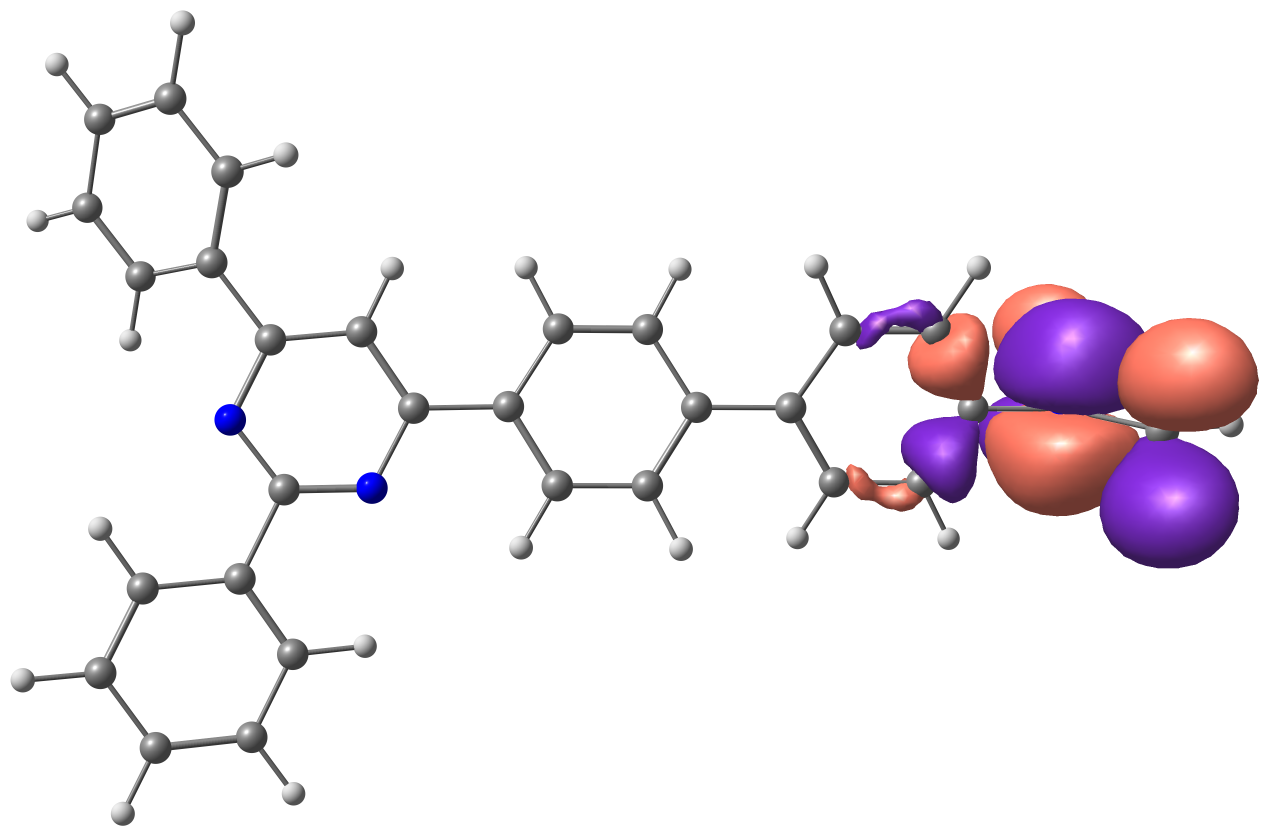  S_1,90_ HOMO (Tol) | 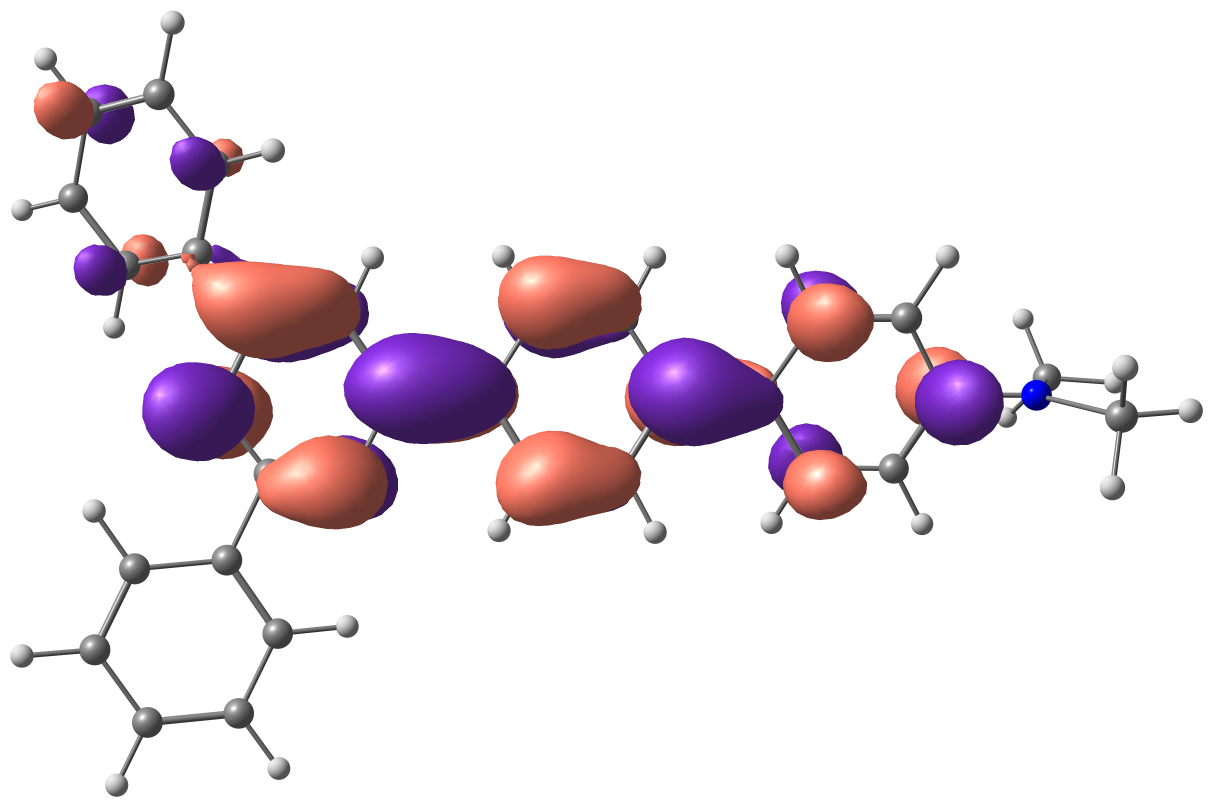  S_1,90_ LUMO (Tol) |
| 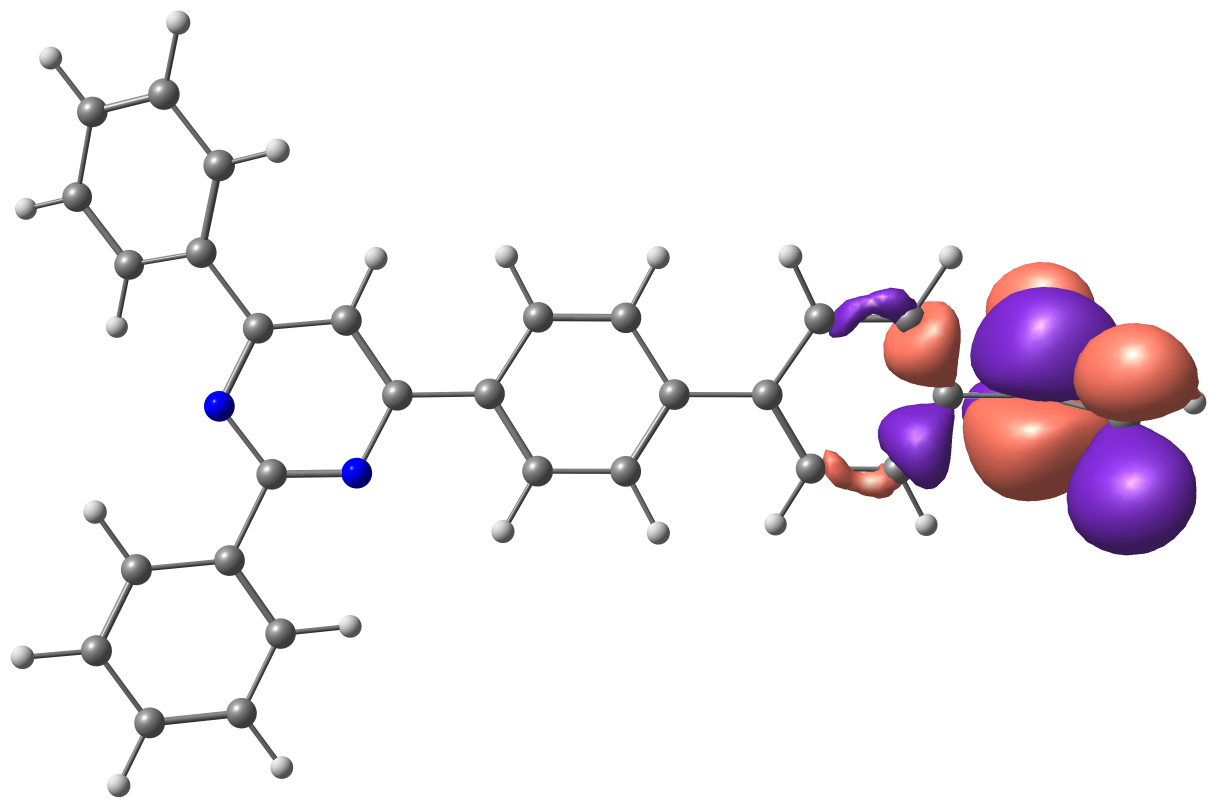 S_1,90_ HOMO (ACN) | 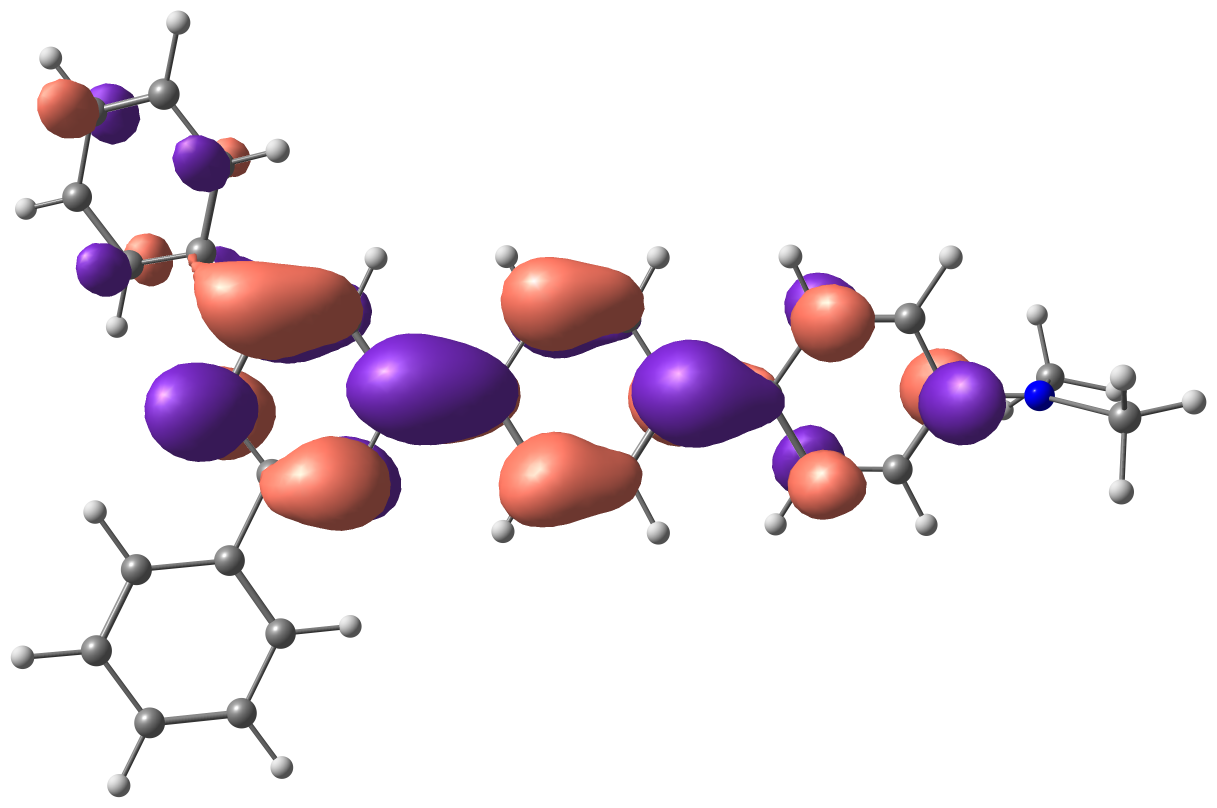 S_1,90_ LUMO (ACN) | 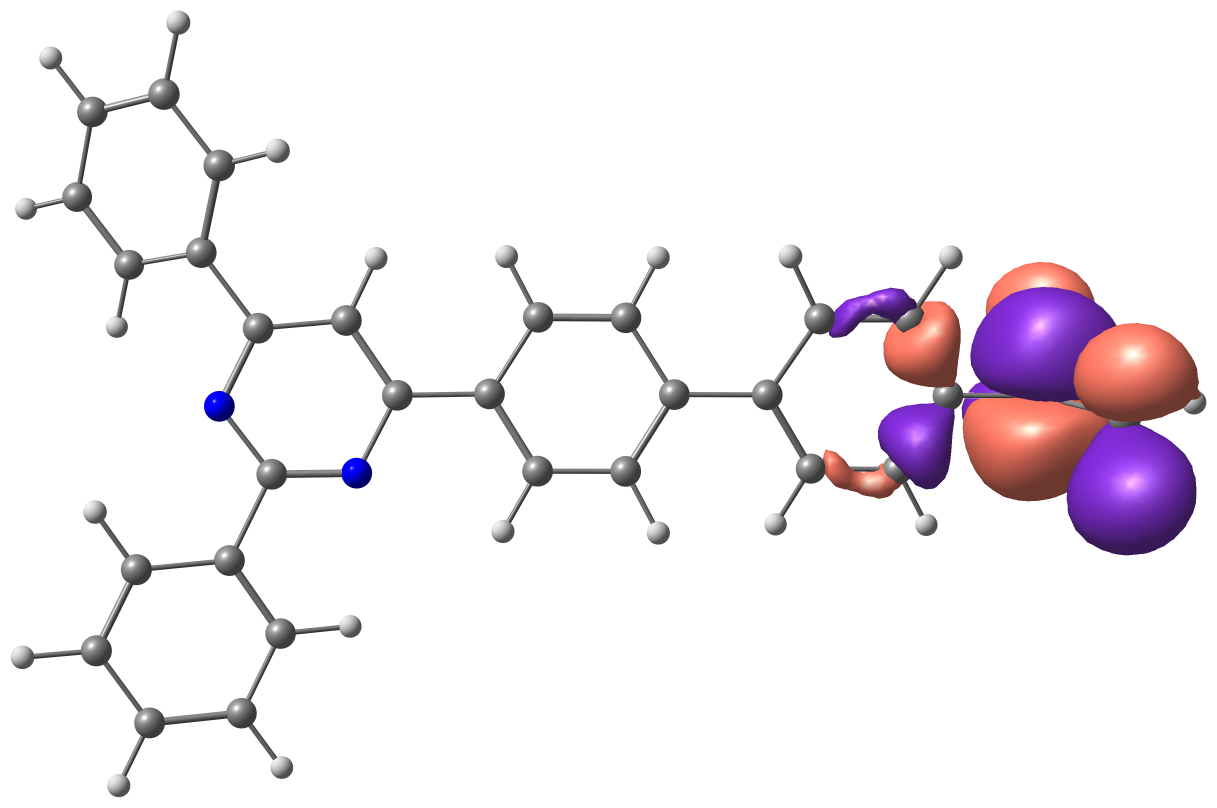  S_1,90_ HOMO (DMSO) | 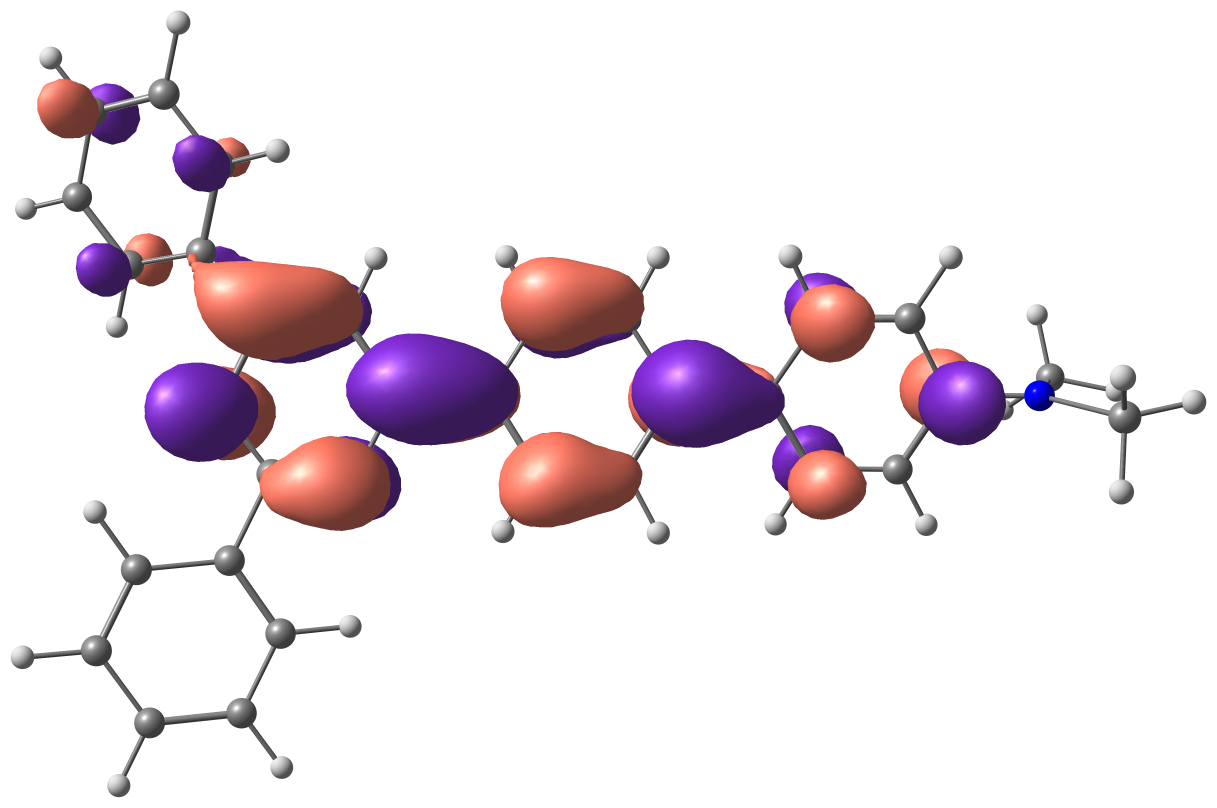  S_1,90_ LUMO (DMSO) |

**Figure S10**. Isosurface contour plots (±0.03 a.u.) calculated at the B3LYP/6-311G**(PCM) level for the frontier MOs of **D1** at the S_1,0_ and S_1,90_ minimum-energy geometries in Hx, Tol, ACN and DMSO.

| 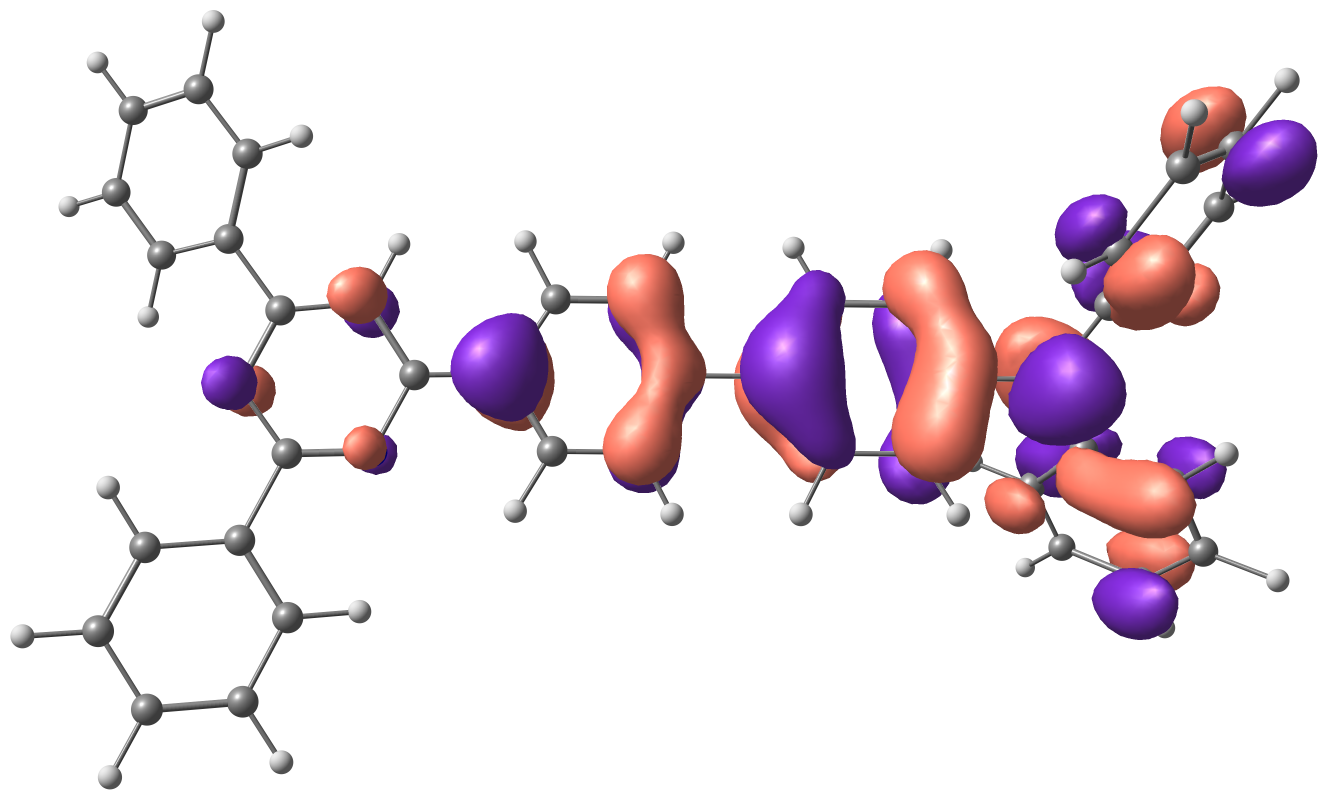  S_1,40_ HOMO (Hx) | 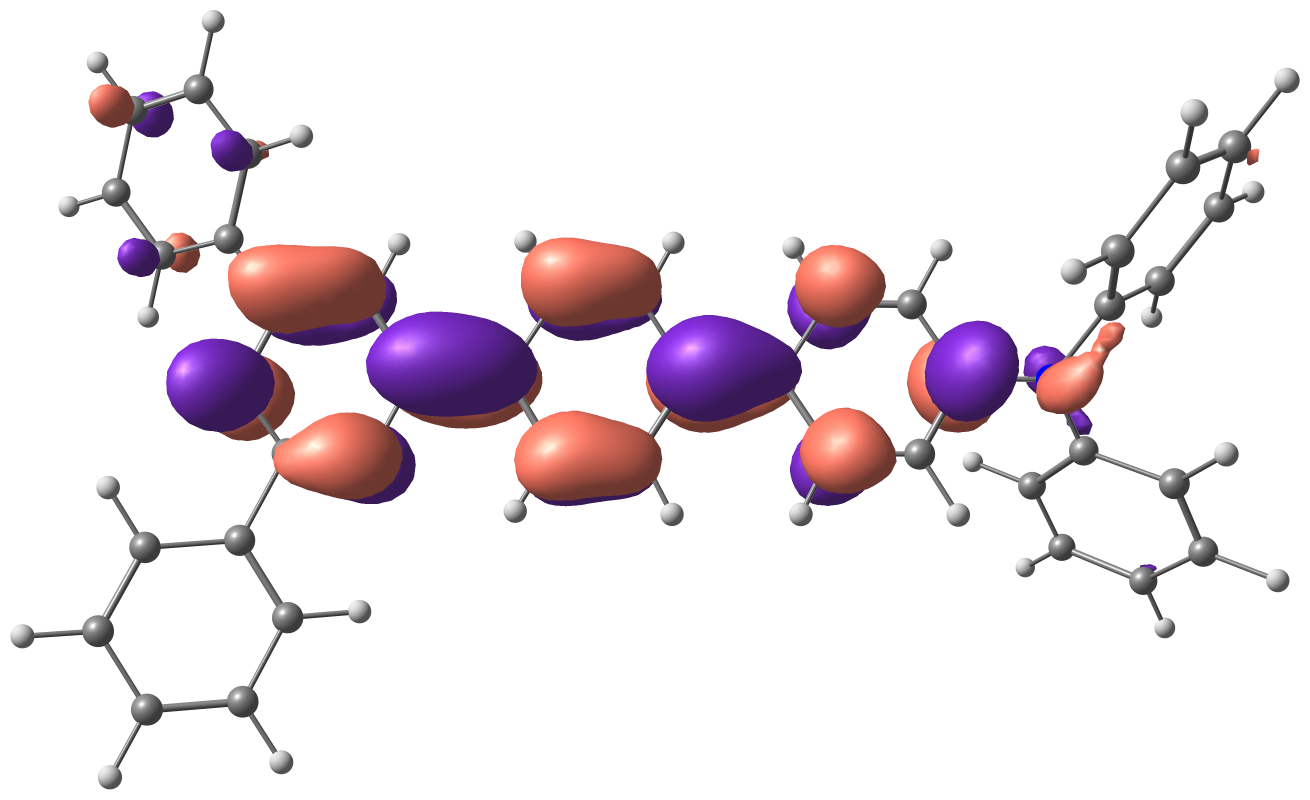  S_1,40_ LUMO (Hx) | 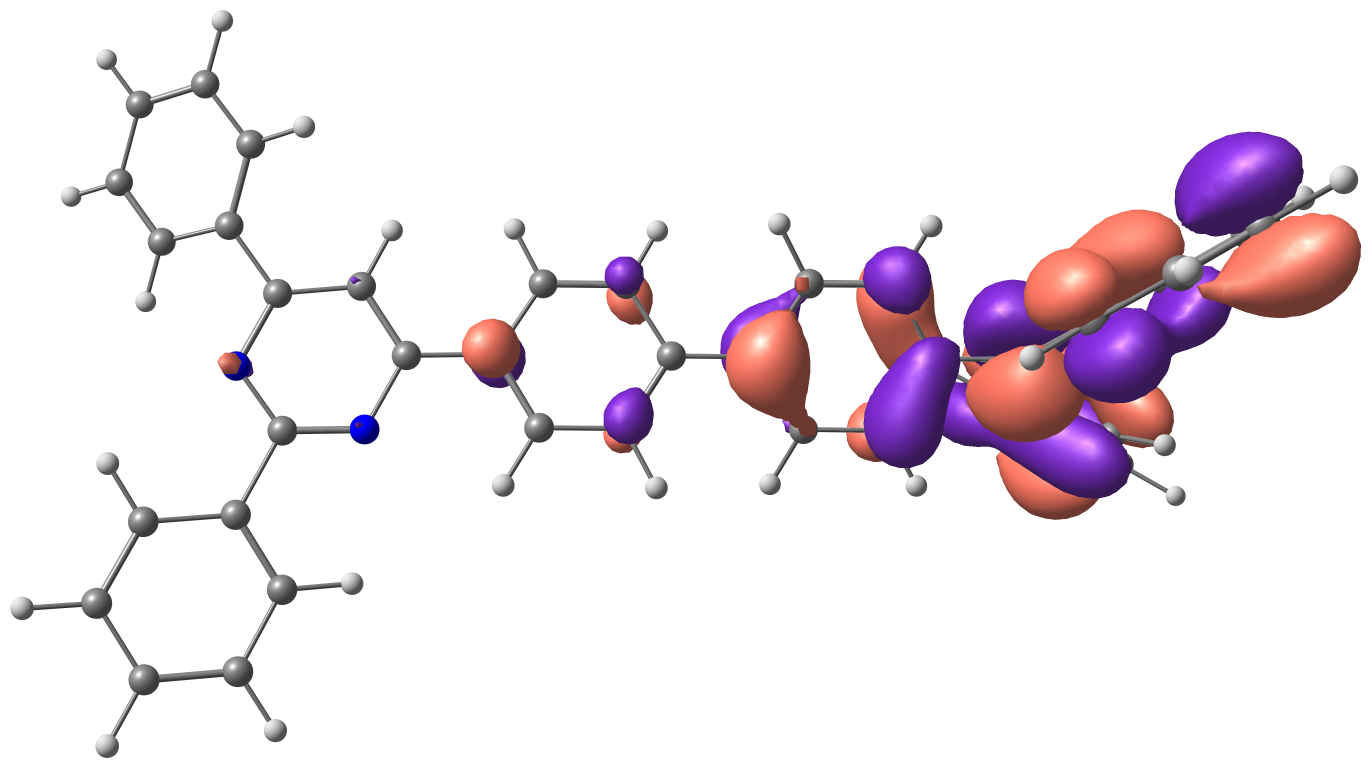  S_1,40_ HOMO (Tol) | 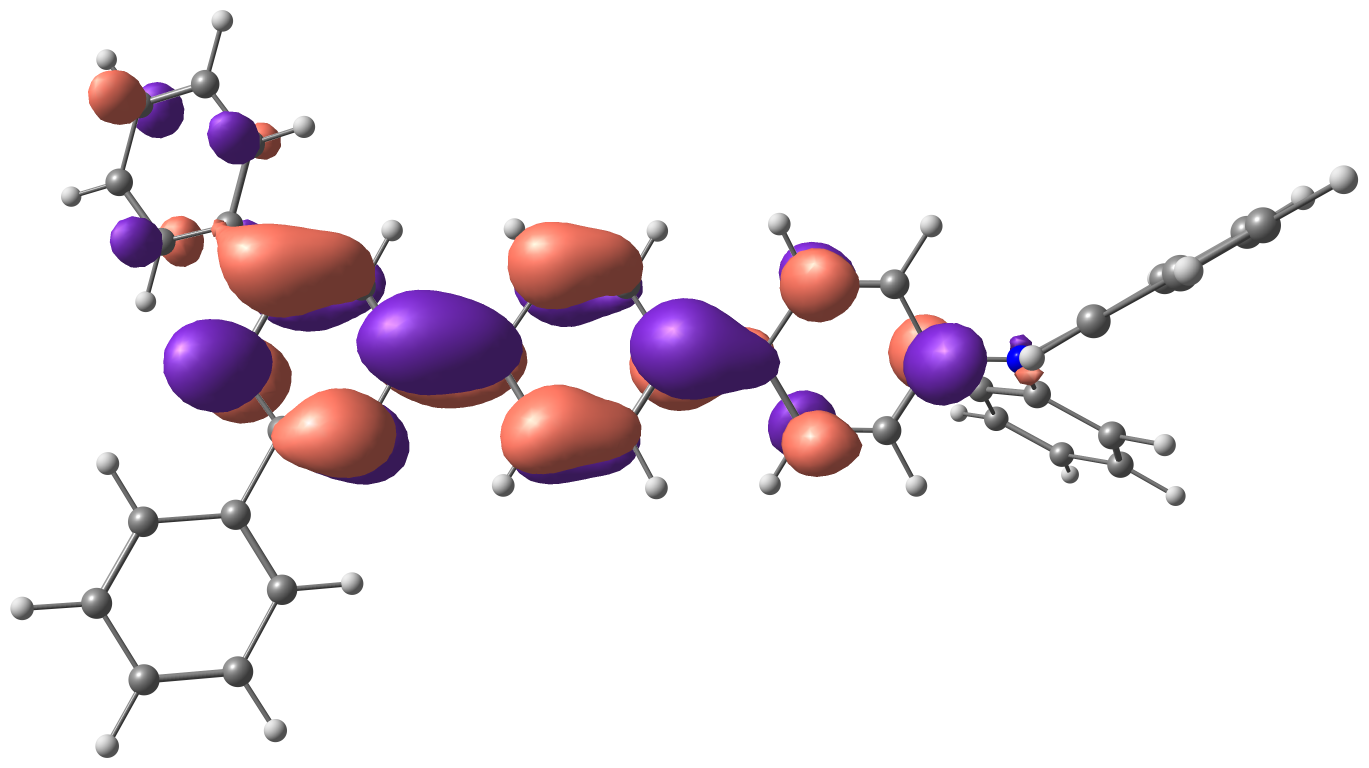  S_1,40_ LUMO (Tol) |
| --- | --- | --- | --- |
| 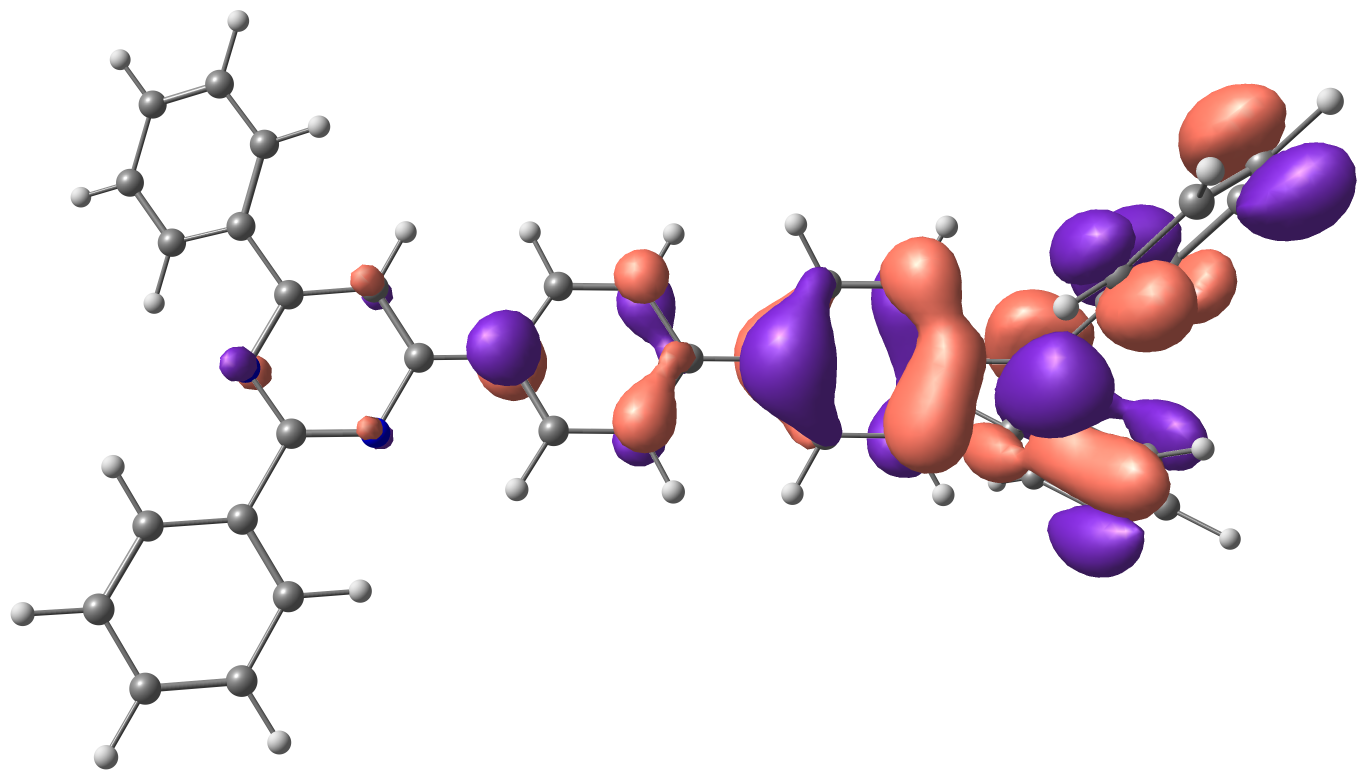 S_1,40_ HOMO (ACN) | 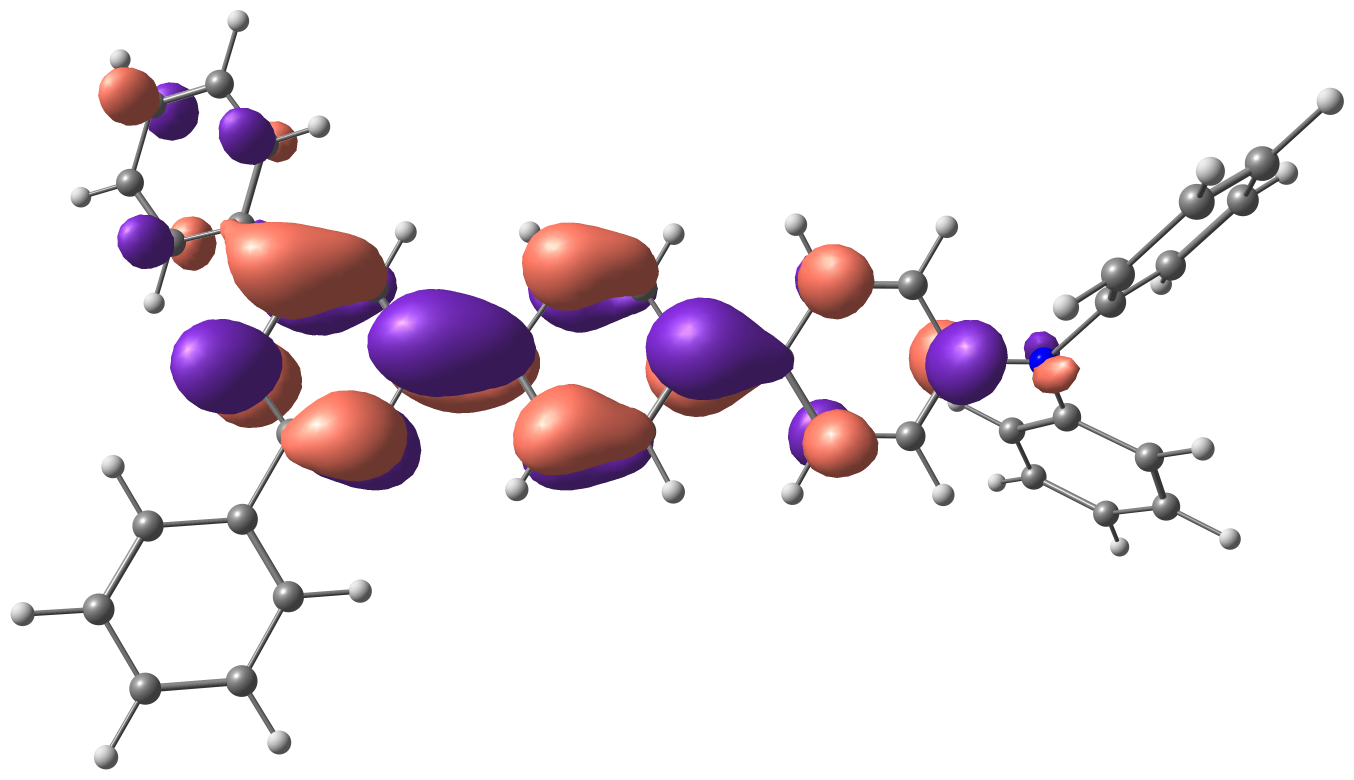 S_1,40_ LUMO (ACN) | 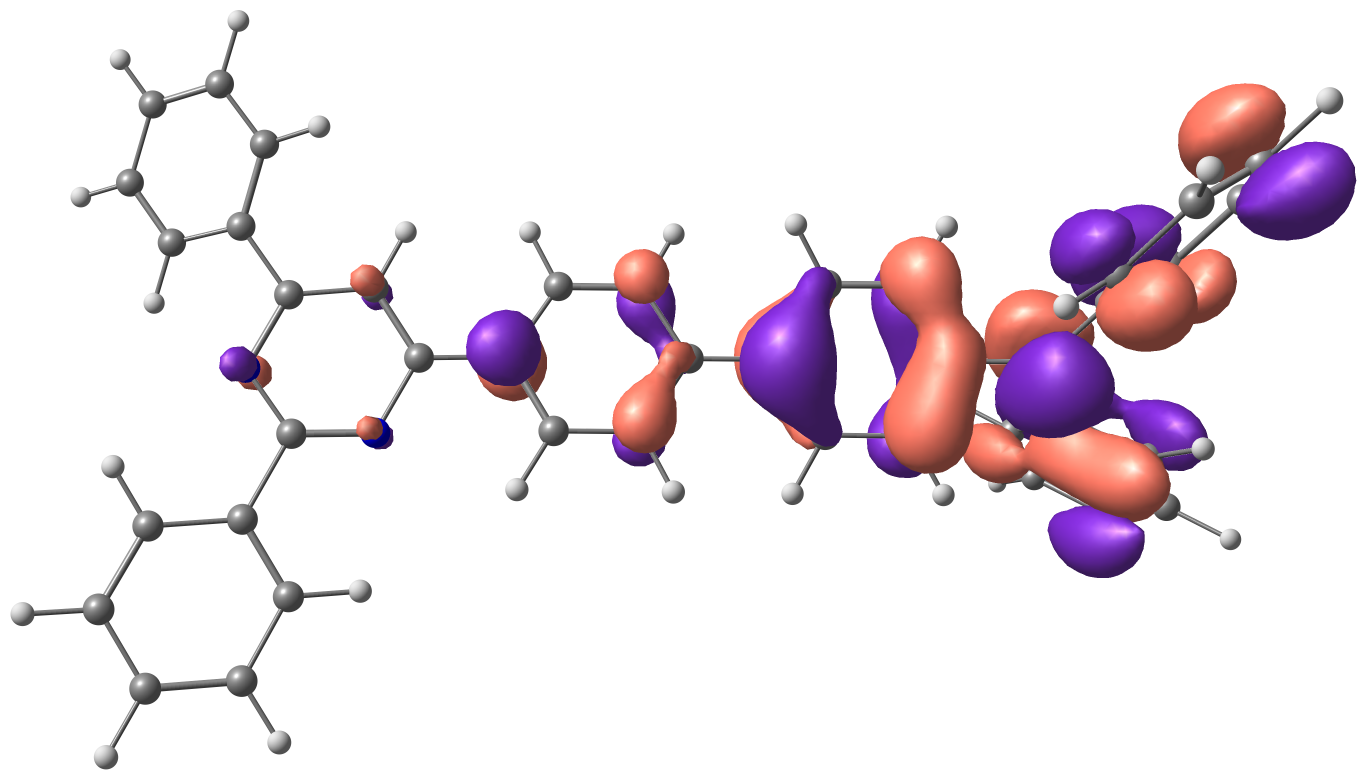  S_1,40_ HOMO (DMSO) | 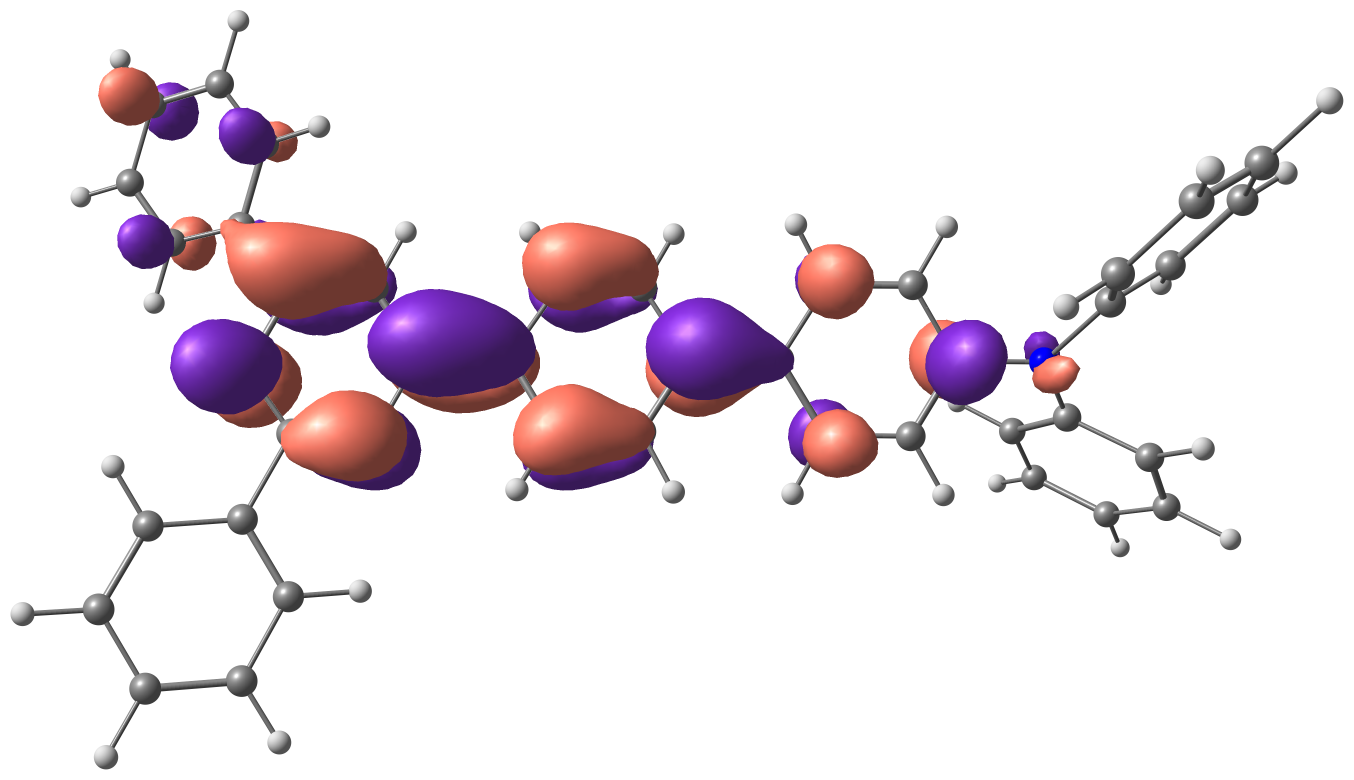  S_1,40_ LUMO (DMSO) |

**Figure S11**. Isosurface contour plots (±0.03 a.u.) calculated at the B3LYP/6-311G**(PCM) level for the frontier MOs of **D2** at the S_1,40_ minimum-energy geometry in Hx, Tol, ACN and DMSO).

**
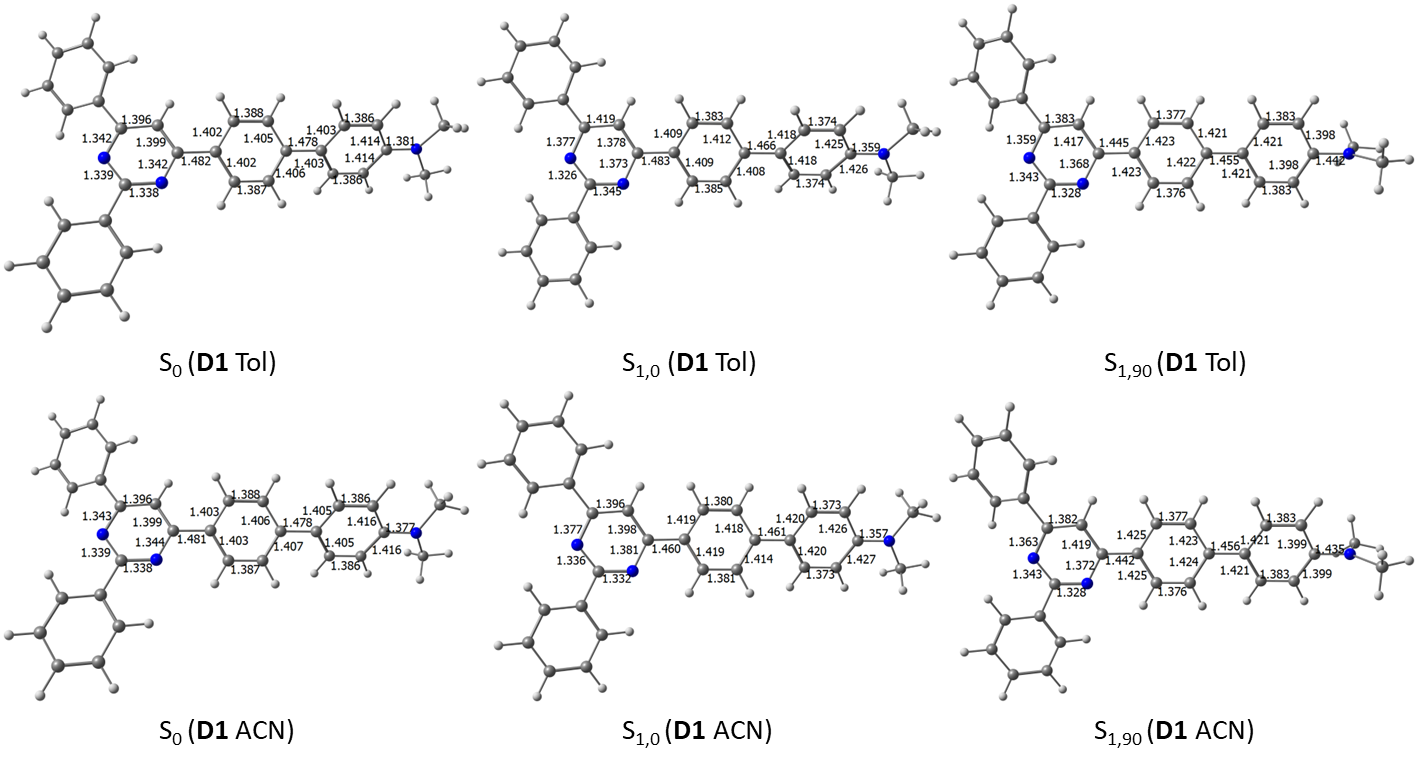

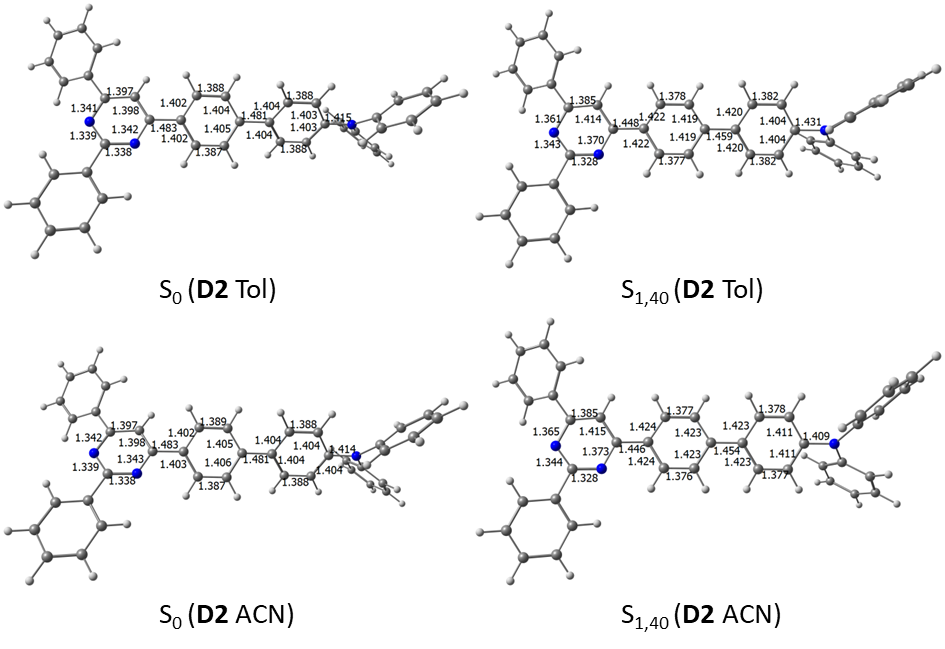
**

**Figure S12.** Optimized bond distances calculated for the S_0_ and S_1_ minima of **D1** and **D2** at the DFT (TD-DFT) B3LYP/6-311G** level of theory, including solvent effects (Tol and ACN) using the PCM method. All distances expressed in Å.

**Table S6:** S_0_ and S_1_ relative energies (eV) calculated for **D1** at the optimized S_0_, S_1,0_ and S_1,90_ minima in four different solvents (Hx, Tol, ACN, and DMSO). Energies are computed at the TD-DFT B3LYP/6-311G** level of theory using PCM for simulating the solvent and the corrected linear response method.

| **Solvent** | **Geometry** | **S_0_** | **S_1_** |
| --- | --- | --- | --- |
| **Hx** | **S_0_** | 0.00 | 2.77 |
|  | **S_1,0_** | 0.19 | 2.48 |
|  | **S_1,90_** | 0.76 | 2.70 |
| **Tol** | **S_0_** | 0.00 | 2.68 |
|  | **S_1,0_** | 0.20 | 2.39 |
|  | **S_1,90_** | 0.79 | 2.55 |
| **ACN** | **S_0_** | 0.00 | 2.57 |
|  | **S_1,0_** | 0.43 | 2.13 |
|  | **S_1,90_** | 1.41 | 1.81 |
| **DMSO** | **S_0_** | 0.00 | 2.52 |
|  | **S_1,0_** | 0.41 | 2.12 |
|  | **S_1,90_** | 1.37 | 1.79 |

**Table S7:** S_0_ and S_1_ relative energies (eV) calculated for **D2** at the optimized S_0_ and S_1,40_ minima in four different solvents (Hx, Tol, ACN and DMSO). Energies are computed at the TD-DFT B3LYP/6-311G** level of theory using PCM for simulating the solvent and the corrected linear response method.

| **Solvent** | **Geometry** | **S_0_** | **S_1_** |
| --- | --- | --- | --- |
| **Hx** | **S_0_** | 0.00 | 2.66 |
|  | **S_1,40_** | 0.19 | 2.47 |
| **Tol** | **S_0_** | 0.00 | 2.58 |
|  | **S_1,40_** | 0.20 | 2.41 |
| **ACN** | **S_0_** | 0.00 | 2.53 |
|  | **S_1,40_** | 0.43 | 2.17 |
| **DMSO** | **S_0_** | 0.00 | 2.49 |
|  | **S_1,40_** | 0.41 | 2.16 |
